# Recombination analysis of *Human mastadenovirus C* whole genomes

**DOI:** 10.1038/s41598-019-38719-z

**Published:** 2019-02-18

**Authors:** Pierre Rivailler, Naiying Mao, Zhen Zhu, Wenbo Xu

**Affiliations:** WHO WPRO Regional Reference Measles/Rubella Laboratory and NHC Key Laboratory of Medical Virology and Viral Diseases, National Institute for Viral Disease Control and Prevention, Chinese Center for Disease Control and Prevention, 155 Changbai Road, Changping District, Beijing, 102206 People’s Republic of China

## Abstract

This study aims at analyzing all publicly available HAdV-C whole genome sequences (WGSs) and describes the genetic relationships between these genomes as well as identifies potential hotspots for recombination throughout the viral genome. In addition to the 4 prototypical genomic sequences, this analysis identified 20 HAdV-C WGSs which should be relevant for future recombination analysis of HAdV-C. This report confirmed the recombinogenic property of HAdV-C genomes and identified two main regions for breakpoints, within the hexon gene and around the fiber genomic region. No obvious recombination was detected between HAdV-Cs and non-human mastadenoviruses or non-C HAdVs. Finally, it highlighted the need for a surveillance of HAdVs in order to detect novel recombinant types that might represent health risks and develop possible prevention measures. Genetic analyses of recombination between recently collected HAdV-Cs and the assessment of their potential virulence are necessary steps towards the establishment of a surveillance of HAdVs in the future.

## Introduction

The human mastadenovirus (HAdV) is a non-enveloped, double-stranded DNA virus of the family *Adenoviridae* within the genus *Mastadenovirus*^1^. The viral capsid is composed of two types of capsomeres: the hexon and the penton (which itself consists of the penton base and the fiber)^1^. Hexon and fiber are involved in neutralization as well as hemagglutination inhibition for the latter^2^. HAdVs are divided into 7 species (HAdV-A to G) with 90 types (as of July 2018) based on biological properties, serum neutralization assays, and whole-genome sequencing analysis^3^. Recently, the HAdV working group established a nomenclature based on penton base, hexon and fiber sequences (PHF), and is continuously updating the nomenclature based on biological and genomics data (http://hadvwg.gmu.edu). As multiple studies revealed that HAdV was prone to intratypic recombination, the 9th International Adenovirus Meeting proposed to use whole genome sequences (WGSs) to characterize and name novel HAdV types^4^. The HAdV-C species comprises 6 types named 1, 2, 5, 6, 57 and 89. The prototypes for type 1, 2, 5 and 6 were collected in 1953 in the USA whereas the prototype for type 57 was collected in Azerbaijan (AZE) in 2001. According to the adenovirus working group, C89 has been identified in 2015 in Germany (http://hadvwg.gmu.edu).

As the number of HAdV-C WGSs grows, it becomes necessary to have a better idea of the genetic relationship between all these viral genomes. Among all the 102 WGSs available in GenBank (as of March 2018), only four (HQ003817, KR699642 MF315028 and MF315029) have been analyzed in terms of recombination^5–7^. The genomes HQ003817, (prototype of type 57) and KR699642 (strain CBJ113) were described in the context of the 4 prototype viruses collected in 1953 (type 1, 2, 5 and 6)^5,6^. We recently described putative recombination events for two viruses (BJ04 and BJ09) collected in China in 2012–2013 (MF315028 and MF315029)^7^. We attempted to perform the analysis in the context of recently collected HAdV-C viruses. However, the analysis was limited by the fact that the recombination events between other genomes were not described. This study aims at analyzing all publicly available HAdV-C WGSs and describes the genetic relationships between these genomes as well as identifies potential hotspots for recombination throughout the genome.

## Results

### Phylogenetic analysis

A phylogenetic network was originally constructed with 32 HAdV-C WGSs (tree in Supplementary Fig. S1, sequences listed in Table 1). The network was robust with a fit index greater than 97%. Four clusters were identified, representing 4 of the 5 types in HAdV-C. Cluster 1, 2, 5 and 6 contained the sequence AF534906 of the prototype of type 1, NC_001405 of type 2, AC_00008 of type 5 and FJ349096 of type 6, respectively. Three sequences were not included in any cluster, notably JX173080-2001-EGY, HQ003817-2001-AZE and KF268129-2005-USA. With the exception of type 5 cluster, all clusters contained several sequences featuring a similar phylogenetic profile: they are displayed as parallel lines and differ only at the very tip of a branch (for example, the 6 sequences LC068713-8 in type 6 cluster). The neighbor joining phylogenetic tree on the 32 WGSs confirmed that 7 sequences (LC068715, JX173085, KF268130, JX173086, JX173077, LC068720 and KF951595) were closely related to one of the remaining 25 sequences (identified with symbols in Supplementary Fig. S2). The sequence similarity was confirmed in phylogenetic trees across all genomic regions (Supplementary Fig. S2). These sequences did not involve any major recombination event and were not included in further analysis of recombination events (listed in Table 1). Only minor divergence was detected throughout these genomes, with pairwise genetic distance (called p-distance hereafter) of 0.003 or less to the sequence backbone (Supplementary Table S1).

**Table 1:** List of the 25 HAdV-C WGSs analyzed for recombination among the 43 WGSs downloaded from GenBank.

GenBank-ID^a^	Complete name or annotation	Country of collection	Year of collection	Informative in RDP4^b^	Recombination analysis^c^	Prototype	Reference
AF534906		USA	1953	Yes	Yes	Type 1	^23^
NC_001405		USA	1953	Yes	Yes	Type 2	^24^
AC_000008		USA	1953	Yes	Yes	Type 5	^25^
FJ349096		USA	1953	Yes	Yes	Type 6	^5^
LC068713	strain: 870550	Japan	1987	Yes	Yes		Iida *et al*., unpublished
KF268310	human/USA/Pitts_00109/1992/2[P2H2F2]	USA	1992	Yes	Yes		Madupu *et al*., unpublished
LC068714	strain: 930113	Japan	1993	Yes	Yes		Iida *et al*., unpublished
LC068715	strain: 940162	Japan	1994	Yes	No		Iida *et al*., unpublished
JX173078	human/ARG/A15812/2000/1[P1H1F1]	Argentina	2000	Yes	Yes		Madupu *et al*., unpublished
HQ003817	human/AZE/16700/2001/57[P1H57F6]	Azerbaijan	2001	Yes	Yes	Type 57	^5^
JX173081	human/EGY/E53/2001/2[P2H2F2]	Egypt	2001	Yes	Yes		Madupu *et al*., unpublished
JX173080	human/EGY/E13/2001/1[P1H1F1]	Egypt	2001	Yes	Yes		Madupu *et al*., unpublished
JX173079	human/ARG/A15932/2002/2[P2H2F2]	Argentina	2002	Yes	Yes		Madupu *et al*., unpublished
KX384959	strain T215/Ft Jackson South Carolina USA/2002	USA	2002	Yes	Yes		^26^
JX173084	human/USA/VT5544/2003/2[P2H2F2]	USA	2003	Yes	Yes		Madupu *et al*., unpublished
LC068716	strain: 1030787	Japan	2003	Yes	Yes		Iida *et al*., unpublished
JX173082	human/USA/VT384/2003/1[P1H1F1]	USA	2003	Yes	Yes		Madupu *et al*., unpublished
JX173083	human/USA/VT2672/2003/1[P1H1F1]	USA	2003	Yes	Yes		Madupu *et al*., unpublished
JX173085	human/USA/VT2612/2003/1[P1H1F1]	USA	2003	Yes	No		Madupu *et al*., unpublished
KF268130	human/USA/UFL_Adv2/2004/2[P2H2F2]	USA	2004	Yes	No		Madupu *et al*., unpublished
LC068717	strain: 1040264	Japan	2004	Yes	Yes		Iida *et al*., unpublished
LC068718	strain: 1040502	Japan	2004	Yes	Yes		Iida *et al*., unpublished
JX173086	human/USA/VT13862/2004/1[P1H1F1]	USA	2004	Yes	No		Madupu *et al*., unpublished
JX173077	human/ARG/A8649/2005/2[P2H2F2]	Argentina	2005	Yes	No		Madupu *et al*., unpublished
LC068720	strain: 1050158	Japan	2005	Yes	No		Iida *et al*., unpublished
KF268129	human/USA/UFL_Adv6/2005/6[P6H6F6]	USA	2005	Yes	Yes		Madupu *et al*., unpublished
JX423389	human/USA/ak31_AdV6/2007/6[P6H6F6]	USA	2007	Yes	Yes		Madupu *et al*., unpublished
KF268199	human/USA/UFL_Adv5/2008/5[P2/H5/F5]	USA	2008	Yes	Yes		Madupu *et al*., unpublished
KR699642	strain CBJ113	China	2009	Yes	Yes		^6^
MF315028	human/CHN/BJ04/2012/[P1/H2/F2]	China	2012	Yes	Yes		^7^
KF951595	strain DD28	China	2013	Yes	No		An *et al*., unpublished
MF315029	human/CHN/BJ09/2013/[P1/H2/F2]	China	2013	Yes	Yes		^7^
AY601635	strain NHRC Ad5FS 7151	USA		Yes	No^d^		Tibbetts *et al*., unpublished
AC_000007	same as NC_001405	USA	1953	No			^24^
J01917	same as NC_001405	USA	1953	No			^24^
HQ413315	Tonsil 99	USA	1953	No			^27^
AC_000017	same as AF534906	USA	1953	No			^23^
M73260	same as AC_000008	USA	1953	No			^25^
LC068712	strain: 870550	Japan	1987	No			Iida *et al*., unpublished
KF268127	human/USA/CL_42/1988/5[P5H5F5]	USA	1988	No			Madupu *et al*., unpublished
KF429754	human/USA/Pitts_00149/1990/5[P5H5F5], strain F268310	USA	1990	No			Madupu *et al*., unpublished
LC068719	strain: 1050156	Japan	2005	No			Iida *et al*., unpublished
AY339865				No			

^a^The genomes were sorted based on the columns “informative in RDP4”, “prototype” and “year of collection”.

^b^Only WGSs identified as informative in RDP4 were analyzed.

^c^The genomes closely related to another genome of the dataset based on phylogenetics and genetic distance and not involved in any major recombination event were not selected for recombination analysis.

^d^This genome was not selected because the collection year was unknown.

**Figure 1:**
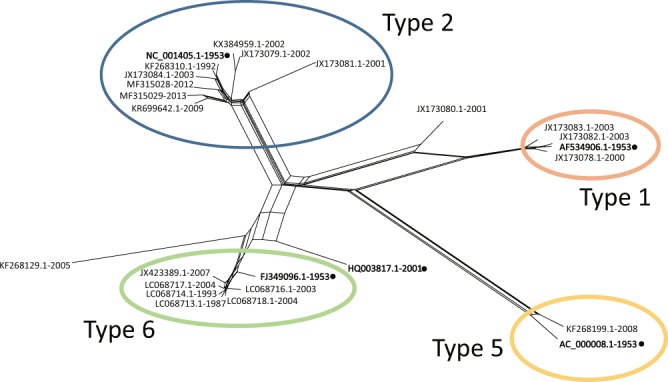
Phylogenetic network built with 25 HAdV-C WGSs. The clusters corresponding to type 1, 2, 5 and 6 are shown with different colors, type 1 in pastel pink, type 2 in blue, type 5 in pastel yellow and type 6 in pastel green. The sequences corresponding to the prototype viruses of type 1, 2, 5, 6 and 57 are shown in bold face and a dot.

The remaining 25 sequences were further analyzed for any potential recombination events (listed in Table 1). The phylogenetic network featured parallel branches indicating that the evolution was not linear and involved multiple recombination events (Fig. 1). The phylogenetic network appeared distorted due to 4 outlier sequences: JX173080-2001-EGY related to the type 1 network, JX173081-2001-EGY related to the type 2 network and sequences KF268129-2005-USA and HQ003817-2001-AZE which are related to type 6 network. A SplitsTree analysis was further implemented without these 4 outlier sequences (Supplementary Fig. S3). The phylogenetic network appeared tree-like suggesting that all the sequences were related and involved in multiple recombination events between each other.

### Recombination map based on phylogeny and genetic distance

**Figure 2:**
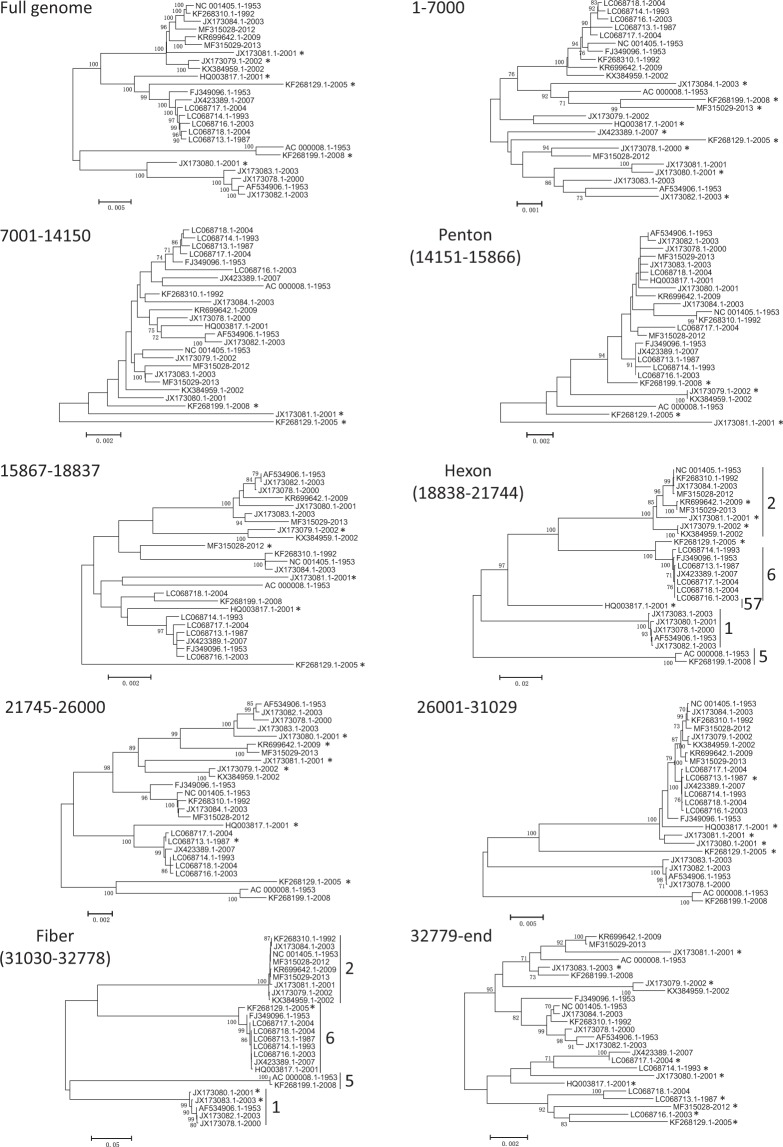
Neighbor joining phylogenetic trees of 25 HAdV-Cs. WGS as well contiguous regions were analyzed. The genomic region used to build the tree is shown for each tree. Type designations for the hexon and fiber genes are indicated by brackets. The sequences identified as outliers based on the p-distances from Supplementary Table S1 are identified with an asterisk. Bootstrap values greater than 70% are shown.

The recombination events were analyzed using two strategies, a manual approach using phylogenetics and genetic distances on one hand and a more automatic approach using the RDP4 software package on the other hand. The phylogenetic analysis was done on WGS as well as contiguous genomic regions including penton, hexon and fiber genes (Fig. 2). Pairwise p-distances were computed for the corresponding trees (Supplementary Tables S1–11) and analyzed following the algorithm presented in Supplementary Fig. S4. We arbitrarily set the threshold for the genetic distance at 0.005, a p-distance greater than 0.005 characterizing an outlier sequence. However, we allowed certain flexibility on the threshold depending on the p-distance and phylogenetic profiles throughout the genome. For example, even though the pairwise p-distance between KR699642-2009 and KF268130-2004 is 0.007 at the WGS level, KF268130-2004 was considered the backbone of KR699642-2009 because KR699642-2009 had a p-distance with KF268130-2004 less than 0.005 for 4 of the 9 analyzed genomic regions. The overall mean distance between WGSs is 0.032 and, at the genomic region level, the highest overall mean distances are found with the fiber gene (0.183) and the hexon gene (0.099) (Supplementary Table S12). This is  due to the fact that fiber and hexon genes are highly divergent with distinct types, 4 for fiber and 5 for hexon. However, the distance within types is <0.005 except for hexon type 2 (0.013) and fiber type 1 (0.006) (Supplementary Tables S7 and S10). Based on these values, considering a pairwise p-distance of 0.005 to identify outlier sequence seems reasonable. As previously described, a threshold of 0.005 resulted in the identification of 7 genomes that were not likely to be involved in any recombination event.

**Figure 3:**
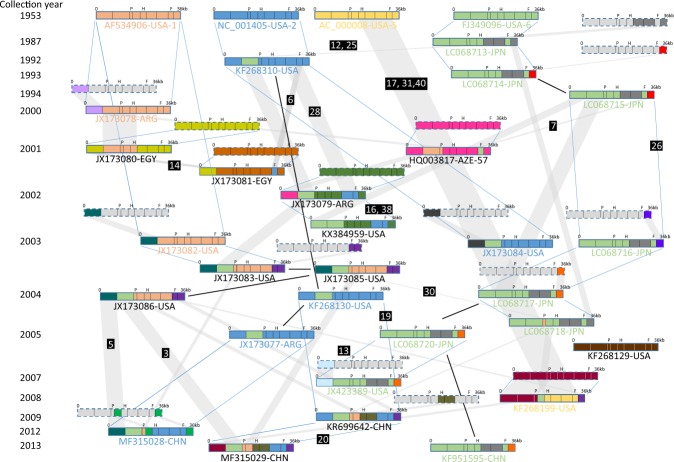
Graphic representation of the genetic relationships between 32 HAdV-Cs. Each HAdV-C genome is represented in scale, as a colored box with 9 portions corresponding to the 9 genomic regions used in this analysis. The start and the end of the genome as well as the regions corresponding to the penton (P), hexon (H) and fiber (F) genes are indicated. The genomes of the prototype of type 1, 2, 5 and 6 are shown on the top. Type 1, 2, 5 and 6 are represented in pastel pink, blue, pastel yellow and pastel green, respectively. The other colors are indicating sequences with unknown origin. Hypothetical genomes are shown as dotted line boxes. Hypothetical genomic regions not involved in any recombination event are shown in light grey. The relationship between genomes was established based on phylogenetic trees (Fig. 2 and Supplementary Fig. S2) and p-distances (Supplementary Table S1). The relationship between genomes is indicated as follows: the related genomes that are not involved in major recombination events are linked with a thick black line (for example, KF268310 and KF268130). A recombinant virus is linked to its major parental genome (or backbone) by two parallel straight lines. Finally, the genomic sequences involved in recombination are linked by a parallelogram shaded in grey. The names of the genomes sharing the same genotype as a prototype virus are shown in the same color as the prototype name. The number of the RDP event confirming the genetic relationship is shown in a black box. The collection year is indicated on the left side of the figure.

The analysis of the phylogenetic trees in combination with the genetic distances allowed to draw a recombination map (Fig. 3). It is important to note that this map is a simplified way of looking at recombination potential as only major events occurring between the analyzed genomic regions were identified. In contrast, events occurring within these genomic regions were not identified. Furthermore, this representation did not consider breakpoints. It only identified genomic regions of potential genomes that were related, i.e. likely involved in recombination events. The map featured 32 genomes, including those that were not involved in major recombination events. The map was color coded with the 4 prototypes at the top of the map, each with a different color, pastel pink for type 1, pastel blue for type 2, pastel yellow for type 5 and pastel green for type 6. Any other color featured in the map represented a sequence from a hypothetical genome. These hypothetical genomes were created based on p-distances (>0.005) and phylogenetic analyses (Fig. 2, Supplementary Table S1). The map featured 3 types of genomic relationships, represented by a thick black line, parallel blue lines or a shaded parallelogram. Similar genomes were linked by a thick line as their evolution did not involve any major recombination event (for example, KF268310 and KF268130, with p-distance ≤ 0.002 throughout the genome). Related genomes were linked by parallel lines. They were identified in the tree featuring WGSs (Fig. 2). These genomes could be considered as major parent or backbone of recombinant viruses (for example, AF534906-1953 is likely to be the backbone of JX173078-2000; p-distance is 0.003 at the WGS level). Finally, genomes were linked by shaded parallelograms when they were involved in a recombination event. These genomes could be considered as minor parents. For example, the genomic regions from 7001 to 21744 of JX173078-2000-ARG and JX173080-2001-EGY are related with p-distance ≤ 0.005.

As the phylogenetic network showed, KF268129-2005 is completely isolated as it is not related to any known sequences (Fig. 3). Whereas most of the recombinant genomes involved one recombination event, 10 recombinants involved several recombination partners, namely JX173081, JX173079, HQ003817, JX173083, LC068717, LC068718, KF268199, KR699642, MF315028 and MF315029. For example, the JX173081-EGY-2001  genome was predicted to consist of an unknown backbone combined with the genomic region 1-7000 of JX173080-2001-EGY and the fiber gene of KF268310-1992-USA. The relationship between the 32 genomes was established with the help of 16 unknown genomes (dotted line boxes in Fig. 3). In order to exclude any potential involvement of adenovirus sequence of a non-human host, phylogenetic analysis of the 32 HAdV-C WGS was performed in the context of two non-human mastadenovirus WGS, from chimpanzee (CS138463) and bonobo (HC191035) (Supplementary Fig. S5). The neighbor joining phylogenetic trees on WGS as well as on the 9 contiguous genomic regions did not show any evidence for recombination between HAdV-C sequences and non-human adenovirus sequence. Similarly, HAdV-C sequences were analyzed in the context of non-C HAdV sequences in order to assess for any potential recombination event between HAdV-C and HAdV of another species (Supplementary Fig. S6). Once again, the neighbor joining phylogenetic trees on WGS as well as on the 9 contiguous genomic regions did not show any evidence for recombination between HAdV-C sequences and sequences of HAdV of another species. Whereas most of the unknown genomes were predicted to be involved in the recombination of one single genomic region, 5 of these unknown genomes could be considered as the entire backbone of recombinant viruses (dotted line box filled with one single color in Fig. 3). It was the case for the Egyptian viruses, JX173080 and JX173081, the Azerbaijani virus HQ003817 also known as the prototype for type 57, one Argentinian virus, JX173079 and one American virus, KF268199. Whereas most genomes were generally involved in a single recombination event, 3 genomes were involved in several recombination events. This was the case for KF268310, LC068715 and JX173086. For example, it was predicted that LC068715-1994-JPN was involved in three recombination events: the genomic region 1-7000 in KX384959-2002-USA, the genomic region 7001-14150 in LC068717-2004-JPN and the fiber gene in HQ003817-2001-AZE. Finally, considering the viruses collected recently, KR699642 in 2009 and the related virus MF315029 collected in 2013, it is clear that these genomes cannot be identified by their type any longer, as they are a mosaic of sequences of different origin. In the recombination map (Fig. 3), penton (P), hexon (H) and Fiber (F) loci each feature a different color meaning that these three genes have a different evolutionary history. For example, the genome KR699642-2009-CHN was predicted to have a backbone related to KF268130-2004-USA (closely related to type 2 and 6) combined with a penton gene from LC068718-2004-JPN (related to type 1), the genomic regions 15867-18837 and 32779-end from JX173086-2004-USA as well as a genomic region 18838-26000 of unknown source.

HAdV-C genomes are identified by the type of their penton (P), hexon (H) and fiber (F) genes. This is the basis of the PHF nomenclature and could be considered as a genotype. The prototype viruses are defined by penton, hexon and fiber genes of a same type. For example, the prototype of type 1, AF534906 is defined by the P1H1F1 genotype. Among the 28 analyzed WGSs, 15 share the same genotype of a prototype (sequence name in color in Fig. 3). For example, 2 genomes (JX173078-2000-ARG and JX173082-2003-USA) have a P1H1F1 genotype. The genome KF268129 is outlier for the penton, hexon and fiber gene with p-distances of 0.012, 0.017 and 0.018, respectively (Supplementary Table S1). In this analysis, a sequence with the lowest p-distance > 0.005 against any other sequences of the dataset was considered outlier. Four genomes have an outlier penton sequence, namely JX173081.1 (p-distance of 0.026), JX173079.1 (0.013), KX384959.1 (identical to JX173079.1) and KF268199.1 (0.005). The latter was considered outlier as the node was supported by 94% bootstrap. Given that the penton tree featured only 5 significant bootstrap values, we considered that the supported nodes should be given much attention. Six genomes had an hexon gene that was less conserved than the others: in addition to HQ003817.1 which had been already identified as the prototype C57, JX173081.1 (p-distance of 0.022), JX173079.1 (0.021), KX384959.1 (identical to JX173079.1), KR699642.1 (0.012) and MF315029 (identical to KR699642.1). Finally, 4 genomes had a less conserved fiber gene: JX173080.1 (p-distance of 0.009), JX173083.1 (0.007), JX173085.1 and JX173086.1 (both similar to JX173083.1). Interestingly, these divergences were also featured in the phylogenetic network (Fig. 1). HQ003817, with a highly divergent hexon gene, which was the basis of the type 57 designation, and a fiber gene of type 6, was clearly an outlier but related to the network corresponding to type 6. The other 5 genomes with a divergent hexon gene and a fiber gene of type 2 (JX173081.1, JX173079.1, KX384959.1, KR699642.1 and MF315029) were found on type 2 cluster but they were part of 2 sub-networks separated from the main trunk. JX173080 with a divergent fiber gene but an hexon of type 1 was an outlier of the type 1 network. The remaining three genomes with a divergent fiber gene and an hexon gene of type 1 (JX173083.1, JX173085.1 and JX173086.1) were also part of the type 1 cluster but separated from the other type 1 sequences (Supplementary Fig. S1).

### Recombination hot spots based on automatic analysis

**Figure 4:**
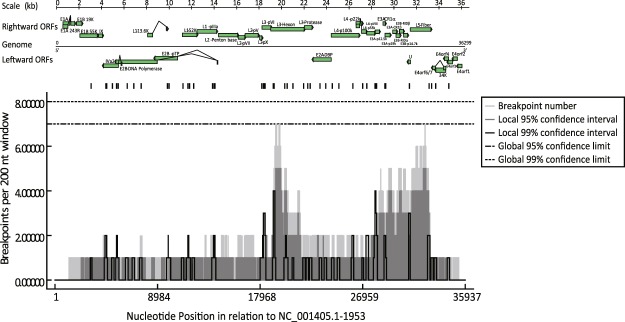
Breakpoint distribution across HAdV-C genome. The distribution plot was generated by RDP4 using 40 recombination events. A genomic map is shown above, featuring the genome as a straight line, the rightward ORFs above this line and the leftward ORFs below this line. ORFs are represented as thick arrows whereas introns are represented as a broken line. The genome NC_001405 was used for the annotation and numbering.

A more systematic approach was followed using the RDP4 software. One hundred seventeen recombination events were identified (Supplementary Table S13). As RDP4 first identified the most evident recombination events, we chose to focus on the 40 first recombination events, which were identified by more than 3 algorithms (Supplementary Table S13). Among the 40 best recombination events, only 55 breakpoints were mapped as some breakpoints were undetermined due to a weak signal and overlap with subsequent recombination events. RDP4 generated a distribution plot of breakpoints based on these 40 recombination events (Fig. 4). Breakpoints were predicted to occur across the genome but there were two major regions where breakpoints could occur, around positions 19640 and 32300. Whereas the first region was relatively delimited with a sharp peak, from 19186 to 20258, which corresponds to the hexon gene (18838-21744), the second region was less defined, from 28263 to 32719, which contains the genes coding for the glycoprotein (gp) CR1α, gp19K, gpCR1β, RIDα, RIDβ, control protein E3 14.7 K, protein U, and fiber protein. An association test was performed to assess whether there was any evidence of breakpoint locations being influenced by any genomic features. Thirty-eight genes/exons were identified in the NC_001405 genome (Supplementary Table S14). Among these 38 genomic features, 4 had an unusual high number of predicted breakpoints compared to the rest of the genome with a probability of it being due by chance less than 1% (p-value < 0.01): IVa2-e2, [second exon of IVa2] (p-value of 0.002), E2B1, [the most leftward E2B ORF] (0.009), L3-1, [the most leftward L3 ORF] (0.006) and E3A-1, [the most leftward E3A ORF] (0.003) (Supplementary Table S15). For example, IVa2 exon is 1336 nt long (3.8% of the coding potential of the genome) and 4 breakpoints were predicted in that locus. If a similar breakpoint distribution was observed in all the ORFs, 105 breakpoints would have been expected, which is half of what has been predicted by RDP4. Finally, the breakpoints were significantly more localized at the end of the ORFs with a p-value of 0.001.

### Macro- and micro-evolution of *Human mastadenovirus C*

Phylogenetic analysis focused on macro-evolution as it concerned large genomic regions whereas RDP analysis focused on micro-evolution as it was based on sliding window analysis. In order to test whether RDP predictions were supported by phylogenetic analysis, the 40 first RDP events were assessed based on several criteria, namely breakpoints, sequences involved in the recombination event as well as p-values supporting the predictions (Table 2, Supplementary Table S13). None of the 40 recombination events predicted by RDP involved a defined trio recombinant/minor parent/major parent. Thus, in all cases, at least one partner was identified as 2 or more sequences. Among the 40 RDP events, 18 were confirmed by phylogenetic analysis and were identified on the recombination map (event number in black box in Fig. 3). For example, event 3 concerned the genomes MF315029-2013, JX173083-2003 and JX173081.1-2001. The estimated breakpoints were located at 4505 and 19200 meaning that the trees corresponding to the genomic region 1 to 18837 on one hand and the trees for the rest of the genome should give a different pattern for MF315029-2013, JX173083-2003 and JX173081.1-2001. Indeed, in the tree concerning the genomic region 7001-14150, MF315029-2013 and JX173083-2003 formed a cluster of 3 sequences supported by a bootstrap of 100%. In contrast, the fiber tree (31030-32778) showed that MF315029-2013 and JX173081.1-2001 were of type 2 whereas JX173083-2003 was of type 1. Thus, phylogenetic analysis confirmed a recombination between MF315029-2013, JX173083-2003 and JX173081.1-2001. The RDP events non-featured in the recombination map concerned undefined relationships either due to multiple sequences identified as recombinant/minor/major parent or due to unknown parent (Table 2, Supplementary Table S13). For example, event 1 was difficult to assess as it involved 21 sequences as potential minor parent and 6 sequences as potential major parent. Similarly, event 2 was not mapped as it involved unknown minor parent and 10 sequences as potential major parent.

**Table 2:** List of the 40 first RDP events.

Event #^a,b^	Begins^c^	Ends^c^	Recombinant Sequence(s)^d,e^	Minor Parental Sequence(s)^e^	Major Parental Sequence(s)^e^	RDP^f^	GENECONV^f^	Bootscan^f^	Maxchi^f^	Chimaera^f^	SiSscan^f^	3Seq^f^	Map^g^
1	28060	31054	JX173080.1-2001	21 sequences	6 sequences	7.34E-186	1.25E-189	1.03E-183	1.60E-49	3.40E-51	5.81E-53	1.65E-12	no
2	27327	31044	KF268129.1-2005	10 unknown sequences	10 sequences	9.04E-117	1.03E-117	1.05E-126	2.12E-46	2.02E-47	1.52E-55	1.10E-12	no
**3**	**4505**	**19200**	**^MF315029-2013; KR699642.1-2009**	**4 sequences**	**JX173081.1-2001**	**5.43E-118**	**3.96E-114**	**6.66E-66**	**3.65E-31**	***3.99E-09***	**3.84E-29**	**2.20E-12**	**yes**
4	27327	31052	11 sequences	10 sequences	6 sequences	5.87E-98	4.50E-115	3.58E-112	3.26E-37	2.44E-28	1.45E-56	5.51E-13	no
**5**	4090*	**18370**	**^MF315028-2012**	**4 sequences**	**JX173081.1-2001**	**1.01E-101**	**5.45E-88**	**1.65E-78**	**4.53E-27**	**5.73E-15**	**8.97E-27**	**5.51E-13**	**yes**
**6**	**28005**	**32999**	**6 sequences**	**JX173081.1-2001**	**1 unknown sequence**	**2.53E-67**	**1.44E-58**	**7.44E-66**	**4.69E-37**	**1.61E-15**	**3.80E-94**	**1.01E-77**	**yes**
**7**	**26951**	**32743**	**HQ003817.1-2001**	**KF268129.1-2005; FJ349096.1-1953**	**7 sequences**	**3.04E-55**	**3.04E-67**	**3.53E-71**	***3.99E-05***	**5.16E-15**	**9.19E-47**	***1.58E-03***	**yes**
8	4435	19128	^JX173081.1-2001	3 unknown sequences	JX173079.1-2002; KX384959.1-2002	6.57E-67	1.74E-47	6.97E-36	1.68E-24	5.61E-10	3.36E-20	*1.07E-04*	no
9	5504	18363	^KF268129.1-2005	6 unknown sequences	11 sequences	5.65E-63	1.25E-48	3.76E-26	1.13E-25	*1.08E-07*	3.05E-12	*2.23E-05*	no
10	22362	26094	^KF268129.1-2005	2 unknown sequences	FJ349096.1-1953	NS	4.47E-61	*4.46E-07*	3.30E-21	7.40E-21	1.29E-18	1.10E-12	no
11	20327	21795	11 sequences	3 sequences	7 sequences	1.23E-43	8.38E-55	5.41E-50	1.64E-16	2.37E-14	1.17E-27	5.51E-13	no
**12**	**23242**	**26073**	**FJ349096.1-1953**	**JX173077.1-2005; KR699642.1-2009**	**9 sequences**	**NS**	**4.39E-41**	**NS**	**3.52E-14**	**5.20E-13**	**1.70E-12**	**1.10E-12**	**yes**
**13**	596*	**11183**	**^JX423389.1-2007**	**1 unknown sequence**	**7 sequences**	**3.64E-15**	**1.90E-26**	**9.03E-31**	**4.81E-13**	**2.70E-12**	***3.62E-09***	**7.69E-35**	**yes**
**14**	1*	4434*	**^JX173081.1-2001**	**JX173080.1-2001**	**MF315029-2013; KR699642.1-2009**	**1.55E-41**	**3.81E-35**	**3.34E-34**	**9.48E-13**	**7.47E-14**	**6.81E-14**	**1.65E-12**	**yes**
15	23695	28059*	JX173081.1-2001	7 sequences	MF315028-2012	NS	2.89E-10	*3.32E-03*	1.21E-17	1.26E-14	*9.49E-04*	1.65E-12	no
**16**	3067*	**7533**	**^KX384959.1-2002**	**13 sequences**	**JX173079.1-2002**	**9.56E-41**	**3.71E-26**	**2.69E-25**	***2.11E-09***	**3.40E-10**	***5.23E-08***	**1.13E-16**	**yes**
**17**	**13801**	**18289**	**^KF268199.1-2008**	**7 sequences**	**AC_000008.1-1953**	**1.30E-39**	**8.29E-23**	**9.77E-21**	***8.41E-08***	***1.85E-08***	***8.05E-07***	**2.75E-12**	**yes**
18	32764	34473	8 sequences	3 sequences	JX173080.1-2001	2.40E-39	9.91E-30	1.87E-39	*2.93E-09*	*2.15E-09*	2.27E-10	1.65E-12	no
**19**	**22109**	**24308**	**^KR699642.1-2009; MF315029-2013**	**7 sequences**	**KF268310.1-1992**	**1.46E-38**	**3.03E-22**	***1.61E-03***	***5.52E-07***	***8.96E-08***	***1.11E-04***	**NS**	**yes**
**20**	291*	4151*	**MF315029-2013**	**11 unknown sequences**	**KR699642.1-2009; JX173079.1-2002**	**4.27E-36**	**1.38E-31**	**1.32E-37**	**2.40E-10**	***3.96E-09***	***1.54E-07***	**1.10E-12**	**yes**
21	171*	4504*	KF268129.1-2005	3 unknown sequences	8 sequences	3.06E-32	3.21E-28	8.74E-19	1.13E-11	2.32E-10	*1.44E-05*	1.10E-12	no
22	6874	13947	5 sequences	JX173079.1-2002	JX173082.1-2003	7.73E-31	4.06E-17	3.92E-20	*2.38E-05*	*5.14E-06*	*3.41E-06*	2.20E-12	no
23	28137	28931	^AC_000008.1-1953; KF268199.1-2008	1 unknown sequence	6 sequences	2.27E-24	1.09E-29	3.72E-16	*9.70E-08*	8.39E-13	9.30E-23	*1.29E-05*	no
24	3163	6835*	^JX173079.1-2002	AF534906.1-1953	4 unknown sequences	2.25E-28	4.61E-17	1.94E-28	*1.18E-04*	*9.24E-05*	8.19E-23	3.85E-12	no
**25**	159*	**9848**	**^JX173084.1-2003**	**7 unknown sequences**	**3 sequences**	**9.12E-15**	**4.79E-13**	***2.08E-06***	**1.00E-14**	**8.40E-16**	***1.88E-07***	**6.01E-28**	**yes**
**26**	**10021**	**11763**	**^LC068716.1-2003**	**5 unknown sequences**	**8 sequences**	**2.51E-27**	**9.55E-22**	**5.95E-23**	***1.64E-06***	***1.52E-05***	***1.81E-03***	**1.65E-12**	**yes**
27	950*	10020*	^HQ003817.1-2001	5 unknown sequences	7 sequences	*4.00E-08*	*3.18E-08*	*0.019766271*	*2.19E-09*	*3.13E-06*	2.40E-13	5.03E-37	no
**28**	18697*	**24849**	**^JX173079.1-2002; KX384959.1-2002**	**7 sequences**	**NC_001405.1-1953**	**4.50E-19**	***5.28E-03***	***4.14E-04***	***8.13E-05***	***2.20E-05***	**1.44E-48**	**2.20E-12**	**yes**
29	34781*	35890*	5 sequences	7 unknown sequences	9 sequences	1.97E-20	2.31E-24	5.52E-22	*2.93E-04*	*3.67E-03*	*1.75E-03*	*2.23E-09*	no
**30**	**14016**	**18091**	**^LC068718.1-2004**	**7 sequences**	**8 sequences**	**1.02E-24**	**1.05E-12**	**9.87E-14**	***7.44E-05***	***1.55E-04***	**NS**	***2.61E-09***	**yes**
**31**	**5067**	**11627**	**^AC_000008.1-1953**	**3 unknown sequences**	**KF268199.1-2008**	**1.02E-23**	**NS**	**NS**	***2.53E-05***	***1.43E-06***	**NS**	**2.20E-12**	**yes**
32	18364*	19197	^AC_000008.1-1953	10 sequences	1 unknown sequence	5.47E-21	9.04E-13	4.26E-20	*4.29E-04*	*1.75E-04*	NS	1.44E-10	no
33	28155	28899	^KF268129.1-2005	5 sequences	6 unknown sequences	1.99E-19	NS	*1.27E-06*	*9.75E-09*	3.50E-11	6.15E-18	*1.29E-05*	no
34	18419*	19197	^KF268129.1-2005	1 unknown sequence	10 sequences	6.65E-19	*1.48E-08*	4.97E-19	*7.73E-04*	*2.94E-04*	NS	*3.23E-07*	no
35	33322	34308*	8 sequences	1 unknown sequence	7 sequences	2.02E-18	1.59E-17	9.24E-19	*3.44E-03*	*2.50E-04*	*1.20E-02*	*3.54E-08*	no
36	34970*	35937*	^KF268129.1-2005	5 unknown sequences	5 sequences	1.59E-14	*3.60E-07*	9.58E-18	*1.12E-02*	*2.90E-03*	NS	NS	no
37	20154	20845	^HQ003817.1-2001	5 sequences	7 sequences	1.43E-10	5.59E-11	*1.81E-08*	*3.58E-02*	*4.06E-03*	6.64E-17	5.51E-13	no
**38**	7594*	**12137**	**^KX384959.1-2002**	**3 unknown sequences**	**JX173079.1-2002**	**8.00E-16**	***5.33E-03***	***6.00E-05***	***4.97E-05***	**NS**	**NS**	***2.06E-02***	**yes**
39	5397	6297	JX173083.1-2003	13 sequences	3 sequences	NS	1.52E-12	1.00E-15	NS	NS	NS	*1.07E-03*	no
**40**	34560*	35935*	**^KF268199.1-2008**	**12 sequences**	**AC_000008.1-1953**	**3.66E-13**	**3.59E-10**	**1.14E-14**	**NS**	**NS**	**NS**	**1.65E-12**	**yes**

^a^RDP events are listed in Supplementary Table S13.

^b^RDP events that are identified in recombination map in Fig. 3 are shown in bold.

^c^Undetermined breakpoints are shown with “*”.

^d^The potentially misidentified recombinants are indicated with ^.

^e^In cases where more than 2 sequences are listed as potential candidate for a recombination event, the number of listed sequences is indicated.

^f^p-values > 1E-10 are italicized.

^g^RDP events shown in bold are featured in the recombination map in Fig. 3.

## Discussion

The aim of this study was to better understand the relationship between all publicly available HAdV-C WGSs. Our analysis identified several categories of sequences which should facilitate future recombination analyses of HAdV-C genomes. First, among the 102 publicly available WGSs, 60 were not suitable for analysis as they were incomplete or from viruses that were patented, modified or collected from a non-human species. Second, 17 sequences were uninformative relative to recombination events. Among the 25 remaining sequences, 4 were from the prototype viruses of type 1, 2, 5 and 6 collected in 1953. One sequence, from virus KF268129-2005-USA, was very different from the other sequences. The remaining 20 sequences are likely to be relevant for future analyses of recombination event between HAdV-C viruses and we would suggest to use those sequences to describe HAdV-C that will be collected in the future.

Several studies described adenovirus genomes with genetic elements from both human and primate adenoviruses^8–10^. Furthermore, Chen *et al*. showed that an adenovirus collected in New World monkeys can infect humans^11^. Interestingly, the current study does not provide any evidence for a recombination between HAdV-C and non-human adenoviruses or non-C species HAdV. The currently publicly available WGSs cannot help to understand most of the genetic relationships between HAdV-Cs outside the well-studied penton/hexon/fiber genes. A few reasons might explain this situation. Many HAdV-C genomes have only been been partially sequenced in order to analyze the penton, hexon and fiber genes but only a few WGSs are currently available for obvious financial reasons. It is possible that some of the currently available viruses might help to understand some genetic relationship. Because it is not possible or even reasonable to fully sequence all viruses, we would suggest to randomly select viruses focusing on country distribution. Among the 42 available WGSs, more than half (23) concern viruses collected in the USA, the remaining WGSs concern viruses from 5 other countries (Japan (8 genomes), China (4), Argentina (3), Egypt (2) and Azerbaijan (1)). HAdV-C data from more than 30 countries is available from GenBank so it might be worth increasing the spectrum of countries from where HAdV-C WGSs have been generated. Another reason to explain the missing data is likely to be a limited surveillance. Many infections are likely un-noticed or un-notified. A better surveillance would obviously increase the amount of data available for analysis and allow a more reliable understanding of the genetic relationship between circulating viruses. Even though a recent study by Ismail *et al*. suggested that HAdV-Cs were more stable than other HAdVs, the current analysis provides evidence of the recombination process molding the dynamic evolution of this species of HAdV and should be a good argument for better surveillance programs that at least consider hexon and fiber rather than only the hexon region^12^. Finally, it is possible that a high p-distance, which is characterizing an outlier sequence, might be the consequence of recombination events. For example, sequence C could be the result of the recombination between a known sequence A and a known sequence B and the resulting mosaicism could lead to a higher p-distance.

Among the 32 analyzed WGSs, one genome, KF268129-2005-USA, appeared very different from the others. This genome has been reported to be of P6H6F6 genotype in GenBank. However, the current analysis shows a p-distance greater than 0.015 from the closest type (HAdV-C6) for hexon and fiber gene which might be a good argument for a new type designation^13^. Independently of the designation issue, the relatively strong divergence of this sequence might be once again the consequence of a limited surveillance for HAdVs.

This analysis was mainly based on phylogenetic analysis of 9 contiguous genomic regions. As previously discussed by Walsh *et al*., the high similarity of HAdV-C genomes result in a low phylogenetic signal and it is necessary to analyze a relatively large genomic region in order to detect some sequence variation^5^. The analysis strategy using the 9 contiguous genomic regions was previously described^7^. These regions were arbitrarily designed, focusing on the penton, hexon and fiber genes as well as the other genomic regions in order to limit the number of analyzed regions but also have an overview of the entire genome. A compromise needed to be found between detecting most recombination events with low confidence or detecting only the major ones with high confidence. Our analysis tried to strike a balance by using on one hand, phylogenetics on large genomic regions, to visually detect obvious recombination events and, on the other hand, the RDP4 software, using a sliding window strategy, to automatically detect recombination events and get some statistics on breakpoint distribution. The breakpoint distribution identified two hotspots corresponding to the hexon gene and the genomic region upstream of the fiber gene including the fiber gene *per se*. Such outcome was somewhat expected as recombination between genomes involving hexon and fiber genomic regions were previously described for HAdV-C genomes^5,6,14^. The consequence of intratypic recombination on HAdV-C pathology remains unclear. A more unexpected result was the fact that no other genomic region seemed to be consistently involved in any recombination events suggesting that the penton sequence was not a good genomic marker. As we and other authors previously noted, the current phylogenetic analysis of the penton sequence confirmed this statement as only 5 nodes of the phylogenetic tree had a significant bootstrap value, greater than 70%, suggesting that most of the penton sequences were not highly divergent^7,12^.

In conclusion, this analysis identified 20 HAdV-C WGSs which should be relevant for future recombination analysis of HAdV-C. This report confirmed the recombinogenic property of HAdV-C genomes and identified two hotspots for breakpoints, within the hexon gene and around the fiber genomic region. Finally, it highlighted the need for a surveillance of HAdVs in order to detect novel recombinant types that represent potential health risk and develop possible prevention measures. Genetic analyses of recombination between recently collected HAdV-Cs and the assessment of their potential virulence is a necessary step towards the establishment of a surveillance of HAdVs in the future.

## Material and Methods

### Dataset

One hundred and two HAdV-C WGSs were downloaded from GenBank. The viral genomes that were patented, modified, or incomplete or from viruses collected from a non-human species were discarded. The 43 remaining WGSs were analyzed (Table 1). Ten WGSs were identified as uninformative by the Recombination Detection Program RDP4^15^. One genome was discarded as the collection year was unknown. The remaining 32 WGSs were aligned with MAFFT version 7^16^. Twenty-five of these sequences were analyzed for recombination event (listed in Table 1). The viruses were identified by their GenBank ID, year and country of collection in the manuscript. In order to assess for any potential recombination event between the genomes of HAdV-C and the genomes of non-human mastadenoviruses, two non-human mastadenovirus WGS were downloaded from GenBank, CS138463 from chimpanzee adenovirus and HC191035 from bonobo adenovirus^10^. Similarly, in order to assess for any potential recombination event between the genomes of HAdV-C and the genomes of non-C HAdV, 76 WGS of species of HAdV-A, B, D, E, F or G were downloaded from GenBank.

### Phylogenetic analysis

WGSs alignment was split into 9 pieces in order to monitor potential recombination events within the penton base, hexon and fiber knob genes as well as the rest of the genome as previously described^7^. The HAdV-C genome was divided as follows, 1-7000, 7001-14150, 14151-15866 (Penton gene), 15867-18837, 18838-21744 (Hexon gene), 21745-26000, 26001-31029, 31030-32778 (Fiber gene) and 32779-end. The numbering is based on NC_001405 genome. The sequences were used to generate neighbor joining trees with the MEGA 6 software and the maximum composite likelihood nucleotide substitution model^17,18^. The phylogenetic inference was tested with the bootstrap method with 1000 replications^19^. Bootstrap values greater than 70% were indicated. Pairwise p-distances were computed in MEGA 6 (Supplementary Tables S2–S11). The p-distance is the proportion (*p*) of nucleotide sites at which two sequences being compared are different. It is obtained by dividing the number of nucleotide differences by the total number of nucleotides compared. A threshold for divergence was arbitrarily set up at 0.005. Two sequences having a p-distance less than 0.005 would be considered related sequences. A sequence featuring a p-distance greater than 0.005 to all sequences in the dataset would be considered outlier sequence. Outliers were visually checked in phylogenetic trees and identified with an asterisk (Fig. 2).

### Recombination analysis

WGSs were graphically analyzed with SplitsTree4 using default parameters^20^. The recombination events were analyzed using two strategies, a manual approach using phylogenetics and genetic distances on one hand, and a more automatic approach using the RDP4 software package on the other hand. Pairwise p-distances were analyzed following the algorithm presented in Supplementary Fig. 4. Potential recombination events were also identified by one of the 7 tested algorithms (RDP, GENECONV, Chimaera, MaxChi, BootScan, SiScan and 3Seq) within the RDP4 package^15^. Recombination breakpoint distribution plot was generated with RDP4 using a 200nt sliding window^21^. Clustering tests were also generated by RDP4 using a genomic annotation of NC_001405 edited in Artemis (Table S13)^22^.

## Supplementary information

Supplementary material
