# Supplementary material for: Recombination analysis of Human mastadenovirus C whole genomes
Source: Sci Rep. 2019 Feb 18;9:2182. doi: 10.1038/s41598-019-38719-z (PMC6379361; doi:10.1038/s41598-019-38719-z)
Supplement: Supplementary file 1 — Supplementary material [file 41598_2019_38719_MOESM1_ESM.pdf]

Recombination analysis of human *mastadenovirus* C whole genomes

Pierre Rivailler, Naiying Mao, Zhen Zhu and Wenbo Xu*

WHO WPRO Regional Reference Measles/Rubella Laboratory and Key Laboratory of Medical Virology and Viral Diseases, Ministry of Health of the People's Republic of China, National Institute for Viral Disease Control and Prevention, Chinese Center for Disease Control and Prevention, No.155, Changbai Road, Changping District, Beijing 102206, People's Republic of China

*Correspondence and requests for materials should be addressed to W.X (email: wenbo_xu1@aliyun.com).

Supplementary Table S1: lowest pairwise p distances for each genomic region and each analyzed sequences

Year	GB ID	WGS	p dist. [1,2,3]	1_7000	p dist. [5]	7000_14150	p dist.	Penton	p dist.	15867_18837	p dist.	Hexon	p dist.	21745_26000	p dist.	26001_31029	p dist.	Fiber	p dist.	32779_end	p dist.
1987	LC068713.1	**FJ349096.1-1953**	**0.005**	FJ349096.1-1953	0.002	FJ349096.1-1953	0.001	FJ349096.1-1953	0.001	FJ349096.1-1953	0.001	FJ349096.1-1953	0.001	FJ349096.1-1953	0.014	NC_001405-1953	0.007	FJ349096.1-1953	0.004	NC_001405-1953	0.015
																FJ349096.1-1953	0.007				
1992	KF268310.1	**NC_001405-1953**	**0.002**	NC_001405-1953	0.002	FJ349096.1-1953	0.003	NC_001405-1953	0.001	NC_001405-1953	0.001	NC_001405-1953	0	NC_001405-1953	0.001	NC_001405-1953	0.001	NC_001405-1953	0.001	NC_001405-1953	0.001
				FJ349096.1-1953	0.002	LC068713.1-1987	0.003														
				LC068713.1-1987	0.002	NC_001405-1953	0.004														
1993	LC068714.1	**LC068713.1-1987**	**0.002**	LC068713.1-1987	0.001	LC068713.1-1987	0.001	LC068713.1-1987	0.001	FJ349096.1-1953	0.001	FJ349096.1-1953	0.002	LC068713.1-1987	0.001	LC068713.1-1987	0	LC068713.1-1987	0	LC068713.1-1987	0.011
										LC068713.1-1987	0.001	LC068713.1-1987	0.002								
1994	LC068715.1	**LC068714.1-1993**	**0.001**	LC068714.1-1993	0.000	LC068713.1-1987	0.001	FJ349096.1-1953	0.001	FJ349096.1-1953	0.001	LC068713.1-1987	0	LC068714.1-1993	0.000	LC068713.1-1987	0.001	LC068713.1-1987	0.000	LC068714.1-1993	0.001
						LC068714.1-1993	0.001	LC068713.1-1987	0.001	LC068713.1-1987	0.001	LC068714.1-1993	0.002			LC068714.1-1993	0.001	LC068714.1-1993	0.000		
								LC068714.1-1993	0.001	LC068714.1-1993	0.001										
2000	JX173078.1	**AF534906.1-1953**	**0.003**	AF534906.1-1953	0.009	AF534906.1-1953	0.005	AF534906.1-1953	0.002	AF534906.1-1953	0.000	AF534906.1-1953	0.000	AF534906.1-1953	0.002	AF534906.1-1953	0.000	AF534906.1-1953	0.001	AF534906.1-1953	0.002
						KF268310.1-1992	0.005													NC_001405-1953	0.002
2001	JX173080.1	AF534906.1-1953	0.019	AF534906.1-1953	0.007	KF268310.1-1992	0.005	AF534906.1-1953	0.003	AF534906.1-1953	0.003	AF534906.1-1953	0.002	AF534906.1-1953	0.006	NC_001405-1953	0.017	JX173078.1-2000	0.009	LC068714.1-1993	0.012
		JX173078.1-2000	0.019			JX173078.1-2000	0.005	JX173078.1-2000	0.005	JX173078.1-2000	0.003	JX173078.1-2000	0.002			KF268310.1-1992	0.017			LC068715.1-1994	0.012
	JX173081.1	KF268310.1-1992	0.014	JX173080.1-2001	0.002	JX173080.1-2001	0.013	FJ349096.1-1953	0.026	FJ349096.1-1953	0.012	NC_001405-1953	0.022	NC_001405-1953	0.014	NC_001405-1953	0.012	NC_001405-1953	0.004	AC_000008.1-1953	0.010
								LC068713.1-1987	0.026	LC068713.1-1987	0.012	KF268310.1-1992	0.022	KF268310.1-1992	0.014	KF268310.1-1992	0.012	KF268310.1-1992	0.005		
										LC068714.1-1993	0.012										
										LC068715.1-1994	0.012										
	HQ003817.1	LC068713.1-1987	0.017	AF534906.1-1953	0.006	JX173078.1-2000	0.004	AF534906.1-1953	0.001	FJ349096.1-1953	0.007	LC068713.1-1987	0.107	LC068713.1-1987	0.010	FJ349096.1-1953	0.013	LC068713.1-1987	0.001	NC_001405-1953	0.009
		LC068714.1-1993	0.017					JX173078.1-2000	0.002	LC068713.1-1987	0.007	LC068714.1-1993	0.107	LC068714.1-1993	0.010			LC068714.1-1993	0.001	KF268310.1-1992	0.009
		LC068715.1-1994	0.017							LC068714.1-1993	0.007			LC068715.1-1994	0.010			LC068715.1-1994	0.001		
										LC068715.1-1994	0.007										
2002	JX173079.1	NC_001405-1953	0.009	HQ003817.1-2001	0.005	NC_001405-1953	0.004	LC068713.1-1987	0.013	AF534906.1-1953	0.011	NC_001405-1953	0.021	NC_001405-1953	0.013	NC_001405-1953	0.004	NC_001405-1953	0.003	NC_001405-1953	0.011
		KF268310.1-1992	0.009			KF268310.1-1992	0.004			FJ349096.1-1953	0.011	KF268310.1-1992	0.021	KF268310.1-1992	0.013	KF268310.1-1992	0.004	KF268310.1-1992	0.005	KF268310.1-1992	0.011
										LC068713.1-1987	0.011										
										LC068714.1-1993	0.011										
										LC068715.1-1994	0.011										
										JX173078.1-2000	0.011										
	KX384959.1	**JX173079.1-2002**	**0.003**	NC_001405-1953	0.003	KF268310.1-1992	0.005	JX173079.1-2002	0	JX173079.1-2002	0.003	JX173079.1-2002	0	JX173079.1-2002	0.001	JX173079.1-2002	0.001	JX173079.1-2002	0.001	JX173079.1-2002	0.002
				FJ349096.1-1953	0.003	JX173079.1-2002	0.006 [6]														
				LC068713.1-1987	0.003																
				KF268310.1-1992	0.003																
				LC068714.1-1993	0.003																
				LC068715.1-1994	0.003																
				JX173079.1-2002	0.007																
2003	JX173082.1	**AF534906.1-1953**	**0.002**	AF534906.1-1953	0.006	AF534906.1-1953	0.002	AF534906.1-1953	0.001	AF534906.1-1953	0.000	AF534906.1-1953	0.001	AF534906.1-1953	0.001	AF534906.1-1953	0.000	AF534906.1-1953	0.001	AF534906.1-1953	0.001
										JX173078.1-2000	0.000	JX173078.1-2000	0.001					JX173078.1-2000	0.001	JX173078.1-2000	0.001
	JX173083.1	**JX173082.1-2003**	**0.004**	JX173082.1-2003	0.005	KF268310.1-1992	0.003	AF534906.1-1953	0.001	AF534906.1-1953	0.002	AF534906.1-1953	0.002	AF534906.1-1953	0.003	AF534906.1-1953	0.003	JX173078.1-2000	0.007	NC_001405-1953	0.006
						JX173079.1-2002	0.003	HQ003817.1-2001	0.001	JX173078.1-2000	0.002	JX173078.1-2000	0.002	JX173082.1-2003	0.003	JX173078.1-2000	0.003	JX173080.1-2001	0.007	AC_000008.1-1953	0.006
						JX173082.1-2003	0.006	JX173082.1-2003	0.002	JX173082.1-2003	0.002	JX173082.1-2003	0.003			JX173082.1-2003	0.003	JX173082.1-2003	0.007	KF268310.1-1992	0.006
	JX173085.1	**JX173083.1-2003**	**0.001**	JX173082.1-2003	0.003	JX173083.1-2003	0	JX173083.1-2003	0.001	JX173083.1-2003	0	JX173083.1-2003	0	JX173083.1-2003	0	JX173083.1-2003	0	JX173083.1-2003	0.001	JX173083.1-2003	0
				JX173083.1-2003	0.003																
	JX173084.1	NC_001405-1953	0.004	AC_000008.1-1953	0.009	KF268310.1-1992	0.005	AF534906.1-1953	0.005	NC_001405-1953	0.001	NC_001405-1953	0	NC_001405-1953	0.001	NC_001405-1953	0.001	KF268310.1-1992	0	NC_001405-1953	0.001
		**KF268310.1-1992**	**0.004**	FJ349096.1-1953	0.009	JX173083.1-2003	0.005	KF268310.1-1992	0.005	KF268310.1-1992	0.001	KF268310.1-1992	0.001	KF268310.1-1992	0.001	KF268310.1-1992	0.001			KF268310.1-1992	0.001
								HQ003817.1-2001	0.005												
								JX173082.1-2003	0.005												
								JX173083.1-2003	0.005												
								JX173085.1-2003	0.005												
	LC068716.1	LC068713.1-1987	0.002	KF268310.1-1992	0.001	FJ349096.1-1953	0.005	LC068713.1-1987	0.000	FJ349096.1-1953	0.001	LC068713.1-1987	0	LC068714.1-1993	0	LC068714.1-1993	0	LC068713.1-1987	0	LC068713.1-1987	0.010
		LC068714.1-1993	0.002	LC068714.1-1993	0.001	LC068713.1-1987	0.005	LC068715.1-1994	0.001	LC068713.1-1987	0.001	LC068715.1-1994	0	LC068715.1-1994	0	LC068715.1-1994	0.001	LC068714.1-1993	0	LC068715.1-1994	0.010
		**LC068715.1-1994**	**0.002**	LC068715.1-1994	0.001	LC068714.1-1993	0.005	LC068714.1-1993	0.001	LC068714.1-1993	0.001	LC068714.1-1993	0.002					LC068715.1-1994	0		
						LC068715.1-1994	0.005			LC068715.1-1994	0.001										
2004	JX173086.1	**JX173085.1-2003**	**0.000**	JX173085.1-2003	0	JX173083.1-2003	0.000	JX173083.1-2003	0.001	JX173083.1-2003	0.000	JX173085.1-2003	0.000	JX173083.1-2003	0.000	JX173083.1-2003	0.000	JX173083.1-2003	0.001	JX173083.1-2003	0.001
						JX173085.1-2003	0.000	JX173085.1-2003	0.002	JX173085.1-2003	0.000			JX173085.1-2003	0.000	JX173085.1-2003	0.000	JX173085.1-2003	0.001	JX173085.1-2003	0.001
	LC068717.1	LC068713.1-1987	0.002	FJ349096.1-1953	0.001	FJ349096.1-1953	0.001	LC068713.1-1987	0.004	FJ349096.1-1953	0.001	LC068713.1-1987	0.000	LC068713.1-1987	0.000	LC068713.1-1987	0.001	LC068713.1-1987	0.002	LC068715.1-1994	0.006
		LC068714.1-1993	0.002	LC068713.1-1987	0.001	LC068713.1-1987	0.001	LC068716.1-2003	0.004	LC068713.1-1987	0.001	LC068715.1-1994	0.000	LC068715.1-1994	0.001	LC068714.1-1993	0.001	LC068714.1-1993	0.002	HQ003817.1-2001	0.006
		LC068715.1-1994	0.002	KF268310.1-1992	0.001	LC068714.1-1993	0.001	LC068715.1-1994	0.005	LC068715.1-1994	0.001	LC068716.1-2003	0.000	LC068714.1-1993	0.001	LC068715.1-1994	0.001	LC068715.1-1994	0.002		
		**LC068716.1-2003**	**0.003**	LC068714.1-1993	0.001	LC068715.1-1994	0.001	LC068714.1-1993	0.005	LC068716.1-2003	0.001	LC068714.1-1993	0.002	LC068716.1-2003	0.001	LC068716.1-2003	0.001	LC068716.1-2003	0.002		
				LC068715.1-1994	0.001					LC068714.1-1993	0.002										
				LC068716.1-2003	0.001																
	LC068718.1	LC068713.1-1987	0.002	LC068714.1-1993	0.000	LC068713.1-1987	0.001	AF534906.1-1953	0.001	FJ349096.1-1953	0.004	LC068713.1-1987	0.000	LC068714.1-1993	0	LC068714.1-1993	0	LC068713.1-1987	0.000	LC068713.1-1987	0.004
		LC068714.1-1993	0.002	LC068713.1-1987	0.001	LC068714.1-1993	0.001	HQ003817.1-2001	0.001	LC068713.1-1987	0.004	LC068715.1-1994	0.000	LC068715.1-1994	0	LC068713.1-1987	0.001	LC068714.1-1993	0.000		
		LC068715.1-1994	0.002	LC068717.1-2004	0.001	LC068715.1-1994	0.001	JX173083.1-2003	0.001	LC068715.1-1994	0.004	LC068716.1-2003	0.000	LC068716.1-2003	0	LC068717.1-2004	0.001	LC068715.1-1994	0.000		
		LC068716.1-2003	0.002			LC068717.1-2004	0.001	JX173085.1-2003	0.002	LC068716.1-2003	0.004	LC068717.1-2004	0.000	LC068713.1-1987	0.001			LC068716.1-2003	0.000		
		**LC068717.1-2004**	**0.002**					LC068717.1-2004	0.005	LC068717.1-2004	0.004			LC068717.1-2004	0.001			LC068717.1-2004	0.002		
	KF268130.1	**KF268310.1-1992**	**0.001**	FJ349096.1-1953	0.001	KF268310.1-1992	0.002	KF268310.1-1992	0	KF268310.1-1992	0.001	NC_001405-1953	0.001	NC_001405-1953	0.001	NC_001405-1953	0.001	NC_001405-1953	0.001	JX173084.1-2003	0.000
				KF268310.1-1992	0.001					JX173084.1-2003	0.001	KF268310.1-1992	0.001	KF268310.1-1992	0.001	KF268310.1-1992	0.001	KF268310.1-1992	0.002	KF268310.1-1992	0.002
				LC068717.1-2004	0.001							JX173084.1-2003	0.001	JX173084.1-2003	0.001						
				LC068718.1-2004	0.001																
2005	JX173077.1	KF268310.1-1992	0.000	KF268310.1-1992	0.001	KF268310.1-1992	0.000	KF268310.1-1992	0.000	NC_001405-1953	0.001	NC_001405-1953	0.000	NC_001405-1953	0.000	KF268310.1-1992	0.000	KF268310.1-1992	0.000	KF268310.1-1992	0.001
		**KF268130.1-2004**	**0.001**	LC068717.1-2004	0.001	KF268130.1-2004	0.002	KF268130.1-2004	0.000	KF268310.1-1992	0.001	KF268310.1-1992	0.000	JX173084.1-2003	0.000	KF268130.1-2004	0.001	JX173084.1-2003	0.000	KF268130.1-2004	0.002
				KF268130.1-2004	0.002					JX173084.1-2003	0.001	JX173084.1-2003	0.000	KF268310.1-1992	0.001			KF268130.1-2004	0.002		
										KF268130.1-2004	0.001	KF268130.1-2004	0.001	KF268130.1-2004	0.001						
	LC068720.1	**LC068717.1-2004**	**0.001**	LC068717.1-2004	0	LC068717.1-2004	0.002	LC068717.1-2004	0.002	LC068717.1-2004	0	LC068713.1-1987	0.000	LC068713.1-1987	0.000	LC068717.1-2004	0.000	LC068713.1-1987	0.001	LC068717.1-2004	0.000
												LC068715.1-1994	0.000	LC068717.1-2004	0.000			LC068714.1-1993	0.001		
												LC068716.1-2003	0.000					LC068715.1-1994	0.001		
												LC068717.1-2004	0.000					LC068716.1-2003	0.001		
												LC068718.1-2004	0.000					LC068717.1-2004	0.001		
																		LC068718.1-2004	0.001		
	KF268129.1	LC068713.1-1987	0.026	JX173078.1-2000	0.012	KF268310.1-1992	0.018	AF534906.1-1953	0.012	FJ349096.1-1953	0.014	FJ349096.1-1953	0.017	AC_000008.1-1953	0.024	JX173079.1-2002	0.081	FJ349096.1-1953	0.018	LC068716.1-2003	0.008
		LC068714.1-1993	0.026			JX173083.1-2003	0.018	FJ349096.1-1953	0.012	LC068713.1-1987	0.014	LC068713.1-1987	0.017								
		LC068715.1-1994	0.026			JX173085.1-2003	0.018	LC068713.1-1987	0.012	LC068714.1-1993	0.014	LC068714.1-1993	0.017								
		LC068716.1-2003	0.026			JX173086.1-2004	0.018	LC068715.1-1994	0.012	LC068715.1-1994	0.014	LC068715.1-1994	0.017								
		LC068718.1-2004	0.026			JX173077.1-2005	0.018	HQ003817.1-2001	0.012	LC068717.1-2004	0.014	LC068716.1-2003	0.017								
		LC068720.1-2005	0.026					LC068716.1-2003	0.012	LC068720.1-2005	0.014	LC068717.1-2004	0.017								
								JX173083.1-2003	0.012			LC068718.1-2004	0.017								
								LC068718.1-2004	0.012			LC068720.1-2005	0.017								
2007	JX423389.1	LC068717.1-2004	0.003	JX173078.1-2000	0.007	LC068713.1-1987	0.004	LC068713.1-1987	0.000	FJ349096.1-1953	0.001	LC068713.1-1987	0.000	LC068713.1-1987	0.001	LC068713.1-1987	0	LC068713.1-1987	0.001	LC068717.1-2004	0.001
		**LC068720.1-2005**	**0.004**			LC068717.1-2004	0.005	LC068716.1-2003	0.000	LC068713.1-1987	0.001	LC068715.1-1994	0.000	LC068714.1-1993	0.001	LC068714.1-1993	0	LC068714.1-1993	0.001	LC068720.1-2005	0.001
						LC068720.1-2005	0.007 [6]	LC068717.1-2004	0.004	LC068714.1-1993	0.001	LC068716.1-2003	0.000	LC068715.1-1994	0.001	LC068716.1-2003	0	LC068715.1-1994	0.001		
								LC068720.1-2005	0.005	LC068715.1-1994	0.001	LC068717.1-2004	0.000	LC068716.1-2003	0.001	LC068718.1-2004	0	LC068716.1-2003	0.001		
										LC068716.1-2003	0.001	LC068718.1-2004	0.000	LC068717.1-2004	0.001	LC068717.1-2004	0.001	LC068718.1-2004	0.001		
										LC068717.1-2004	0.001	LC068720.1-2005	0.000	LC068718.1-2004	0.001	LC068720.1-2005	0.001	LC068720.1-2005	0.001		
										LC068720.1-2005	0.001			LC068720.1-2005	0.001			LC068717.1-2004	0.003		
2008	KF268199.1	AC_000008.1-1953	0.008	AC_000008.1-1953	0.009	JX173083.1-2003	0.007	AF534906.1-1953	0.005 [7]	LC068718.1-2004	0.003	AC_000008.1-1953	0.008 [8]	AC_000008.1-1953	0.003	AC_000008.1-1953	0.006	AC_000008.1-1953	0.002	JX173086.1-2004	0.001
								FJ349096.1-1953	0.005												
								LC068713.1-1987	0.005												
								LC068715.1-1994	0.005												
								HQ003817.1-2001	0.005												
								LC068716.1-2003	0.005												
								JX173083.1-2003	0.005												
								LC068718.1-2004	0.005												
								JX423389.1-2007	0.005												
2009	KR699642.1	KF268310.1-1992	0.007	KF268310.1-1992	0.002	KF268310.1-1992	0.003	AF534906.1-1953	0.002	JX173078.1-2000	0.002	NC_001405-1953	0.012	JX173083.1-2003	0.012	NC_001405-1953	0.004	NC_001405-1953	0.003	JX173083.1-2003	0.004
		**KF268130.1-2004**	**0.007 [4]**	LC068717.1-2004	0.002	KF268130.1-2004	0.004	HQ003817.1-2001	0.002	JX173086.1-2004	0.003	KF268310.1-1992	0.012	JX173085.1-2003	0.012	KF268310.1-1992	0.004	KF268130.1-2004	0.003	JX173085.1-2003	0.004
		JX173077.1-2005	0.007	LC068720.1-2005	0.002			JX173083.1-2003	0.002			JX173084.1-2003	0.012	JX173086.1-2004	0.012	JX173084.1-2003	0.004			JX173086.1-2004	0.005
				KF268130.1-2004	0.003			LC068718.1-2004	0.002			KF268130.1-2004	0.012			KF268130.1-2004	0.004				
												JX173077.1-2005	0.012			JX173077.1-2005	0.004				
2012	MF315028	NC_001405-1953	0.006	JX173078.1-2000	0.004	JX173083.1-2003	0.003	AF534906.1-1953	0.002	LC068718.1-2004	0.006	NC_001405-1953	0.001	NC_001405-1953	0.001	NC_001405-1953	0.002	KF268130.1-2004	0.001	LC068716.1-2003	0.010
		KF268310.1-1992	0.006	JX173086.1-2004	0.004	JX173085.1-2003	0.003	HQ003817.1-2001	0.002			KF268310.1-1992	0.001	JX173084.1-2003	0.001	KF268310.1-1992	0.002	JX173077.1-2005	0.002	LC068718.1-2004	0.010
		KF268130.1-2004	0.006			JX173086.1-2004	0.003	JX173083.1-2003	0.002			JX173084.1-2003	0.001	JX173077.1-2005	0.001	JX173077.1-2005	0.002				
		**JX173077.1-2005**	**0.006 [4]**			JX173077.1-2005	0.005	LC068718.1-2004	0.002			KF268130.1-2004	0.001								
												JX173077.1-2005	0.001								
2013	KF951595.1	LC068717.1-2004	0.001	LC068713.1-1987	0.001	FJ349096.1-1953	0.001	LC068713.1-1987	0.001	FJ349096.1-1953	0.002	LC068713.1-1987	0.000	LC068713.1-1987	0	LC068714.1-1993	0.000	LC068713.1-1987	0	LC068717.1-2004	0.002
		**LC068720.1-2005**	**0.002**	KF268310.1-1992	0.001	LC068713.1-1987	0.001	LC068716.1-2003	0.001	LC068713.1-1987	0.002	LC068715.1-1994	0.000	LC068717.1-2004	0	JX423389.1-2007	0.000	LC068714.1-1993	0	LC068720.1-2005	0.002
				LC068714.1-1993	0.001	LC068714.1-1993	0.001	JX423389.1-2007	0.001	LC068714.1-1993	0.002	LC068716.1-2003	0.000	LC068720.1-2005	0	LC068717.1-2004	0.001	LC068715.1-1994	0		
				LC068715.1-1994	0.001	LC068715.1-1994	0.001	LC068717.1-2004	0.005	LC068715.1-1994	0.002	LC068717.1-2004	0.000			LC068720.1-2005	0.001	LC068716.1-2003	0		
				LC068716.1-2003	0.001	LC068717.1-2004	0.001	LC068720.1-2005	0.005	LC068716.1-2003	0.002	LC068718.1-2004	0.000					LC068718.1-2004	0		
				KF268130.1-2004	0.001	LC068718.1-2004	0.001			LC068717.1-2004	0.002	LC068720.1-2005	0.000					LC068717.1-2004	0.002		
				LC068717.1-2004	0.001	LC068720.1-2005	0.003			LC068720.1-2005	0.002	JX423389.1-2007	0.000					LC068720.1-2005	0.001		
				LC068718.1-2004	0.001					JX423389.1-2007	0.002										
				LC068720.1-2005	0.001																
	MF315029	**KR699642.1-2009**	**0.004**	KF268199.1	0.006 [8]	JX173083.1-2003	0.001	AF534906.1-1953	0.002	JX173083.1-2003	0.002	KR699642.1-2009	0.000	KR699642.1-2009	0.002	KR699642.1-2009	0.001	KR699642.1-2009	0.001	KR699642.1-2009	0.001
						JX173085.1-2003	0.002	HQ003817.1-2001	0.002	JX173085.1-2003	0.002							MF315028-2012	0.001		
						JX173086.1-2004	0.002	JX173082.1-2003	0.002	KR699642.1-2009	0.005										
						KR699642.1-2009	0.005	JX173083.1-2003	0.002												
								JX173085.1-2003	0.002												
								LC068718.1-2004	0.002												
								KR699642.1-2009	0.003												

1: p-distances were selected from pairwise p-distance tables shown in supplementary tables S2-S11.
2: p-distances (p dist.) > 0.005 are considered divergent and are highlighted in dark grey.
3: WGS sequences with p-distance ≤0.005 are considered as the backbone of the recombinant virus and are indicated in bold face.
4: KF268130 and JX173077 were considered as backbone based on p-distances <0.005 across the genome.
5: sequences with a p-distance ≤0.005 were highlighted in light grey and considered involved in recombination after checking the corresponding phylogenetic tree.
6: the p-distance was not considered divergent based on WGS p-distance.
7: the p-distance was considered divergent based on the phylogenetic tree (bootstrap value).
8: the p-distance was not considered divergent based on the phylogenetic tree.

Supplementary table S2: pairwise p-distance for 32 WGS

			1 [1]	2	3	4	5	6	7	8	9	10	11	12	13	14	15	16	17	18	19	20	21	22	23	24	25	26	27	28	29	30	31
#	Year	GB ID	1953	1953	1953	1953	1987	1992	1993	1994	2000	2001	2001	2001	2002	2002	2003	2003	2003	2003	2003	2004	2004	2004	2004	2005	2005	2005	2007	2008	2009	2012	2013
1	1953	AF534906.1																															
2	1953	NC_001405.1	0.046																														
3	1953	AC_000008.1	0.050	0.050																													
4	1953	FJ349096.1	0.044	0.024	0.049																												
5	1987	LC068713.1	0.045	0.027	0.050	0.005																											
6	1992	KF268310.1	0.046	0.002	0.049	0.024	0.026																										
7	1993	LC068714.1	0.045	0.027	0.050	0.005	0.002	0.026																									
8	1994	LC068715.1	0.045	0.027	0.050	0.005	0.002	0.026	0.001																								
9	2000	JX173078.1	0.003	0.045	0.050	0.043	0.044	0.045	0.045	0.045																							
10	2001	JX173080.1	0.019	0.036	0.051	0.033	0.034	0.035	0.034	0.034	0.019																						
11	2001	JX173081.1	0.049	0.015	0.054	0.033	0.034	0.014	0.034	0.034	0.049	0.036																					
12	2001	HQ003817.1	0.041	0.031	0.051	0.018	0.017	0.031	0.017	0.017	0.041	0.030	0.035																				
13	2002	JX173079.1	0.046	0.009	0.051	0.027	0.028	0.009	0.028	0.028	0.045	0.035	0.015	0.032																			
14	2002	KX384959.1	0.047	0.009	0.051	0.026	0.027	0.008	0.027	0.027	0.046	0.036	0.016	0.033	0.003																		
15	2003	JX173082.1	0.002	0.046	0.050	0.044	0.045	0.046	0.045	0.046	0.003	0.020	0.049	0.041	0.046	0.047																	
16	2003	JX173083.1	0.005	0.045	0.049	0.042	0.044	0.045	0.044	0.044	0.005	0.018	0.047	0.041	0.045	0.045	0.004																
17	2003	JX173085.1	0.005	0.045	0.049	0.043	0.044	0.045	0.044	0.044	0.005	0.019	0.048	0.041	0.045	0.046	0.004	0.001															
18	2003	JX173084.1	0.046	0.004	0.050	0.026	0.028	0.004	0.028	0.028	0.046	0.037	0.016	0.032	0.010	0.011	0.046	0.046	0.046														
19	2003	LC068716.1	0.046	0.027	0.050	0.006	0.002	0.026	0.002	0.002	0.045	0.034	0.034	0.018	0.028	0.028	0.046	0.044	0.045	0.029													
20	2004	JX173086.1	0.005	0.045	0.049	0.043	0.044	0.045	0.044	0.044	0.005	0.018	0.048	0.041	0.045	0.046	0.004	0.001	0.000	0.046	0.045												
21	2004	LC068717.1	0.045	0.026	0.050	0.005	0.002	0.026	0.002	0.002	0.044	0.033	0.034	0.017	0.027	0.027	0.045	0.043	0.044	0.028	0.003	0.044											
22	2004	LC068718.1	0.045	0.026	0.050	0.005	0.002	0.026	0.002	0.002	0.044	0.033	0.033	0.017	0.028	0.027	0.045	0.043	0.044	0.028	0.002	0.044	0.002										
23	2004	KF268130.1	0.046	0.002	0.050	0.023	0.026	0.001	0.026	0.026	0.045	0.036	0.015	0.031	0.009	0.009	0.046	0.045	0.045	0.004	0.027	0.045	0.025	0.026									
24	2005	JX173077.1	0.046	0.002	0.049	0.024	0.026	0.000	0.026	0.026	0.045	0.035	0.014	0.031	0.009	0.009	0.046	0.044	0.045	0.004	0.026	0.045	0.025	0.026	0.001								
25	2005	KF268129.1	0.050	0.044	0.054	0.027	0.026	0.044	0.026	0.026	0.049	0.048	0.047	0.035	0.044	0.044	0.050	0.049	0.049	0.044	0.026	0.049	0.027	0.026	0.044	0.043							
26	2005	LC068720.1	0.045	0.026	0.050	0.005	0.002	0.025	0.002	0.002	0.044	0.033	0.034	0.017	0.027	0.027	0.045	0.043	0.044	0.028	0.003	0.044	0.001	0.003	0.025	0.025	0.026						
27	2007	JX423389.1	0.045	0.028	0.050	0.007	0.004	0.028	0.004	0.004	0.044	0.033	0.034	0.017	0.028	0.029	0.045	0.044	0.044	0.029	0.005	0.044	0.003	0.004	0.028	0.028	0.027	0.004					
28	2008	KF268199.1	0.048	0.051	0.008	0.049	0.050	0.050	0.050	0.050	0.048	0.050	0.054	0.050	0.051	0.052	0.049	0.047	0.047	0.050	0.050	0.047	0.050	0.050	0.051	0.050	0.054	0.050	0.050				
29	2009	KR699642.1	0.044	0.008	0.051	0.026	0.027	0.007	0.027	0.027	0.043	0.034	0.015	0.031	0.010	0.010	0.044	0.042	0.043	0.009	0.027	0.043	0.027	0.026	0.007	0.007	0.044	0.027	0.028	0.051			
30	2012	MF315028	0.046	0.006	0.051	0.026	0.028	0.006	0.028	0.028	0.045	0.035	0.015	0.031	0.010	0.011	0.046	0.044	0.044	0.007	0.028	0.044	0.027	0.027	0.006	0.006	0.043	0.027	0.028	0.050	0.009		
31	2013	KF951595.1	0.045	0.026	0.050	0.005	0.002	0.026	0.002	0.002	0.044	0.033	0.034	0.017	0.028	0.027	0.045	0.044	0.044	0.028	0.003	0.044	0.001	0.002	0.026	0.026	0.027	0.002	0.003	0.050	0.027	0.028	
32	2013	MF315029	0.044	0.009	0.051	0.028	0.028	0.009	0.029	0.029	0.044	0.034	0.016	0.032	0.010	0.011	0.044	0.042	0.042	0.009	0.029	0.042	0.028	0.028	0.009	0.009	0.045	0.028	0.029	0.049	0.004	0.008	0.029

1: the lowest p-distance is highlighted for each sequence of viruses collected after 1953 and is reported in Supplementary Table S1.

Supplementary Table S3: pairwise p-distance for the genomic region 1-7000 of the 32 analyzed genomes

			1 [1]	2	3	4	5	6	7	8	9	10	11	12	13	14	15	16	17	18	19	20	21	22	23	24	25	26	27	28	29	30	31
#	Year	GB ID	1953	1953	1953	1953	1987	1992	1993	1994	2000	2001	2001	2001	2002	2002	2003	2003	2003	2003	2003	2004	2004	2004	2004	2005	2005	2005	2007	2008	2009	2012	2013
1	1953	AF534906.1																															
2	1953	NC_001405.1	0.013																														
3	1953	AC_000008.1	0.013	0.008																													
4	1953	FJ349096.1	0.012	0.002	0.008																												
5	1987	LC068713.1	0.013	0.003	0.008	0.002																											
6	1992	KF268310.1	0.012	0.002	0.008	0.002	0.002																										
7	1993	LC068714.1	0.013	0.002	0.008	0.002	0.001	0.002																									
8	1994	LC068715.1	0.013	0.002	0.008	0.002	0.002	0.002	0.000																								
9	2000	JX173078.1	0.009	0.010	0.011	0.010	0.010	0.010	0.010	0.010																							
10	2001	JX173080.1	0.007	0.012	0.011	0.012	0.012	0.011	0.012	0.012	0.010																						
11	2001	JX173081.1	0.007	0.012	0.012	0.012	0.012	0.012	0.012	0.012	0.010	0.002																					
12	1997	HQ003817.1	0.006	0.009	0.012	0.009	0.009	0.009	0.009	0.009	0.007	0.009	0.009																				
13	2002	JX173079.1	0.007	0.008	0.011	0.007	0.008	0.007	0.008	0.008	0.007	0.010	0.010	0.005																			
14	2002	KX384959.1	0.012	0.003	0.007	0.003	0.003	0.003	0.003	0.003	0.010	0.010	0.011	0.010	0.007																		
15	2003	JX173082.1	0.006	0.012	0.012	0.012	0.013	0.012	0.012	0.013	0.009	0.009	0.009	0.008	0.009	0.012																	
16	2003	JX173083.1	0.007	0.009	0.010	0.009	0.010	0.009	0.009	0.010	0.009	0.007	0.007	0.009	0.010	0.009	0.005																
17	2003	JX173085.1	0.006	0.011	0.011	0.011	0.011	0.011	0.011	0.011	0.008	0.008	0.008	0.008	0.008	0.010	0.003	0.003															
18	2003	JX173084.1	0.014	0.010	0.009	0.009	0.010	0.010	0.010	0.010	0.011	0.014	0.015	0.012	0.011	0.010	0.013	0.013	0.012														
19	2003	LC068716.1	0.013	0.002	0.008	0.002	0.002	0.001	0.001	0.001	0.010	0.012	0.012	0.009	0.008	0.003	0.013	0.010	0.011	0.010													
20	2004	JX173086.1	0.006	0.011	0.011	0.011	0.011	0.011	0.011	0.011	0.008	0.008	0.008	0.007	0.008	0.010	0.003	0.002	0.000	0.012	0.011												
21	2004	LC068717.1	0.012	0.002	0.008	0.001	0.001	0.001	0.001	0.001	0.010	0.011	0.011	0.008	0.007	0.003	0.012	0.009	0.010	0.009	0.001	0.010											
22	2004	LC068718.1	0.013	0.002	0.008	0.002	0.001	0.001	0.000	0.001	0.010	0.012	0.012	0.009	0.007	0.003	0.012	0.009	0.011	0.010	0.001	0.011	0.001										
23	2004	KF268130.1	0.012	0.002	0.008	0.001	0.002	0.001	0.002	0.002	0.010	0.011	0.012	0.009	0.007	0.003	0.012	0.009	0.011	0.009	0.002	0.011	0.001	0.001									
24	2005	JX173077.1	0.012	0.002	0.008	0.002	0.002	0.001	0.002	0.002	0.010	0.011	0.012	0.008	0.007	0.003	0.012	0.009	0.011	0.010	0.002	0.011	0.001	0.002	0.002								
25	2005	KF268129.1	0.014	0.013	0.015	0.013	0.013	0.013	0.013	0.013	0.012	0.013	0.014	0.013	0.014	0.013	0.015	0.013	0.014	0.016	0.013	0.013	0.013	0.013	0.013	0.013							
26	2005	LC068720.1	0.012	0.002	0.008	0.001	0.001	0.001	0.001	0.001	0.010	0.011	0.012	0.008	0.007	0.003	0.012	0.009	0.011	0.010	0.001	0.010	0.000	0.001	0.001	0.001	0.013						
27	2007	JX423389.1	0.009	0.009	0.009	0.009	0.009	0.009	0.009	0.009	0.007	0.009	0.010	0.010	0.009	0.008	0.010	0.008	0.009	0.012	0.009	0.009	0.009	0.009	0.008	0.009	0.012	0.009					
28	2008	KF268199.1	0.012	0.012	0.009	0.012	0.012	0.011	0.012	0.012	0.011	0.013	0.013	0.012	0.010	0.011	0.012	0.012	0.011	0.011	0.012	0.011	0.012	0.012	0.011	0.012	0.016	0.012	0.011				
29	2009	KR699642.1	0.011	0.003	0.007	0.003	0.003	0.002	0.003	0.003	0.010	0.010	0.010	0.009	0.007	0.003	0.011	0.008	0.010	0.010	0.003	0.010	0.002	0.003	0.003	0.003	0.013	0.002	0.008	0.011			
30	2012	MF315028	0.006	0.009	0.010	0.009	0.010	0.009	0.009	0.010	0.004	0.008	0.008	0.006	0.006	0.009	0.006	0.006	0.005	0.010	0.010	0.004	0.009	0.009	0.009	0.009	0.011	0.009	0.007	0.010	0.009		
31	2013	KF951595.1	0.013	0.002	0.008	0.002	0.001	0.001	0.001	0.001	0.010	0.012	0.012	0.009	0.007	0.003	0.012	0.009	0.011	0.010	0.001	0.011	0.001	0.001	0.001	0.002	0.013	0.001	0.009	0.012	0.003	0.009	
32	2013	MF315029	0.012	0.011	0.007	0.011	0.012	0.011	0.011	0.012	0.010	0.014	0.014	0.012	0.010	0.011	0.011	0.011	0.010	0.011	0.012	0.010	0.011	0.011	0.011	0.012	0.017	0.011	0.012	0.006	0.011	0.008	0.011

1: the lowest p-distance is highlighted for each sequence of viruses collected after 1953 and is reported in Supplementary Table S1.

Supplementary Table S4: pairwise p-distance for the genomic region 7001-14150 of the 32 analyzed genomes

			1 [1]	2	3	4	5	6	7	8	9	10	11	12	13	14	15	16	17	18	19	20	21	22	23	24	25	26	27	28	29	30	31
#	Year	GB ID	1953	1953	1953	1953	1987	1992	1993	1994	2000	2001	2001	2001	2002	2002	2003	2003	2003	2003	2003	2004	2004	2004	2004	2005	2005	2005	2007	2008	2009	2012	2013
1	1953	AF534906.1																															
2	1953	NC_001405.1	0.008																														
3	1953	AC_000008.1	0.013	0.011																													
4	1953	FJ349096.1	0.007	0.006	0.010																												
5	1987	LC068713.1	0.008	0.007	0.010	0.001																											
6	1992	KF268310.1	0.007	0.004	0.009	0.003	0.003																										
7	1993	LC068714.1	0.008	0.007	0.011	0.002	0.001	0.004																									
8	1994	LC068715.1	0.008	0.007	0.011	0.002	0.001	0.004	0.001																								
9	2000	JX173078.1	0.005	0.006	0.012	0.006	0.006	0.005	0.007	0.007																							
10	2001	JX173080.1	0.008	0.006	0.011	0.006	0.007	0.005	0.007	0.007	0.005																						
11	2001	JX173081.1	0.017	0.016	0.020	0.016	0.017	0.015	0.017	0.017	0.014	0.013																					
12	2001	HQ003817.1	0.005	0.007	0.012	0.006	0.007	0.006	0.007	0.007	0.004	0.007	0.016																				
13	2002	JX173079.1	0.007	0.004	0.011	0.006	0.006	0.004	0.006	0.006	0.005	0.006	0.016	0.007																			
14	2002	KX384959.1	0.009	0.006	0.010	0.006	0.007	0.005	0.007	0.007	0.007	0.007	0.016	0.007	0.006																		
15	2003	JX173082.1	0.002	0.008	0.013	0.008	0.008	0.007	0.009	0.009	0.005	0.008	0.017	0.006	0.008	0.009																	
16	2003	JX173083.1	0.006	0.004	0.009	0.004	0.005	0.003	0.005	0.005	0.004	0.004	0.014	0.005	0.003	0.004	0.006																
17	2003	JX173085.1	0.006	0.004	0.010	0.005	0.005	0.003	0.005	0.005	0.004	0.005	0.015	0.005	0.003	0.005	0.006	0.000															
18	2003	JX173084.1	0.008	0.007	0.010	0.007	0.008	0.005	0.008	0.008	0.008	0.008	0.017	0.009	0.007	0.008	0.009	0.005	0.006														
19	2003	LC068716.1	0.010	0.009	0.011	0.005	0.005	0.006	0.005	0.005	0.009	0.009	0.019	0.010	0.009	0.010	0.011	0.008	0.008	0.010													
20	2004	JX173086.1	0.006	0.004	0.010	0.005	0.005	0.003	0.005	0.005	0.004	0.005	0.015	0.005	0.003	0.005	0.006	0.000	0.000	0.006	0.008												
21	2004	LC068717.1	0.008	0.006	0.010	0.001	0.001	0.003	0.001	0.001	0.006	0.007	0.017	0.007	0.006	0.007	0.008	0.005	0.005	0.007	0.005	0.005											
22	2004	LC068718.1	0.008	0.006	0.010	0.002	0.001	0.003	0.001	0.001	0.006	0.007	0.017	0.007	0.006	0.007	0.008	0.005	0.005	0.007	0.005	0.005	0.001										
23	2004	KF268130.1	0.008	0.005	0.010	0.003	0.004	0.002	0.004	0.004	0.006	0.007	0.016	0.007	0.005	0.006	0.008	0.004	0.004	0.006	0.007	0.004	0.004	0.004									
24	2005	JX173077.1	0.007	0.004	0.009	0.003	0.004	0.000	0.004	0.004	0.005	0.006	0.016	0.006	0.004	0.005	0.007	0.003	0.003	0.005	0.007	0.003	0.004	0.004	0.002								
25	2005	KF268129.1	0.021	0.020	0.020	0.019	0.020	0.018	0.020	0.020	0.020	0.020	0.024	0.020	0.019	0.019	0.022	0.018	0.018	0.020	0.019	0.018	0.019	0.020	0.019	0.018							
26	2005	LC068720.1	0.008	0.006	0.010	0.003	0.003	0.003	0.004	0.004	0.006	0.007	0.016	0.007	0.006	0.006	0.008	0.004	0.005	0.007	0.007	0.005	0.002	0.003	0.003	0.003	0.019						
27	2007	JX423389.1	0.010	0.009	0.009	0.005	0.004	0.006	0.005	0.005	0.008	0.008	0.018	0.008	0.008	0.009	0.010	0.006	0.006	0.009	0.008	0.006	0.005	0.005	0.007	0.007	0.021	0.007					
28	2008	KF268199.1	0.011	0.009	0.010	0.009	0.009	0.008	0.010	0.010	0.009	0.008	0.017	0.010	0.008	0.008	0.011	0.007	0.008	0.011	0.011	0.008	0.009	0.009	0.009	0.009	0.018	0.010	0.010				
29	2009	KR699642.1	0.006	0.006	0.011	0.004	0.004	0.003	0.005	0.005	0.005	0.007	0.017	0.005	0.006	0.007	0.007	0.005	0.005	0.008	0.007	0.005	0.004	0.004	0.004	0.004	0.019	0.004	0.008	0.010			
30	2012	MF315028	0.008	0.006	0.012	0.007	0.007	0.005	0.007	0.007	0.006	0.007	0.017	0.007	0.005	0.007	0.008	0.003	0.003	0.008	0.010	0.003	0.007	0.007	0.006	0.005	0.020	0.007	0.008	0.010	0.007		
31	2013	KF951595.1	0.008	0.007	0.010	0.001	0.001	0.003	0.001	0.001	0.006	0.007	0.017	0.007	0.006	0.007	0.008	0.005	0.005	0.008	0.005	0.005	0.001	0.001	0.004	0.004	0.020	0.003	0.005	0.010	0.004	0.007	
32	2013	MF315029	0.006	0.005	0.010	0.005	0.005	0.003	0.006	0.006	0.005	0.005	0.015	0.005	0.003	0.005	0.006	0.001	0.002	0.006	0.008	0.002	0.005	0.005	0.004	0.004	0.019	0.005	0.006	0.008	0.005	0.003	0.005

1: the lowest p-distance is highlighted for each sequence of viruses collected after 1953 and is reported in Supplementary Table S1.

Supplementary Table S5: pairwise p-distance for the penton gene (nt 14151-15866) of the 32 analyzed genomes

			1[1]	2	3	4	5	6	7	8	9	10	11	12	13	14	15	16	17	18	19	20	21	22	23	24	25	26	27	28	29	30	31
#	Year	GB ID	1953	1953	1953	1953	1987	1992	1993	1994	2000	2001	2001	2001	2002	2002	2003	2003	2003	2003	2003	2004	2004	2004	2004	2005	2005	2005	2007	2008	2009	2012	2013
1	1953	AF534906.1																															
2	1953	NC_001405.1	0.008																														
3	1953	AC_000008.1	0.015	0.015																													
4	1953	FJ349096.1	0.003	0.009	0.013																												
5	1987	LC068713.1	0.003	0.008	0.013	0.001																											
6	1992	KF268310.1	0.006	0.001	0.013	0.008	0.007																										
7	1993	LC068714.1	0.004	0.009	0.015	0.002	0.001	0.008																									
8	1994	LC068715.1	0.003	0.009	0.014	0.001	0.001	0.008	0.001																								
9	2000	JX173078.1	0.002	0.009	0.016	0.005	0.004	0.008	0.005	0.005																							
10	2001	JX173080.1	0.003	0.009	0.016	0.006	0.005	0.008	0.006	0.006	0.005																						
11	2001	JX173081.1	0.027	0.029	0.028	0.026	0.026	0.028	0.028	0.027	0.028	0.028																					
12	1997	HQ003817.1	0.001	0.008	0.015	0.003	0.003	0.006	0.004	0.003	0.002	0.003	0.026																				
13	2002	JX173079.1	0.015	0.017	0.016	0.014	0.013	0.016	0.015	0.014	0.016	0.016	0.031	0.015																			
14	2002	KX384959.1	0.015	0.017	0.016	0.014	0.013	0.016	0.015	0.014	0.016	0.016	0.031	0.015	0.000																		
15	2003	JX173082.1	0.001	0.008	0.016	0.004	0.003	0.007	0.005	0.004	0.003	0.004	0.028	0.002	0.016	0.016																	
16	2003	JX173083.1	0.001	0.008	0.015	0.003	0.003	0.006	0.004	0.003	0.002	0.003	0.027	0.001	0.015	0.015	0.002																
17	2003	JX173085.1	0.002	0.008	0.016	0.004	0.003	0.007	0.005	0.004	0.003	0.004	0.026	0.002	0.016	0.016	0.002	0.001															
18	2003	JX173084.1	0.005	0.006	0.016	0.007	0.006	0.005	0.008	0.007	0.006	0.006	0.029	0.005	0.018	0.018	0.005	0.005	0.005														
19	2003	LC068716.1	0.003	0.008	0.013	0.001	0.000	0.007	0.001	0.001	0.004	0.005	0.026	0.003	0.013	0.013	0.003	0.003	0.003	0.006													
20	2004	JX173086.1	0.002	0.009	0.016	0.005	0.004	0.008	0.005	0.005	0.003	0.005	0.028	0.002	0.016	0.016	0.003	0.001	0.002	0.006	0.004												
21	2004	LC068717.1	0.005	0.009	0.016	0.005	0.004	0.008	0.005	0.005	0.006	0.007	0.029	0.005	0.016	0.016	0.005	0.005	0.005	0.008	0.004	0.006											
22	2004	LC068718.1	0.001	0.008	0.015	0.003	0.003	0.006	0.004	0.003	0.002	0.003	0.026	0.001	0.015	0.015	0.002	0.001	0.002	0.005	0.003	0.002	0.005										
23	2004	KF268130.1	0.006	0.001	0.013	0.008	0.007	0.000	0.008	0.008	0.008	0.008	0.028	0.006	0.016	0.016	0.007	0.006	0.007	0.005	0.007	0.008	0.008	0.006									
24	2005	JX173077.1	0.006	0.001	0.013	0.008	0.007	0.000	0.008	0.008	0.008	0.008	0.028	0.006	0.016	0.016	0.007	0.006	0.007	0.005	0.007	0.008	0.008	0.006	0.000								
25	2005	KF268129.1	0.012	0.016	0.013	0.012	0.012	0.015	0.013	0.012	0.013	0.013	0.024	0.012	0.018	0.018	0.013	0.012	0.013	0.015	0.012	0.013	0.015	0.012	0.015	0.015							
26	2005	LC068720.1	0.004	0.009	0.016	0.005	0.005	0.008	0.006	0.005	0.005	0.006	0.030	0.004	0.016	0.016	0.005	0.004	0.005	0.007	0.005	0.005	0.002	0.004	0.008	0.008	0.015						
27	2007	JX423389.1	0.003	0.008	0.013	0.001	0.000	0.007	0.001	0.001	0.004	0.005	0.026	0.003	0.013	0.013	0.003	0.003	0.003	0.006	0.000	0.004	0.004	0.003	0.007	0.007	0.012	0.005					
28	2008	KF268199.1	0.005	0.012	0.014	0.005	0.005	0.011	0.006	0.005	0.006	0.006	0.026	0.005	0.014	0.014	0.006	0.005	0.006	0.006	0.005	0.006	0.007	0.005	0.011	0.011	0.011	0.008	0.005				
29	2009	KR699642.1	0.002	0.008	0.016	0.005	0.004	0.006	0.005	0.005	0.003	0.005	0.028	0.002	0.015	0.015	0.003	0.002	0.003	0.005	0.004	0.003	0.006	0.002	0.006	0.006	0.013	0.005	0.004	0.006			
30	2012	MF315028	0.002	0.007	0.015	0.005	0.004	0.006	0.005	0.005	0.003	0.005	0.027	0.002	0.015	0.015	0.003	0.002	0.003	0.005	0.004	0.003	0.003	0.002	0.006	0.006	0.013	0.003	0.004	0.006	0.003		
31	2013	KF951595.1	0.004	0.009	0.015	0.002	0.001	0.008	0.002	0.002	0.005	0.006	0.028	0.004	0.015	0.015	0.005	0.004	0.005	0.008	0.001	0.005	0.005	0.004	0.008	0.008	0.013	0.006	0.001	0.006	0.005	0.005	
32	2013	MF315029	0.002	0.008	0.016	0.004	0.003	0.007	0.005	0.004	0.003	0.004	0.028	0.002	0.016	0.016	0.002	0.002	0.002	0.005	0.003	0.003	0.005	0.002	0.007	0.007	0.013	0.005	0.003	0.006	0.003	0.003	0.005

1: the lowest p-distance is highlighted for each sequence of viruses collected after 1953 and is reported in Supplementary Table S1.

Supplementary Table S6: pairwise p-distance for the genomic region 15867-18837 of the 32 analyzed genomes

			1[1]	2	3	4	5	6	7	8	9	10	11	12	13	14	15	16	17	18	19	20	21	22	23	24	25	26	27	28	29	30	31
#	Year	GB ID	1953	1953	1953	1953	1987	1992	1993	1994	2000	2001	2001	2001	2002	2002	2003	2003	2003	2003	2003	2004	2004	2004	2004	2005	2005	2005	2007	2008	2009	2012	2013
1	1953	AF534906.1																															
2	1953	NC_001405.1	0.016																														
3	1953	AC_000008.1	0.017	0.014																													
4	1953	FJ349096.1	0.013	0.014	0.011																												
5	1987	LC068713.1	0.013	0.014	0.011	0.001																											
6	1992	KF268310.1	0.015	0.001	0.013	0.013	0.013																										
7	1993	LC068714.1	0.012	0.013	0.011	0.001	0.001	0.012																									
8	1994	LC068715.1	0.013	0.014	0.011	0.001	0.001	0.013	0.001																								
9	2000	JX173078.1	0.000	0.016	0.018	0.012	0.012	0.016	0.012	0.012																							
10	2001	JX173080.1	0.003	0.018	0.019	0.014	0.014	0.017	0.013	0.014	0.003																						
11	2001	JX173081.1	0.018	0.016	0.014	0.012	0.012	0.015	0.012	0.012	0.018	0.020																					
12	2001	HQ003817.1	0.013	0.015	0.014	0.007	0.007	0.014	0.007	0.007	0.013	0.015	0.014																				
13	2002	JX173079.1	0.011	0.013	0.016	0.011	0.011	0.012	0.011	0.011	0.011	0.013	0.017	0.014																			
14	2002	KX384959.1	0.011	0.012	0.017	0.014	0.014	0.011	0.013	0.014	0.011	0.013	0.018	0.015	0.003																		
15	2003	JX173082.1	0.000	0.016	0.017	0.013	0.013	0.015	0.012	0.013	0.000	0.003	0.018	0.013	0.011	0.011																	
16	2003	JX173083.1	0.002	0.016	0.018	0.012	0.012	0.015	0.011	0.012	0.002	0.004	0.018	0.013	0.011	0.010	0.002																
17	2003	JX173085.1	0.002	0.016	0.018	0.012	0.012	0.015	0.011	0.012	0.002	0.004	0.018	0.013	0.011	0.010	0.002	0.000															
18	2003	JX173084.1	0.015	0.001	0.013	0.013	0.013	0.001	0.012	0.013	0.016	0.017	0.015	0.014	0.012	0.011	0.015	0.015	0.015														
19	2003	LC068716.1	0.013	0.014	0.011	0.001	0.001	0.013	0.001	0.001	0.013	0.014	0.013	0.007	0.012	0.014	0.013	0.012	0.012	0.013													
20	2004	JX173086.1	0.003	0.016	0.018	0.012	0.012	0.016	0.012	0.012	0.002	0.004	0.018	0.013	0.011	0.011	0.003	0.000	0.000	0.016	0.013												
21	2004	LC068717.1	0.013	0.014	0.012	0.001	0.001	0.013	0.002	0.001	0.012	0.014	0.012	0.007	0.011	0.014	0.013	0.012	0.012	0.013	0.001	0.012											
22	2004	LC068718.1	0.008	0.013	0.012	0.004	0.004	0.013	0.005	0.004	0.008	0.010	0.012	0.008	0.015	0.015	0.008	0.008	0.008	0.013	0.004	0.008	0.004										
23	2004	KF268130.1	0.016	0.002	0.013	0.013	0.013	0.001	0.013	0.013	0.016	0.018	0.016	0.014	0.012	0.012	0.016	0.016	0.016	0.001	0.014	0.016	0.013	0.013									
24	2005	JX173077.1	0.015	0.001	0.013	0.013	0.013	0.001	0.012	0.013	0.016	0.017	0.015	0.014	0.012	0.011	0.015	0.015	0.015	0.001	0.013	0.016	0.013	0.013	0.001								
25	2005	KF268129.1	0.018	0.019	0.017	0.014	0.014	0.019	0.014	0.014	0.018	0.020	0.020	0.016	0.017	0.018	0.018	0.018	0.018	0.019	0.015	0.018	0.014	0.015	0.018	0.019							
26	2005	LC068720.1	0.013	0.014	0.012	0.001	0.001	0.013	0.002	0.001	0.012	0.014	0.012	0.007	0.011	0.014	0.013	0.012	0.012	0.013	0.001	0.012	0.000	0.004	0.013	0.013	0.014						
27	2007	JX423389.1	0.013	0.014	0.011	0.001	0.001	0.013	0.001	0.001	0.012	0.014	0.012	0.007	0.011	0.014	0.013	0.012	0.012	0.013	0.001	0.012	0.001	0.004	0.013	0.013	0.014	0.001					
28	2008	KF268199.1	0.008	0.015	0.013	0.008	0.008	0.014	0.008	0.008	0.007	0.009	0.013	0.009	0.015	0.015	0.008	0.008	0.008	0.014	0.008	0.008	0.007	0.003	0.014	0.014	0.017	0.007	0.008				
29	2009	KR699642.1	0.003	0.017	0.019	0.014	0.014	0.016	0.013	0.014	0.002	0.005	0.019	0.015	0.012	0.011	0.003	0.003	0.003	0.016	0.014	0.003	0.014	0.010	0.017	0.016	0.018	0.014	0.014	0.009			
30	2012	MF315028	0.009	0.010	0.015	0.010	0.010	0.009	0.009	0.010	0.008	0.010	0.015	0.010	0.015	0.015	0.009	0.008	0.008	0.009	0.010	0.008	0.009	0.005	0.009	0.009	0.017	0.009	0.010	0.007	0.010		
31	2013	KF951595.1	0.014	0.015	0.012	0.002	0.002	0.014	0.002	0.002	0.013	0.015	0.013	0.008	0.012	0.015	0.014	0.013	0.013	0.014	0.002	0.013	0.002	0.005	0.014	0.014	0.016	0.002	0.002	0.009	0.015	0.011	
32	2013	MF315029	0.004	0.018	0.019	0.014	0.014	0.017	0.013	0.014	0.004	0.005	0.020	0.015	0.012	0.012	0.004	0.002	0.002	0.017	0.014	0.003	0.014	0.010	0.017	0.017	0.019	0.014	0.014	0.010	0.005	0.009	0.015

1: the lowest p-distance is highlighted for each sequence of viruses collected after 1953 and is reported in Supplementary Table S1.

Supplementary Table S7: pairwise p-distance for the hexon gene (nt 18838-21744) of the 32 analyzed genomes

			1 [1]	2	3	4	5	6	7	8	9	10	11	12	13	14	15	16	17	18	19	20	21	22	23	24	25	26	27	28	29	30	31
#	Year	GB ID	1953	1953	1953	1953	1987	1992	1993	1994	2000	2001	2001	2001	2002	2002	2003	2003	2003	2003	2003	2004	2004	2004	2004	2005	2005	2005	2007	2008	2009	2012	2013
1	1953	AF534906.1																															
2	1953	NC_001405.1	0.138																														
3	1953	AC_000008.1	0.151	0.156																													
4	1953	FJ349096.1	0.150	0.099	0.156																												
5	1987	LC068713.1	0.150	0.100	0.155	0.001																											
6	1992	KF268310.1	0.138	0.000	0.156	0.099	0.100																										
7	1993	LC068714.1	0.151	0.099	0.154	0.002	0.002	0.100																									
8	1994	LC068715.1	0.150	0.099	0.156	0.001	0.000	0.100	0.002																								
9	2000	JX173078.1	0.000	0.138	0.151	0.150	0.150	0.138	0.151	0.150																							
10	2001	JX173080.1	0.002	0.139	0.151	0.151	0.150	0.139	0.152	0.151	0.002																						
11	2001	JX173081.1	0.140	0.022	0.159	0.102	0.103	0.022	0.104	0.102	0.140	0.141																					
12	2001	HQ003817.1	0.113	0.108	0.139	0.108	0.107	0.108	0.107	0.108	0.113	0.114	0.110																				
13	2002	JX173079.1	0.142	0.021	0.162	0.092	0.092	0.021	0.092	0.092	0.141	0.142	0.028	0.116																			
14	2002	KX384959.1	0.142	0.021	0.163	0.092	0.092	0.021	0.092	0.092	0.142	0.142	0.028	0.115	0.000																		
15	2003	JX173082.1	0.001	0.139	0.151	0.150	0.150	0.138	0.151	0.150	0.001	0.003	0.141	0.114	0.142	0.142																	
16	2003	JX173083.1	0.002	0.138	0.150	0.149	0.149	0.137	0.150	0.149	0.002	0.003	0.140	0.112	0.141	0.141	0.003																
17	2003	JX173085.1	0.002	0.137	0.150	0.149	0.149	0.137	0.150	0.149	0.001	0.002	0.139	0.112	0.141	0.141	0.002	0.000															
18	2003	JX173084.1	0.139	0.000	0.157	0.099	0.100	0.001	0.100	0.100	0.138	0.139	0.022	0.109	0.020	0.021	0.139	0.138	0.138														
19	2003	LC068716.1	0.150	0.099	0.156	0.001	0.000	0.100	0.002	0.000	0.150	0.151	0.102	0.108	0.092	0.092	0.150	0.149	0.149	0.100													
20	2004	JX173086.1	0.002	0.137	0.150	0.150	0.149	0.137	0.151	0.150	0.002	0.003	0.139	0.112	0.141	0.141	0.003	0.001	0.000	0.137	0.150												
21	2004	LC068717.1	0.150	0.099	0.156	0.001	0.000	0.100	0.002	0.000	0.150	0.151	0.102	0.108	0.092	0.092	0.150	0.149	0.149	0.100	0.000	0.150											
22	2004	LC068718.1	0.150	0.099	0.156	0.001	0.000	0.100	0.002	0.000	0.150	0.151	0.102	0.108	0.092	0.092	0.150	0.149	0.149	0.100	0.000	0.150	0.000										
23	2004	KF268130.1	0.139	0.001	0.157	0.100	0.100	0.001	0.100	0.100	0.139	0.140	0.022	0.109	0.021	0.022	0.139	0.138	0.138	0.001	0.100	0.138	0.100	0.100									
24	2005	JX173077.1	0.138	0.000	0.156	0.099	0.100	0.000	0.099	0.099	0.138	0.139	0.022	0.108	0.021	0.021	0.139	0.138	0.137	0.000	0.099	0.137	0.099	0.099	0.001								
25	2005	KF268129.1	0.149	0.095	0.154	0.017	0.017	0.095	0.017	0.017	0.149	0.150	0.105	0.104	0.097	0.097	0.149	0.148	0.148	0.094	0.017	0.148	0.017	0.017	0.095	0.095							
26	2005	LC068720.1	0.150	0.099	0.156	0.001	0.000	0.100	0.002	0.000	0.150	0.151	0.102	0.108	0.092	0.092	0.150	0.149	0.149	0.100	0.000	0.150	0.000	0.000	0.100	0.099	0.017						
27	2007	JX423389.1	0.150	0.099	0.156	0.001	0.000	0.100	0.002	0.000	0.150	0.151	0.102	0.108	0.092	0.092	0.150	0.149	0.149	0.100	0.000	0.150	0.000	0.000	0.100	0.099	0.017	0.000					
28	2008	KF268199.1	0.152	0.160	0.008	0.156	0.156	0.160	0.158	0.156	0.151	0.151	0.159	0.143	0.165	0.165	0.151	0.150	0.151	0.160	0.156	0.150	0.156	0.156	0.161	0.160	0.156	0.156	0.156				
29	2009	KR699642.1	0.136	0.012	0.162	0.099	0.099	0.012	0.100	0.099	0.136	0.137	0.023	0.110	0.023	0.023	0.136	0.135	0.135	0.012	0.099	0.135	0.099	0.099	0.012	0.012	0.099	0.099	0.099	0.162			
30	2012	MF315028	0.139	0.001	0.157	0.100	0.100	0.001	0.100	0.100	0.139	0.140	0.022	0.109	0.021	0.022	0.139	0.138	0.138	0.001	0.100	0.138	0.100	0.100	0.001	0.001	0.095	0.100	0.100	0.161	0.012		
31	2013	KF951595.1	0.150	0.099	0.156	0.001	0.000	0.100	0.002	0.000	0.150	0.151	0.102	0.108	0.092	0.092	0.150	0.149	0.149	0.100	0.000	0.150	0.000	0.000	0.100	0.099	0.017	0.000	0.000	0.156	0.099	0.100	
32	2013	MF315029	0.136	0.011	0.162	0.099	0.099	0.012	0.100	0.099	0.135	0.136	0.023	0.109	0.023	0.023	0.136	0.135	0.135	0.012	0.099	0.135	0.099	0.099	0.012	0.011	0.099	0.099	0.099	0.162	0.000	0.011	0.099

1: the lowest p-distance is highlighted for each sequence of viruses collected after 1953 and is reported in Supplementary Table S1.

#	Type [1,2]		1	2	3	4	5	6	7	8	9	10	11	12	13	14	15	16	17	18	19	20	21	22	23	24	25	26	27	28	29	30	31
1	57	HQ003817.1-2001																															
2	5	KF268199.1-2008	0.143		type 5																												
3	5	AC_000008.1-1953	0.139	0.008																													
4	6	LC068718.1-2004	0.108	0.156	0.156											type 6 (0.003) [3]																	
5	6	LC068713.1-1987	0.107	0.156	0.155	0.000																											
6	6	LC068717.1-2004	0.108	0.156	0.156	0.000	0.000																										
7	6	LC068720.1-2005	0.108	0.156	0.156	0.000	0.000	0.000																									
8	6	LC068716.1-2003	0.108	0.156	0.156	0.000	0.000	0.000	0.000																								
9	6	KF268129.1-2005	0.104	0.156	0.154	0.017	0.017	0.017	0.017	0.017																							
10	6	LC068714.1-1993	0.107	0.158	0.154	0.002	0.002	0.002	0.002	0.002	0.017																						
11	6	LC068715.1-1994	0.108	0.156	0.156	0.000	0.000	0.000	0.000	0.000	0.017	0.002																					
12	6	KF951595.1-2013	0.108	0.156	0.156	0.000	0.000	0.000	0.000	0.000	0.017	0.002	0.000																				
13	6	FJ349096.1-1953	0.108	0.156	0.156	0.001	0.001	0.001	0.001	0.001	0.017	0.002	0.001	0.001																			
14	6	JX423389.1-2007	0.108	0.156	0.156	0.000	0.000	0.000	0.000	0.000	0.017	0.002	0.000	0.000	0.001																		
15	1	JX173078.1-2000	0.113	0.151	0.151	0.150	0.150	0.150	0.150	0.150	0.149	0.151	0.150	0.150	0.150	0.150								type 1 (0.002)									
16	1	JX173082.1-2003	0.114	0.151	0.151	0.150	0.150	0.150	0.150	0.150	0.149	0.151	0.150	0.150	0.150	0.150	0.001																
17	1	AF534906.1-1953	0.113	0.152	0.151	0.150	0.150	0.150	0.150	0.150	0.149	0.151	0.150	0.150	0.150	0.150	0.000	0.001															
18	1	JX173083.1-2003	0.112	0.150	0.150	0.149	0.149	0.149	0.149	0.149	0.148	0.150	0.149	0.149	0.149	0.149	0.002	0.003	0.002														
19	1	JX173080.1-2001	0.114	0.151	0.151	0.151	0.150	0.151	0.151	0.151	0.150	0.152	0.151	0.151	0.151	0.151	0.002	0.003	0.002	0.003													
20	1	JX173086.1-2004	0.112	0.150	0.150	0.150	0.149	0.150	0.150	0.150	0.148	0.151	0.150	0.150	0.150	0.150	0.002	0.003	0.002	0.001	0.003												
21	1	JX173085.1-2003	0.112	0.151	0.150	0.149	0.149	0.149	0.149	0.149	0.148	0.150	0.149	0.149	0.149	0.149	0.001	0.002	0.002	0.000	0.002	0.000											
22	2	JX173077.1-2005	0.108	0.160	0.156	0.099	0.100	0.099	0.099	0.099	0.095	0.099	0.099	0.099	0.099	0.099	0.138	0.139	0.138	0.138	0.139	0.137	0.137										type 2 (0.013)
23	2	JX173081.1-2001	0.110	0.159	0.159	0.102	0.103	0.102	0.102	0.102	0.105	0.104	0.102	0.102	0.102	0.102	0.140	0.141	0.140	0.140	0.141	0.139	0.139	0.022									
24	2	NC_001405.1-1953	0.108	0.160	0.156	0.099	0.100	0.099	0.099	0.099	0.095	0.099	0.099	0.099	0.099	0.099	0.138	0.139	0.138	0.138	0.139	0.137	0.137	0.000	0.022								
25	2	MF315029-2013	0.109	0.162	0.162	0.099	0.099	0.099	0.099	0.099	0.099	0.100	0.099	0.099	0.099	0.099	0.135	0.136	0.136	0.135	0.136	0.135	0.135	0.011	0.023	0.011							
26	2	MF315028-2012	0.109	0.161	0.157	0.100	0.100	0.100	0.100	0.100	0.095	0.100	0.100	0.100	0.100	0.100	0.139	0.139	0.139	0.138	0.140	0.138	0.138	0.001	0.022	0.001	0.011						
27	2	KX384959.1-2002	0.115	0.165	0.163	0.092	0.092	0.092	0.092	0.092	0.097	0.092	0.092	0.092	0.092	0.092	0.142	0.142	0.142	0.141	0.142	0.141	0.141	0.021	0.028	0.021	0.023	0.022					
28	2	KF268130.1-2004	0.109	0.161	0.157	0.100	0.100	0.100	0.100	0.100	0.095	0.100	0.100	0.100	0.100	0.100	0.139	0.139	0.139	0.138	0.140	0.138	0.138	0.001	0.022	0.001	0.012	0.001	0.022				
29	2	KF268310.1-1992	0.108	0.160	0.156	0.100	0.100	0.100	0.100	0.100	0.095	0.100	0.100	0.100	0.099	0.100	0.138	0.138	0.138	0.137	0.139	0.137	0.137	0.000	0.022	0.000	0.012	0.001	0.021	0.001			
30	2	JX173084.1-2003	0.109	0.160	0.157	0.100	0.100	0.100	0.100	0.100	0.094	0.100	0.100	0.100	0.099	0.100	0.138	0.139	0.139	0.138	0.139	0.137	0.138	0.000	0.022	0.000	0.012	0.001	0.021	0.001	0.001		
31	2	JX173079.1-2002	0.116	0.165	0.162	0.092	0.092	0.092	0.092	0.092	0.097	0.092	0.092	0.092	0.092	0.092	0.141	0.142	0.142	0.141	0.142	0.141	0.141	0.021	0.028	0.021	0.023	0.021	0.000	0.021	0.021	0.020	
32	2	KR699642.1-2009	0.110	0.162	0.162	0.099	0.099	0.099	0.099	0.099	0.099	0.100	0.099	0.099	0.099	0.099	0.136	0.136	0.136	0.135	0.137	0.135	0.135	0.012	0.023	0.012	0.000	0.012	0.023	0.012	0.012	0.012	0.023

1: sequences are sorted by type.
2: p-distance are color coded, from green (0) to red (0.151).
3: p-distance within each type is shown.

Supplementary Table S8: pairwise p-distance for the genomic region 21745-26000 of the 32 analyzed genomes

			1 [1]	2	3	4	5	6	7	8	9	10	11	12	13	14	15	16	17	18	19	20	21	22	23	24	25	26	27	28	29	30	31
#	Year	GB ID	1953	1953	1953	1953	1987	1992	1993	1994	2000	2001	2001	2001	2002	2002	2003	2003	2003	2003	2003	2004	2004	2004	2004	2005	2005	2005	2007	2008	2009	2012	2013
1	1953	AF534906.1																															
2	1953	NC_001405.1	0.020																														
3	1953	AC_000008.1	0.030	0.023																													
4	1953	FJ349096.1	0.018	0.004	0.026																												
5	1987	LC068713.1	0.022	0.016	0.025	0.014																											
6	1992	KF268310.1	0.020	0.001	0.023	0.005	0.017																										
7	1993	LC068714.1	0.023	0.017	0.025	0.015	0.001	0.017																									
8	1994	LC068715.1	0.023	0.017	0.025	0.015	0.001	0.017	0.000																								
9	2000	JX173078.1	0.002	0.021	0.031	0.019	0.022	0.020	0.023	0.023																							
10	2001	JX173080.1	0.006	0.021	0.032	0.019	0.023	0.020	0.024	0.024	0.007																						
11	2001	JX173081.1	0.018	0.014	0.028	0.017	0.023	0.014	0.023	0.023	0.019	0.018																					
12	2001	HQ003817.1	0.027	0.024	0.028	0.022	0.010	0.024	0.010	0.010	0.027	0.027	0.028																				
13	2002	JX173079.1	0.015	0.013	0.027	0.016	0.018	0.013	0.019	0.019	0.016	0.017	0.015	0.024																			
14	2002	KX384959.1	0.015	0.013	0.027	0.016	0.018	0.013	0.019	0.019	0.016	0.016	0.015	0.024	0.001																		
15	2003	JX173082.1	0.001	0.020	0.030	0.018	0.022	0.019	0.022	0.022	0.002	0.006	0.018	0.027	0.015	0.015																	
16	2003	JX173083.1	0.003	0.019	0.030	0.017	0.022	0.019	0.022	0.022	0.004	0.005	0.017	0.027	0.015	0.015	0.003																
17	2003	JX173085.1	0.004	0.019	0.030	0.018	0.022	0.019	0.023	0.023	0.005	0.005	0.018	0.027	0.016	0.016	0.004	0.000															
18	2003	JX173084.1	0.020	0.001	0.024	0.004	0.016	0.001	0.017	0.017	0.021	0.021	0.014	0.024	0.013	0.013	0.020	0.019	0.019														
19	2003	LC068716.1	0.023	0.017	0.025	0.015	0.001	0.018	0.000	0.000	0.023	0.024	0.024	0.010	0.019	0.019	0.023	0.023	0.023	0.017													
20	2004	JX173086.1	0.003	0.019	0.030	0.017	0.022	0.019	0.022	0.022	0.004	0.005	0.017	0.027	0.015	0.015	0.003	0.000	0.000	0.019	0.023												
21	2004	LC068717.1	0.022	0.016	0.025	0.014	0.000	0.017	0.001	0.001	0.022	0.024	0.023	0.010	0.018	0.018	0.022	0.022	0.022	0.017	0.001	0.022											
22	2004	LC068718.1	0.023	0.017	0.025	0.015	0.001	0.018	0.000	0.000	0.023	0.024	0.024	0.010	0.019	0.019	0.023	0.023	0.023	0.017	0.000	0.023	0.001										
23	2004	KF268130.1	0.020	0.001	0.024	0.005	0.016	0.001	0.017	0.017	0.021	0.021	0.014	0.024	0.013	0.013	0.020	0.019	0.020	0.001	0.017	0.019	0.016	0.017									
24	2005	JX173077.1	0.019	0.000	0.023	0.004	0.016	0.001	0.017	0.017	0.020	0.020	0.013	0.023	0.013	0.013	0.019	0.018	0.019	0.000	0.017	0.018	0.016	0.017	0.001								
25	2005	KF268129.1	0.030	0.028	0.024	0.028	0.025	0.029	0.025	0.025	0.031	0.032	0.033	0.027	0.031	0.031	0.030	0.030	0.031	0.028	0.026	0.030	0.025	0.026	0.029	0.028							
26	2005	LC068720.1	0.022	0.016	0.025	0.014	0.000	0.017	0.001	0.001	0.022	0.024	0.023	0.010	0.018	0.018	0.022	0.022	0.022	0.017	0.001	0.022	0.000	0.001	0.016	0.016	0.025						
27	2007	JX423389.1	0.023	0.017	0.025	0.015	0.001	0.017	0.001	0.001	0.023	0.024	0.023	0.011	0.019	0.019	0.022	0.022	0.023	0.017	0.001	0.022	0.001	0.001	0.017	0.017	0.025	0.001					
28	2008	KF268199.1	0.032	0.024	0.003	0.028	0.026	0.025	0.026	0.026	0.032	0.033	0.029	0.029	0.029	0.029	0.031	0.031	0.032	0.025	0.027	0.031	0.026	0.027	0.025	0.024	0.025	0.026	0.026				
29	2009	KR699642.1	0.013	0.016	0.029	0.016	0.023	0.016	0.023	0.023	0.014	0.014	0.021	0.027	0.017	0.017	0.013	0.012	0.012	0.016	0.024	0.012	0.023	0.024	0.016	0.015	0.031	0.023	0.023	0.031			
30	2012	MF315028	0.020	0.001	0.024	0.005	0.017	0.002	0.018	0.018	0.021	0.021	0.014	0.024	0.014	0.014	0.020	0.019	0.020	0.001	0.018	0.019	0.017	0.018	0.002	0.001	0.029	0.017	0.018	0.025	0.016		
31	2013	KF951595.1	0.022	0.016	0.025	0.014	0.000	0.017	0.001	0.001	0.022	0.023	0.023	0.010	0.018	0.018	0.022	0.022	0.022	0.016	0.001	0.022	0.000	0.001	0.016	0.016	0.025	0.000	0.001	0.026	0.023	0.017	
32	2013	MF315029	0.013	0.016	0.029	0.017	0.023	0.016	0.023	0.023	0.014	0.014	0.021	0.027	0.018	0.018	0.013	0.012	0.012	0.016	0.024	0.012	0.023	0.024	0.016	0.016	0.031	0.023	0.023	0.031	0.002	0.017	0.023

1: the lowest p-distance is highlighted for each sequence of viruses collected after 1953 and is reported in Supplementary Table S1.

Supplementary table S9: pairwise p-distance for the genomic region 26001-31029 of the 32 analyzed genomes

			1 [1]	2	3	4	5	6	7	8	9	10	11	12	13	14	15	16	17	18	19	20	21	22	23	24	25	26	27	28	29	30	31
#	Year	GB ID	1953	1953	1953	1953	1987	1992	1993	1994	2000	2001	2001	2001	2002	2002	2003	2003	2003	2003	2003	2004	2004	2004	2004	2005	2005	2005	2007	2008	2009	2012	2013
1	1953	AF534906.1																															
2	1953	NC_001405.1	0.099																														
3	1953	AC_000008.1	0.105	0.109																													
4	1953	FJ349096.1	0.100	0.008	0.108																												
5	1987	LC068713.1	0.100	**0.007**	0.107	**0.007**																											
6	1992	KF268310.1	0.099	**0.001**	0.109	0.008	0.007																										
7	1993	LC068714.1	0.100	0.006	0.107	0.007	**0.000**	0.006																									
8	1994	LC068715.1	0.100	0.007	0.108	0.008	**0.001**	0.007	**0.001**																								
9	2000	JX173078.1	**0.000**	0.099	0.105	0.100	0.100	0.099	0.100	0.101																							
10	2001	JX173080.1	0.095	**0.017**	0.111	0.018	0.018	**0.017**	0.018	0.019	0.095																						
11	2001	JX173081.1	0.096	**0.012**	0.111	0.017	0.014	**0.012**	0.014	0.015	0.097	0.014																					
12	2001	HQ003817.1	0.102	0.020	0.113	**0.013**	0.017	0.020	0.017	0.017	0.102	0.019	0.022																				
13	2002	JX173079.1	0.099	**0.004**	0.108	0.008	0.007	**0.004**	0.006	0.007	0.099	0.017	0.012	0.019																			
14	2002	KX384959.1	0.099	0.004	0.109	0.009	0.007	0.004	0.007	0.008	0.100	0.017	0.013	0.019	**0.001**																		
15	2003	JX173082.1	**0.000**	0.099	0.105	0.100	0.100	0.099	0.100	0.101	0.001	0.095	0.097	0.102	0.099	0.100																	
16	2003	JX173083.1	**0.003**	0.100	0.105	0.100	0.100	0.099	0.100	0.101	**0.003**	0.095	0.097	0.102	0.099	0.100	**0.003**																
17	2003	JX173085.1	0.003	0.100	0.106	0.101	0.100	0.100	0.100	0.101	0.003	0.096	0.097	0.102	0.100	0.100	0.003	**0.000**															
18	2003	JX173084.1	0.099	**0.001**	0.109	0.009	0.007	**0.001**	0.006	0.007	0.100	0.018	0.013	0.020	0.004	0.004	0.100	0.100	0.100														
19	2003	LC068716.1	0.100	0.007	0.108	0.007	0.001	0.007	**0.000**	0.001	0.100	0.018	0.014	0.017	0.007	0.007	0.100	0.100	0.101	0.007													
20	2004	JX173086.1	0.003	0.100	0.105	0.100	0.100	0.099	0.100	0.101	0.003	0.095	0.097	0.102	0.099	0.100	0.003	**0.000**	**0.000**	0.100	0.100												
21	2004	LC068717.1	0.100	0.006	0.108	0.007	**0.001**	0.006	**0.001**	**0.001**	0.100	0.018	0.014	0.017	0.006	0.007	0.100	0.100	0.101	0.007	**0.001**	0.100											
22	2004	LC068718.1	0.100	0.006	0.108	0.007	0.001	0.006	**0.000**	0.001	0.100	0.018	0.014	0.017	0.006	0.007	0.100	0.101	0.101	0.007	0.001	0.101	0.001										
23	2004	KF268130.1	0.099	**0.001**	0.109	0.009	0.007	**0.001**	0.006	0.007	0.099	0.018	0.012	0.020	0.004	0.004	0.099	0.100	0.100	0.002	0.007	0.100	0.007	0.007									
24	2005	JX173077.1	0.099	0.001	0.109	0.008	0.006	**0.000**	0.006	0.007	0.099	0.017	0.012	0.019	0.003	0.004	0.099	0.099	0.099	0.001	0.006	0.099	0.006	0.006	0.001								
25	2005	KF268129.1	0.100	0.082	0.116	0.082	0.083	0.082	0.083	0.083	0.100	0.084	0.085	0.087	**0.081**	0.082	0.100	0.101	0.101	0.083	0.083	0.101	0.082	0.083	0.082	0.082							
26	2005	LC068720.1	0.099	0.006	0.107	0.007	0.001	0.006	0.001	0.001	0.100	0.018	0.014	0.017	0.006	0.007	0.100	0.100	0.100	0.007	0.001	0.100	**0.000**	0.001	0.007	0.006	0.082						
27	2007	JX423389.1	0.100	0.006	0.107	0.007	**0.000**	0.006	**0.000**	0.001	0.100	0.018	0.014	0.017	0.006	0.007	0.100	0.100	0.100	0.006	**0.000**	0.100	0.001	**0.000**	0.006	0.006	0.083	0.001					
28	2008	KF268199.1	0.104	0.110	**0.006**	0.109	0.108	0.110	0.108	0.109	0.104	0.111	0.112	0.113	0.109	0.110	0.104	0.105	0.105	0.110	0.109	0.105	0.108	0.109	0.110	0.110	0.115	0.108	0.108				
29	2009	KR699642.1	0.099	**0.004**	0.108	0.010	0.006	**0.004**	0.005	0.006	0.099	0.018	0.013	0.021	0.005	0.005	0.099	0.100	0.100	**0.004**	0.006	0.100	0.006	0.006	**0.004**	**0.004**	0.083	0.006	0.005	0.109			
30	2012	MF315028	0.100	**0.002**	0.110	0.009	0.007	**0.002**	0.007	0.008	0.100	0.018	0.013	0.021	0.004	0.005	0.100	0.100	0.101	0.003	0.007	0.100	0.007	0.007	0.003	**0.002**	0.083	0.007	0.007	0.111	0.005		
31	2013	KF951595.1	0.100	0.007	0.108	0.007	0.001	0.007	**0.000**	0.001	0.100	0.018	0.014	0.017	0.007	0.007	0.100	0.101	0.101	0.007	0.001	0.101	0.001	0.001	0.007	0.006	0.083	0.001	**0.000**	0.109	0.006	0.007	
32	2013	MF315029	0.100	0.004	0.109	0.010	0.006	0.004	0.005	0.006	0.100	0.018	0.013	0.021	0.005	0.005	0.100	0.100	0.100	0.004	0.006	0.100	0.006	0.006	0.004	0.004	0.083	0.006	0.005	0.110	**0.001**	0.004	0.006

1: the lowest p-distance is highlighted for each sequence of viruses collected after 1953 and is reported in Supplementary Table S1.

Supplementary Table S10: pairwise p-distance for the fiber gene (31030-32778) of the 32 analyzed genomes

			1 [1]	2	3	4	5	6	7	8	9	10	11	12	13	14	15	16	17	18	19	20	21	22	23	24	25	26	27	28	29	30	31
#	Year	GB ID	1953	1953	1953	1953	1987	1992	1993	1994	2000	2001	2001	2001	2002	2002	2003	2003	2003	2003	2003	2004	2004	2004	2004	2005	2005	2005	2007	2008	2009	2012	2013
1	1953	AF534906.1																															
2	1953	NC_001405.1	0.268																														
3	1953	AC_000008.1	0.249	0.269																													
4	1953	FJ349096.1	0.224	0.246	0.282																												
5	1987	LC068713.1	0.224	0.248	0.284	0.004																											
6	1992	KF268310.1	0.267	0.001	0.269	0.246	0.249																										
7	1993	LC068714.1	0.224	0.248	0.284	0.004	0.000	0.249																									
8	1994	LC068715.1	0.224	0.248	0.284	0.004	0.000	0.249	0.000																								
9	2000	JX173078.1	0.001	0.269	0.249	0.225	0.225	0.268	0.225	0.225																							
10	2001	JX173080.1	0.010	0.266	0.245	0.221	0.221	0.265	0.221	0.221	0.009																						
11	2001	JX173081.1	0.268	0.004	0.269	0.247	0.250	0.005	0.250	0.250	0.269	0.266																					
12	2001	HQ003817.1	0.226	0.250	0.285	0.004	0.001	0.250	0.001	0.001	0.226	0.222	0.251																				
13	2002	JX173079.1	0.268	0.003	0.267	0.246	0.248	0.005	0.248	0.248	0.269	0.266	0.005	0.250																			
14	2002	KX384959.1	0.267	0.003	0.267	0.247	0.250	0.005	0.250	0.250	0.268	0.265	0.005	0.251	0.001																		
15	2003	JX173082.1	0.001	0.268	0.248	0.224	0.224	0.268	0.224	0.224	0.001	0.010	0.269	0.226	0.269	0.268																	
16	2003	JX173083.1	0.008	0.266	0.248	0.222	0.222	0.266	0.222	0.222	0.007	0.007	0.267	0.224	0.267	0.266	0.007																
17	2003	JX173085.1	0.007	0.266	0.248	0.222	0.222	0.265	0.222	0.222	0.006	0.006	0.266	0.223	0.266	0.265	0.007	0.001															
18	2003	JX173084.1	0.267	0.001	0.269	0.246	0.249	0.000	0.249	0.249	0.268	0.265	0.005	0.250	0.005	0.005	0.268	0.266	0.265														
19	2003	LC068716.1	0.224	0.248	0.284	0.004	0.000	0.249	0.000	0.000	0.225	0.221	0.250	0.001	0.248	0.250	0.224	0.222	0.222	0.249													
20	2004	JX173086.1	0.008	0.266	0.248	0.222	0.222	0.266	0.222	0.222	0.007	0.007	0.267	0.224	0.267	0.266	0.007	0.001	0.001	0.266	0.222												
21	2004	LC068717.1	0.224	0.248	0.282	0.006	0.002	0.248	0.002	0.002	0.224	0.220	0.249	0.003	0.248	0.249	0.224	0.222	0.221	0.248	0.002	0.222											
22	2004	LC068718.1	0.224	0.248	0.284	0.004	0.000	0.249	0.000	0.000	0.225	0.221	0.250	0.001	0.248	0.250	0.224	0.222	0.222	0.249	0.000	0.222	0.002										
23	2004	KF268130.1	0.268	0.001	0.270	0.246	0.248	0.002	0.248	0.248	0.269	0.266	0.005	0.250	0.004	0.004	0.269	0.267	0.266	0.002	0.248	0.267	0.248	0.248									
24	2005	JX173077.1	0.267	0.001	0.269	0.246	0.249	0.000	0.249	0.249	0.268	0.265	0.005	0.250	0.005	0.005	0.268	0.266	0.265	0.000	0.249	0.266	0.248	0.249	0.002								
25	2005	KF268129.1	0.222	0.245	0.281	0.018	0.020	0.246	0.020	0.020	0.223	0.219	0.246	0.020	0.245	0.246	0.222	0.221	0.220	0.246	0.020	0.221	0.021	0.020	0.245	0.246							
26	2005	LC068720.1	0.224	0.248	0.283	0.004	0.001	0.248	0.001	0.001	0.224	0.220	0.249	0.002	0.248	0.249	0.224	0.222	0.221	0.248	0.001	0.222	0.001	0.001	0.248	0.248	0.020						
27	2007	JX423389.1	0.224	0.249	0.283	0.004	0.001	0.250	0.001	0.001	0.224	0.221	0.250	0.002	0.249	0.250	0.224	0.223	0.222	0.250	0.001	0.223	0.003	0.001	0.249	0.250	0.020	0.001					
28	2008	KF268199.1	0.250	0.271	0.002	0.284	0.285	0.271	0.285	0.285	0.250	0.246	0.270	0.286	0.268	0.268	0.250	0.250	0.249	0.271	0.285	0.250	0.283	0.285	0.271	0.271	0.282	0.284	0.284				
29	2009	KR699642.1	0.268	0.003	0.269	0.246	0.248	0.004	0.248	0.248	0.269	0.266	0.005	0.250	0.004	0.004	0.268	0.266	0.266	0.004	0.248	0.266	0.248	0.248	0.003	0.004	0.245	0.248	0.249	0.271			
30	2012	MF315028	0.268	0.001	0.270	0.246	0.249	0.002	0.249	0.249	0.269	0.266	0.003	0.250	0.003	0.003	0.269	0.267	0.266	0.002	0.249	0.267	0.248	0.249	0.001	0.002	0.246	0.248	0.250	0.271	0.002		
31	2013	KF951595.1	0.224	0.248	0.284	0.004	0.000	0.249	0.000	0.000	0.225	0.221	0.250	0.001	0.248	0.250	0.224	0.222	0.222	0.249	0.000	0.222	0.002	0.000	0.248	0.249	0.020	0.001	0.001	0.285	0.248	0.249	
32	2013	MF315029	0.268	0.002	0.270	0.246	0.249	0.003	0.249	0.249	0.269	0.266	0.003	0.250	0.003	0.003	0.269	0.267	0.266	0.003	0.249	0.267	0.248	0.249	0.002	0.003	0.246	0.248	0.250	0.271	0.001	0.001	0.249

1: the lowest p-distance is highlighted for each sequence of viruses collected after 1953 and is reported in Supplementary Table S1.

	Type [1,2]		1	2	3	4	5	6	7	8	9	10	11	12	13	14	15	16	17	18	19	20	21	22	23	24	25	26	27	28	29	30	31	
1	5	AC_000008.1-1953																																
2	5	KF268199.1-2008	0.002																															type 5
3	6	LC068718.1-2004	0.284	0.285																														
4	6	LC068713.1-1987	0.284	0.285	0.000																													type 6 (0.004) [3]
5	6	LC068717.1-2004	0.282	0.283	0.002	0.002																												
6	6	LC068720.1-2005	0.283	0.284	0.001	0.001	0.001																											
7	6	LC068714.1-1993	0.284	0.285	0.000	0.000	0.002	0.001																										
8	6	HQ003817.1-2001	0.285	0.286	0.001	0.001	0.003	0.002	0.001																									
9	6	KF268129.1-2005	0.281	0.282	0.020	0.020	0.021	0.020	0.020	0.020																								
10	6	LC068715.1-1994	0.284	0.285	0.000	0.000	0.002	0.001	0.000	0.001	0.020																							
11	6	LC068716.1-2003	0.284	0.285	0.000	0.000	0.002	0.001	0.000	0.001	0.020	0.000																						
12	6	KF951595.1-2013	0.284	0.285	0.000	0.000	0.002	0.001	0.000	0.001	0.020	0.000	0.000																					
13	6	FJ349096.1-1953	0.282	0.284	0.004	0.004	0.006	0.004	0.004	0.004	0.018	0.004	0.004	0.004																				
14	6	JX423389.1-2007	0.283	0.284	0.001	0.001	0.003	0.001	0.001	0.002	0.020	0.001	0.001	0.001	0.004																			
15	1	JX173078.1-2000	0.249	0.250	0.225	0.225	0.224	0.224	0.225	0.226	0.223	0.225	0.225	0.225	0.225	0.224																		
16	1	JX173082.1-2003	0.248	0.250	0.224	0.224	0.224	0.224	0.224	0.226	0.222	0.224	0.224	0.224	0.224	0.224	0.001																	type 1 (0.006)
17	1	AF534906.1-1953	0.249	0.250	0.224	0.224	0.224	0.224	0.224	0.226	0.222	0.224	0.224	0.224	0.224	0.224	0.001	0.001																
18	1	JX173083.1-2003	0.248	0.250	0.222	0.222	0.222	0.222	0.222	0.224	0.221	0.222	0.222	0.222	0.222	0.223	0.007	0.007	0.008															
19	1	JX173080.1-2001	0.245	0.246	0.221	0.221	0.220	0.220	0.221	0.222	0.219	0.221	0.221	0.221	0.221	0.221	0.009	0.010	0.010	0.007														
20	1	JX173086.1-2004	0.248	0.250	0.222	0.222	0.222	0.222	0.222	0.224	0.221	0.222	0.222	0.222	0.222	0.223	0.007	0.007	0.008	0.001	0.007													
21	1	JX173085.1-2003	0.248	0.249	0.222	0.222	0.221	0.221	0.222	0.223	0.220	0.222	0.222	0.222	0.222	0.222	0.006	0.007	0.007	0.001	0.006	0.001												
22	2	JX173077.1-2005	0.269	0.271	0.249	0.249	0.248	0.248	0.249	0.250	0.246	0.249	0.249	0.249	0.246	0.250	0.268	0.268	0.267	0.266	0.265	0.266	0.265											
23	2	JX173081.1-2001	0.269	0.270	0.250	0.250	0.249	0.249	0.250	0.251	0.246	0.250	0.250	0.250	0.247	0.250	0.269	0.269	0.268	0.267	0.266	0.267	0.266	0.005										type 2 (0.003)
24	2	NC_001405.1-1953	0.269	0.271	0.248	0.248	0.248	0.248	0.248	0.250	0.245	0.248	0.248	0.248	0.246	0.249	0.269	0.268	0.268	0.266	0.266	0.266	0.266	0.001	0.004									
25	2	MF315029-2013	0.270	0.271	0.249	0.249	0.248	0.248	0.249	0.250	0.246	0.249	0.249	0.249	0.246	0.250	0.269	0.269	0.268	0.267	0.266	0.267	0.266	0.003	0.003	0.002								
26	2	MF315028-2012	0.270	0.271	0.249	0.249	0.248	0.248	0.249	0.250	0.246	0.249	0.249	0.249	0.246	0.250	0.269	0.269	0.268	0.267	0.266	0.267	0.266	0.002	0.003	0.001	0.001							
27	2	KX384959.1-2002	0.267	0.268	0.250	0.250	0.249	0.249	0.250	0.251	0.246	0.250	0.250	0.250	0.247	0.250	0.268	0.268	0.267	0.266	0.265	0.266	0.265	0.005	0.005	0.003	0.003	0.003						
28	2	KF268130.1-2004	0.270	0.271	0.248	0.248	0.248	0.248	0.248	0.250	0.245	0.248	0.248	0.248	0.246	0.249	0.269	0.269	0.268	0.267	0.266	0.267	0.266	0.002	0.005	0.001	0.002	0.001	0.004					
29	2	KF268310.1-1992	0.269	0.271	0.249	0.249	0.248	0.248	0.249	0.250	0.246	0.249	0.249	0.249	0.246	0.250	0.268	0.268	0.267	0.266	0.265	0.266	0.265	0.000	0.005	0.001	0.003	0.002	0.005	0.002				
30	2	JX173084.1-2003	0.269	0.271	0.249	0.249	0.248	0.248	0.249	0.250	0.246	0.249	0.249	0.249	0.246	0.250	0.268	0.268	0.267	0.266	0.265	0.266	0.265	0.000	0.005	0.001	0.003	0.002	0.005	0.002	0.000			
31	2	JX173079.1-2002	0.267	0.268	0.248	0.248	0.248	0.248	0.248	0.250	0.245	0.248	0.248	0.248	0.246	0.249	0.269	0.269	0.268	0.267	0.266	0.267	0.266	0.005	0.005	0.003	0.003	0.003	0.001	0.004	0.005	0.005		
32	2	KR699642.1-2009	0.269	0.271	0.248	0.248	0.248	0.248	0.248	0.250	0.245	0.248	0.248	0.248	0.246	0.249	0.269	0.268	0.268	0.266	0.266	0.266	0.266	0.004	0.005	0.003	0.001	0.002	0.004	0.003	0.004	0.004	0.004	

1: The sequences are sorted by type.
2: the p-distances are color-coded, from green (0) to red (0.286).
3: the p-distance within type is indicated.

Supplementary Table S11: pairwise p-distance for the genomic region 32779-end of the 32 analyzed genomes

			1[1]	2	3	4	5	6	7	8	9	10	11	12	13	14	15	16	17	18	19	20	21	22	23	24	25	26	27	28	29	30	31
#	Year	GB ID	1953	1953	1953	1953	1987	1992	1993	1994	2000	2001	2001	2001	2002	2002	2003	2003	2003	2003	2003	2004	2004	2004	2004	2005	2005	2005	2007	2008	2009	2012	2013
1	1953	AF534906.1																															
2	1953	NC_001405.1	0.003																														
3	1953	AC_000008.1	0.011	0.011																													
4	1953	FJ349096.1	0.007	0.005	0.012																												
5	1987	LC068713.1	0.017	0.015	0.017	0.016																											
6	1992	KF268310.1	0.004	0.001	0.011	0.005	0.015																										
7	1993	LC068714.1	0.017	0.015	0.017	0.014	0.011	0.016																									
8	1994	LC068715.1	0.017	0.015	0.017	0.014	0.010	0.015	0.001																								
9	2000	JX173078.1	0.002	0.002	0.010	0.005	0.016	0.003	0.016	0.015																							
10	2001	JX173080.1	0.016	0.016	0.019	0.015	0.017	0.016	0.012	0.012	0.015																						
11	2001	JX173081.1	0.014	0.013	0.010	0.015	0.017	0.013	0.018	0.018	0.014	0.022																					
12	2001	HQ003817.1	0.010	0.009	0.016	0.011	0.015	0.009	0.010	0.010	0.009	0.011	0.017																				
13	2002	JX173079.1	0.013	0.011	0.012	0.012	0.018	0.011	0.018	0.018	0.012	0.020	0.015	0.017																			
14	2002	KX384959.1	0.014	0.012	0.013	0.013	0.019	0.012	0.020	0.019	0.013	0.021	0.016	0.018	0.002																		
15	2003	JX173082.1	0.001	0.003	0.010	0.006	0.017	0.003	0.017	0.016	0.001	0.016	0.014	0.010	0.013	0.014																	
16	2003	JX173083.1	0.008	0.006	0.006	0.008	0.016	0.006	0.014	0.014	0.007	0.014	0.009	0.010	0.011	0.012	0.007																
17	2003	JX173085.1	0.008	0.006	0.006	0.008	0.016	0.006	0.014	0.014	0.007	0.014	0.009	0.010	0.011	0.012	0.007	0.000															
18	2003	JX173084.1	0.003	0.001	0.011	0.005	0.015	0.001	0.015	0.015	0.002	0.016	0.013	0.009	0.011	0.013	0.003	0.006	0.006														
19	2003	LC068716.1	0.017	0.015	0.015	0.014	0.010	0.014	0.011	0.010	0.015	0.016	0.016	0.014	0.017	0.018	0.016	0.014	0.014	0.015													
20	2004	JX173086.1	0.007	0.007	0.005	0.009	0.017	0.007	0.015	0.015	0.006	0.013	0.010	0.011	0.012	0.013	0.006	0.001	0.001	0.007	0.015												
21	2004	LC068717.1	0.014	0.012	0.018	0.011	0.012	0.012	0.007	0.006	0.013	0.010	0.019	0.006	0.019	0.020	0.013	0.013	0.013	0.012	0.012	0.014											
22	2004	LC068718.1	0.017	0.015	0.015	0.014	0.004	0.015	0.010	0.010	0.015	0.016	0.015	0.014	0.016	0.017	0.016	0.014	0.014	0.015	0.008	0.015	0.011										
23	2004	KF268130.1	0.003	0.001	0.011	0.005	0.015	0.002	0.016	0.015	0.002	0.016	0.013	0.009	0.012	0.013	0.003	0.006	0.006	0.000	0.015	0.007	0.012	0.015									
24	2005	JX173077.1	0.004	0.002	0.012	0.006	0.016	0.001	0.016	0.016	0.003	0.016	0.014	0.010	0.012	0.013	0.003	0.007	0.007	0.002	0.016	0.008	0.013	0.016	0.002								
25	2005	KF268129.1	0.018	0.017	0.016	0.016	0.013	0.017	0.013	0.013	0.018	0.016	0.019	0.016	0.017	0.019	0.018	0.016	0.016	0.017	0.008	0.017	0.015	0.010	0.017	0.018							
26	2005	LC068720.1	0.014	0.012	0.018	0.011	0.012	0.012	0.007	0.006	0.013	0.010	0.019	0.006	0.019	0.020	0.013	0.013	0.013	0.012	0.012	0.014	0.000	0.011	0.012	0.013	0.015						
27	2007	JX423389.1	0.015	0.013	0.019	0.012	0.013	0.013	0.009	0.008	0.014	0.011	0.020	0.008	0.020	0.021	0.014	0.014	0.014	0.013	0.013	0.015	0.001	0.012	0.013	0.014	0.017	0.001					
28	2008	KF268199.1	0.008	0.008	0.006	0.009	0.018	0.008	0.016	0.016	0.007	0.015	0.010	0.011	0.012	0.014	0.007	0.002	0.002	0.008	0.016	0.001	0.014	0.016	0.008	0.009	0.017	0.014	0.016				
29	2009	KR699642.1	0.010	0.009	0.008	0.010	0.015	0.009	0.016	0.016	0.010	0.017	0.007	0.013	0.012	0.013	0.010	0.004	0.004	0.009	0.014	0.005	0.015	0.013	0.009	0.010	0.016	0.015	0.016	0.006			
30	2012	MF315028	0.019	0.017	0.017	0.016	0.012	0.017	0.013	0.012	0.018	0.017	0.019	0.016	0.018	0.020	0.018	0.016	0.016	0.017	0.010	0.017	0.013	0.010	0.017	0.018	0.012	0.013	0.015	0.018	0.016		
31	2013	KF951595.1	0.014	0.012	0.019	0.011	0.013	0.013	0.008	0.007	0.013	0.010	0.020	0.007	0.019	0.021	0.014	0.013	0.013	0.012	0.013	0.014	0.002	0.012	0.013	0.013	0.016	0.002	0.003	0.015	0.016	0.014	
32	2013	MF315029	0.010	0.008	0.007	0.010	0.014	0.008	0.015	0.015	0.009	0.017	0.007	0.013	0.011	0.013	0.010	0.004	0.004	0.008	0.013	0.005	0.014	0.012	0.009	0.009	0.015	0.014	0.016	0.005	0.001	0.016	0.016

1: the lowest p-distance is highlighted for each sequence of viruses collected after 1953 and is reported in Supplementary Table S1.

Supplementary Table S12: Overall mean p-distance for WGS as well as the genomic regions

**Genomic region**	**Overall mean distance**
WGS	**0.032** [1]
1-7000	0.009
7001-14150	0.009
14151-15866 Penton	0.009
15867-18837	0.011
18838-21744 Hexon	**0.099**
21745-26000	0.019
26001-31029	0.051
31030-32778 Fiber	**0.183**
32779-end	0.013

1: The p-distance in bold face are mentioned in the main text.

Supplementary Table S13: list of 117 recombination events identifed by RDP4 analysis package

		Breakpoint Positions									Detection Methods						
		In Alignment		In Recombinant Sequence		Relative to NC_001405.1-1953											
Recombination Event Number	Number In .RDP File	Begin	End	Begin	End	Begin	End	Recombinant Sequence(s)	Minor Parental Sequence(s)	Major Parental Sequence(s)	RDP	GENECONV	Bootscan	Maxchi	Chimaera	SiSscan	3Seq
1 [a]	1	28210	31548	28060	31060	28060	31054	JX173080.1-2001	KF268130.1-2004	JX173083.1-2003	7.34E-186	1.25E-189	1.03E-183	1.60E-49	3.40E-51	5.81E-53	1.65E-12
1									NC_001405.1-1953	AF534906.1-1953							
1									JX173077.1-2005	JX173082.1-2003							
1									KF268310.1-1992	JX173078.1-2000							
1									JX173084.1-2003	JX173085.1-2003							
1									KR699642.1-2009	JX173086.1-2004							
1									JX173079.1-2002								
1									KX384959.1-2002								
1									JX173081.1-2001								
1									FJ349096.1-1953								
1									JX423389.1-2007								
1									KF951595.1-2013								
1									LC068717.1-2004								
1									LC068720.1-2005								
1									LC068718.1-2004								
1									LC068713.1-1987								
1									LC068714.1-1993								
1									LC068715.1-1994								
1									LC068716.1-2003								
1									MF315028-2012								
1									MF315029-2013								
2	2	27476	31538	27343	31256	27327	31044	KF268129.1-2005	Unknown (MF315029-2013)	LC068716.1-2003	9.04E-117	1.03E-117	1.05E-126	2.12E-46	2.02E-47	1.52E-55	1.10E-12
2									Unknown(NC_001405.1-1953)	FJ349096.1-1953							
2									Unknown(JX173077.1-2005)	JX423389.1-2007							
2									Unknown(KF268310.1-1992)	KF951595.1-2013							
2									Unknown(KF268130.1-2004)	LC068717.1-2004							
2									Unknown(JX173084.1-2003)	LC068720.1-2005							
2									Unknown(KR699642.1-2009)	LC068718.1-2004							
2									Unknown(JX173079.1-2002)	LC068713.1-1987							
2									Unknown(KX384959.1-2002)	LC068714.1-1993							
2									Unknown(MF315028-2012)	LC068715.1-1994							

Supplementary Table S13: list of 117 recombination events identifed by RDP4 analysis package

		Breakpoint Positions									Detection Methods						
		In Alignment		In Recombinant Sequence		Relative to NC_001405.1-1953											
Recombination Event Number	Number In .RDP File	Begin	End	Begin	End	Begin	End	Recombinant Sequence(s)	Minor Parental Sequence(s)	Major Parental Sequence(s)	RDP	GENECONV	Bootscan	Maxchi	Chimaera	SiSscan	3Seq
3	3	4545	19299	4514	19228	4505	19200	^MF315029-2013	JX173085.1-2003	JX173081.1-2001	5.43E-118	3.96E-114	6.66E-66	3.65E-31	3.99E-09	3.84E-29	2.20E-12
3								KR699642.1-2009[P]	JX173082.1-2003								
3									JX173083.1-2003								
3									JX173086.1-2004								
4	4	27476	31546	27307	31030	27327	31052	^JX423389.1-2007	MF315029-2013	JX173083.1-2003	5.87E-98	4.50E-115	3.58E-112	3.26E-37	2.44E-28	1.45E-56	5.51E-13
4								FJ349096.1-1953	NC_001405.1-1953	AF534906.1-1953							
4								KF951595.1-2013	JX173077.1-2005	JX173082.1-2003							
4								LC068717.1-2004	KF268310.1-1992	JX173078.1-2000							
4								LC068720.1-2005	KF268130.1-2004	JX173085.1-2003							
4								LC068718.1-2004	JX173084.1-2003	JX173086.1-2004							
4								LC068713.1-1987	KR699642.1-2009								
4								LC068714.1-1993	JX173079.1-2002								
4								LC068715.1-1994	KX384959.1-2002								
4								LC068716.1-2003	MF315028-2012								
4								HQ003817.1-2001									
5	5	4130*	18467	4106*	18390	4090*	18370	^MF315028-2012	JX173085.1-2003	JX173081.1-2001	1.01E-101	5.45E-88	1.65E-78	4.53E-27	5.73E-15	8.97E-27	5.51E-13
5									JX173082.1-2003								
5									JX173083.1-2003								
5									JX173086.1-2004								
6	6	28155	33509	28005	32999	28005	32999	^NC_001405.1-1953	JX173081.1-2001	Unknown (KF268199.1-2008)	2.53E-67	1.44E-58	7.44E-66	4.69E-37	1.61E-15	3.80E-94	1.01E-77
6								JX173077.1-2005									
6								KF268310.1-1992									
6								KF268130.1-2004									
6								JX173084.1-2003									
6								MF315028-2012[P]									
7	7	27100	33252	26942	32622	26951	32743	HQ003817.1-2001	KF268129.1-2005	AF534906.1-1953	3.04E-55	3.04E-67	3.53E-71	3.99E-05	5.16E-15	9.19E-47	1.58E-03
7									FJ349096.1-1953	JX173082.1-2003							
7										JX173078.1-2000							
7										JX173083.1-2003							

Supplementary Table S13: list of 117 recombination events identifed by RDP4 analysis package

		Breakpoint Positions															
		In Alignment		In Recombinant Sequence		Relative to NC_001405.1-1953					Detection Methods						
Recombination Event Number	Number In .RDP File	Begin	End	Begin	End	Begin	End	Recombinant Sequence(s)	Minor Parental Sequence(s)	Major Parental Sequence(s)	RDP	GENECONV	Bootscan	Maxchi	Chimaera	SiSscan	3Seq
7										JX173085.1-2003							
7										JX173086.1-2004							
7										JX173080.1-2001							
8	8	4475	19227	4439	19121	4435	19128	^JX173081.1-2001	Unknown (JX173086.1-2004)	JX173079.1-2002	6.57E-67	1.74E-47	6.97E-36	1.68E-24	5.61E-10	3.36E-20	1.07E-04
8									Unknown(JX173082.1-2003)	KX384959.1-2002							
8									Unknown(JX173085.1-2003)								
9	9	5544	18460	5529	18396	5504	18363	^KF268129.1-2005	Unknown (JX173083.1-2003)	LC068718.1-2004	5.65E-63	1.25E-48	3.76E-26	1.13E-25	1.08E-07	3.05E-12	2.23E-05
9									Unknown(AF534906.1-1953)	FJ349096.1-1953							
9									Unknown(JX173082.1-2003)	JX423389.1-2007							
9									Unknown(JX173085.1-2003)	KF951595.1-2013							
9									Unknown(JX173086.1-2004)	LC068717.1-2004							
9									Unknown(JX173080.1-2001)	LC068720.1-2005							
9										LC068713.1-1987							
9										LC068714.1-1993							
9										LC068715.1-1994							
9										LC068716.1-2003							
9										KF268199.1-2008							
10	10	22491	26225	22381	26113	22362	26094	^KF268129.1-2005	Unknown (JX173077.1-2005)	FJ349096.1-1953	NS	4.47E-61	4.46E-07	3.30E-21	7.40E-21	1.29E-18	1.10E-12
10									Unknown(JX173081.1-2001)								
11	11	20456	21924	20308	21775	20327	21795	^JX423389.1-2007	KX384959.1-2002	JX173083.1-2003	1.23E-43	8.38E-55	5.41E-50	1.64E-16	2.37E-14	1.17E-27	5.51E-13
11								FJ349096.1-1953	JX173079.1-2002	AF534906.1-1953							
11								KF951595.1-2013	JX173081.1-2001	JX173082.1-2003							
11								LC068717.1-2004		JX173078.1-2000							
11								LC068720.1-2005		JX173085.1-2003							
11								LC068718.1-2004		JX173086.1-2004							
11								LC068713.1-1987		JX173080.1-2001							
11								LC068714.1-1993									
11								LC068715.1-1994									
11								LC068716.1-2003									

Supplementary Table S13: list of 117 recombination events identifed by RDP4 analysis package

		Breakpoint Positions									Detection Methods						
		In Alignment		In Recombinant Sequence		Relative to NC_001405.1-1953											
Recombination Event Number	Number In .RDP File	Begin	End	Begin	End	Begin	End	Recombinant Sequence(s)	Minor Parental Sequence(s)	Major Parental Sequence(s)	RDP	GENECONV	Bootscan	Maxchi	Chimaera	SiSscan	3Seq
11								KF268129.1-2005[P]									
12	12	23373	26204	23227	26058	23242	26073	FJ349096.1-1953	JX173077.1-2005	LC068718.1-2004	NS	4.39E-41	NS	3.52E-14	5.20E-13	1.70E-12	1.10E-12
12									KR699642.1-2009	JX423389.1-2007							
12										KF951595.1-2013							
12										LC068717.1-2004							
12										LC068720.1-2005							
12										LC068713.1-1987							
12										LC068714.1-1993							
12										LC068715.1-1994							
12										LC068716.1-2003							
13	13	598*	11260	597*	11170	596*	11183	^JX423389.1-2007	Unknown (KX384959.1-2002)	KF951595.1-2013	3.64E-15	1.90E-26	9.03E-31	4.81E-13	2.70E-12	3.62E-09	7.69E-35
13										LC068717.1-2004							
13										LC068720.1-2005							
13										LC068713.1-1987							
13										LC068714.1-1993							
13										LC068715.1-1994							
13										LC068716.1-2003							
14	14	1*	4474*	0*	4438*	1*	4434*	^JX173081.1-2001	JX173080.1-2001	MF315029-2013	1.55E-41	3.81E-35	3.34E-34	9.48E-13	7.47E-14	6.81E-14	1.65E-12
14										KR699642.1-2009							
15	15	23826	28209*	23684	28048*	23695	28059*	JX173081.1-2001	JX173080.1-2001	MF315028-2012	NS	2.89E-10	3.32E-03	1.21E-17	1.26E-14	9.49E-04	1.65E-12
15									AF534906.1-1953								
15									JX173082.1-2003								
15									JX173078.1-2000								
15									JX173083.1-2003								
15									JX173085.1-2003								
15									JX173086.1-2004								
16	16	3102*	7573	3046*	7512	3067*	7533	^KX384959.1-2002	KF951595.1-2013	JX173079.1-2002	9.56E-41	3.71E-26	2.69E-25	2.11E-09	3.40E-10	5.23E-08	1.13E-16
16									NC_001405.1-1953								

Supplementary Table S13: list of 117 recombination events identifed by RDP4 analysis package

		Breakpoint Positions															
		In Alignment		In Recombinant Sequence		Relative to NC_001405.1-1953					Detection Methods						
Recombination Event Number	Number In .RDP File	Begin	End	Begin	End	Begin	End	Recombinant Sequence(s)	Minor Parental Sequence(s)	Major Parental Sequence(s)	RDP	GENECONV	Bootscan	Maxchi	Chimaera	SiSscan	3Seq
16									JX173077.1-2005								
16									KF268310.1-1992								
16									KF268130.1-2004								
16									FJ349096.1-1953								
16									LC068717.1-2004								
16									LC068720.1-2005								
16									LC068718.1-2004								
16									LC068713.1-1987								
16									LC068714.1-1993								
16									LC068715.1-1994								
16									LC068716.1-2003								
17	17	13878	18386	13816	18294	13801	18289	^KF268199.1-2008	AF534906.1-1953	AC_000008.1-1953	1.30E-39	8.29E-23	9.77E-21	8.41E-08	1.85E-08	8.05E-07	2.75E-12
17									JX173082.1-2003								
17									JX173078.1-2000								
17									JX173083.1-2003								
17									JX173085.1-2003								
17									JX173086.1-2004								
17									JX173080.1-2001								
18	18	33273	34984	32834	34538	32764	34473	^JX173083.1-2003	JX173081.1-2001	JX173080.1-2001	2.40E-39	9.91E-30	1.87E-39	2.93E-09	2.15E-09	2.27E-10	1.65E-12
18								AF534906.1-1953	KR699642.1-2009								
18								JX173082.1-2003	MF315029-2013								
18								JX173078.1-2000									
18								JX173085.1-2003									
18								JX173086.1-2004									
18								AC_000008.1-1953									
18								KF268199.1-2008[P]									
19	19	22238	24439	22107	24306	22109	24308	^KR699642.1-2009	JX173086.1-2004	KF268310.1-1992	1.46E-38	3.03E-22	1.61E-03	5.52E-07	8.96E-08	1.11E-04	NS
19								MF315029-2013	AF534906.1-1953								
19									JX173082.1-2003								
19									JX173078.1-2000								

Supplementary Table S13: list of 117 recombination events identifed by RDP4 analysis package

		Breakpoint Positions									Detection Methods						
		In Alignment		In Recombinant Sequence		Relative to NC_001405.1-1953											
Recombination Event Number	Number In .RDP File	Begin	End	Begin	End	Begin	End	Recombinant Sequence(s)	Minor Parental Sequence(s)	Major Parental Sequence(s)	RDP	GENECONV	Bootscan	Maxchi	Chimaera	SiSscan	3Seq
19									JX173083.1-2003								
19									JX173085.1-2003								
19									JX173080.1-2001								
20	20	292*	4191*	292*	4160*	291*	4151*	MF315029-2013	Unknown (LC068717.1-2004)	KR699642.1-2009	4.27E-36	1.38E-31	1.32E-37	2.40E-10	3.96E-09	1.54E-07	1.10E-12
20									Unknown(JX173077.1-2005)	JX173079.1-2002							
20									Unknown(JX173079.1-2002)								
20									Unknown(FJ349096.1-1953)								
20									Unknown(KF951595.1-2013)								
20									Unknown(LC068720.1-2005)								
20									Unknown(LC068718.1-2004)								
20									Unknown(LC068713.1-1987)								
20									Unknown(LC068714.1-1993)								
20									Unknown(LC068715.1-1994)								
20									Unknown(LC068716.1-2003)								
21	21	172*	4544*	172*	4529*	171*	4504*	KF268129.1-2005	Unknown (KR699642.1-2009)	LC068716.1-2003	3.06E-32	3.21E-28	8.74E-19	1.13E-11	2.32E-10	1.44E-05	1.10E-12
21									Unknown(JX173079.1-2002)	KF951595.1-2013							
21									Unknown(KX384959.1-2002)	LC068717.1-2004							
21										LC068720.1-2005							
21										LC068718.1-2004							
21										LC068713.1-1987							
21										LC068714.1-1993							
21										LC068715.1-1994							
22	22	6914	14024	6894	13964	6874	13947	^JX173083.1-2003	JX173079.1-2002	JX173082.1-2003	7.73E-31	4.06E-17	3.92E-20	2.38E-05	5.14E-06	3.41E-06	2.20E-12
22								JX173085.1-2003									
22								JX173086.1-2004									
22								MF315028-2012									
22								MF315029-2013									
23	23~	28287	29129	28096	28857	28137	28931	^AC_000008.1-1953	Unknown (KF268129.1-2005)	HQ003817.1-2001	2.27E-24	1.09E-29	3.72E-16	9.70E-08	8.39E-13	9.30E-23	1.29E-05
23								KF268199.1-2008		KR699642.1-2009							

Supplementary Table S13: list of 117 recombination events identifed by RDP4 analysis package

Recombination Event Number	Number In .RDP File	Breakpoint Positions: In Alignment Begin	In Alignment End	In Recombinant Sequence Begin	In Recombinant Sequence End	Relative to NC_001405.1-1953 Begin	Relative to NC_001405.1-1953 End	Recombinant Sequence(s)	Minor Parental Sequence(s)	Major Parental Sequence(s)	Detection Methods: RDP	GENECONV	Bootscan	Maxchi	Chimaera	SiSscan	3Seq
23										JX173079.1-2002							
23										KX384959.1-2002							
23										JX173081.1-2001							
23										MF315029-2013							
24	24	3198	6875*	3165	6837*	3163	6835*	^JX173079.1-2002	AF534906.1-1953	Unknown (JX173083.1-2003)	2.25E-28	4.61E-17	1.94E-28	1.18E-04	9.24E-05	8.19E-23	3.85E-12
24										Unknown(JX173082.1-2003)							
24										Unknown(JX173085.1-2003)							
24										Unknown(JX173086.1-2004)							
25	25	160*	9912	149*	9839	159*	9848	^JX173084.1-2003	Unknown (KF268310.1-1992)	LC068716.1-2003	9.12E-15	4.79E-13	2.08E-06	1.00E-14	8.40E-16	1.88E-07	6.01E-28
25									Unknown(NC_001405.1-1953)	LC068718.1-2004							
25									Unknown(JX173077.1-2005)	LC068713.1-1987							
25									Unknown(KF268130.1-2004)								
25									Unknown(FJ349096.1-1953)								
25									Unknown(AF534906.1-1953)								
25									Unknown(JX173082.1-2003)								
26	26	10085	11840	10009	11760	10021	11763	^LC068716.1-2003	Unknown (JX173078.1-2000)	LC068714.1-1993	2.51E-27	9.55E-22	5.95E-23	1.64E-06	1.52E-05	1.81E-03	1.65E-12
26									Unknown(HQ003817.1-2001)	FJ349096.1-1953							
26									Unknown(AF534906.1-1953)	KF951595.1-2013							
26									Unknown(JX173082.1-2003)	LC068717.1-2004							
26									Unknown(JX173080.1-2001)	LC068720.1-2005							
26										LC068718.1-2004							
26										LC068713.1-1987							
26										LC068715.1-1994							
27	27	952*	10084*	951*	10038*	950*	10020*	^HQ003817.1-2001	Unknown (JX173077.1-2005)	LC068716.1-2003	4.00E-08	3.18E-08	0.019766271	2.19E-09	3.13E-06	2.40E-13	5.03E-37
27									Unknown(NC_001405.1-1953)	KF951595.1-2013							
27									Unknown(KF268310.1-1992)	LC068717.1-2004							
27									Unknown(KF268130.1-2004)	LC068718.1-2004							
27									Unknown(LC068720.1-2005)	LC068713.1-1987							
27										LC068714.1-1993							

Supplementary Table S13: list of 117 recombination events identifed by RDP4 analysis package

Recombination Event Number	Number In .RDP File	Breakpoint Positions: In Alignment Begin	In Alignment End	In Recombinant Sequence Begin	In Recombinant Sequence End	Relative to NC_001405.1-1953 Begin	Relative to NC_001405.1-1953 End	Recombinant Sequence(s)	Minor Parental Sequence(s)	Major Parental Sequence(s)	Detection Methods: RDP	GENECONV	Bootscan	Maxchi	Chimaera	SiSscan	3Seq
27										LC068715.1-1994							
28	28	18794*	24980	18685*	24839	18697*	24849	^JX173079.1-2002	JX173082.1-2003	NC_001405.1-1953	4.50E-19	5.28E-03	4.14E-04	8.13E-05	2.20E-05	1.44E-48	2.20E-12
28								KX384959.1-2002	AF534906.1-1953								
28									JX173078.1-2000								
28									JX173083.1-2003								
28									JX173085.1-2003								
28									JX173086.1-2004								
28									JX173080.1-2001								
29	29	35292*	36429*	34541*	35647*	34781*	35890*	^LC068713.1-1987	Unknown (AF534906.1-1953)	KF951595.1-2013	1.97E-20	2.31E-24	5.52E-22	2.93E-04	3.67E-03	1.75E-03	2.23E-09
29								LC068718.1-2004	Unknown(JX173082.1-2003)	NC_001405.1-1953							
29								LC068714.1-1993	Unknown(JX173078.1-2000)	JX173077.1-2005							
29								LC068715.1-1994	Unknown(JX173083.1-2003)	KF268310.1-1992							
29								LC068716.1-2003	Unknown(JX173085.1-2003)	KF268130.1-2004							
29									Unknown(JX173086.1-2004)	FJ349096.1-1953							
29									Unknown(JX173080.1-2001)	LC068717.1-2004							
29										LC068720.1-2005							
29										HQ003817.1-2001							
30	30	14093	18188	13992	18057	14016	18091	^LC068718.1-2004	AF534906.1-1953	KF951595.1-2013	1.02E-24	1.05E-12	9.87E-14	7.44E-05	1.55E-04	NS	2.61E-09
30									JX173082.1-2003	FJ349096.1-1953							
30									JX173078.1-2000	JX423389.1-2007							
30									JX173083.1-2003	LC068717.1-2004							
30									JX173085.1-2003	LC068713.1-1987							
30									JX173086.1-2004	LC068714.1-1993							
30									JX173080.1-2001	LC068715.1-1994							
30										LC068716.1-2003							
31	31	5107	11704	5077	11637	5067	11627	^AC_000008.1-1953	Unknown (JX173078.1-2000)	KF268199.1-2008	1.02E-23	NS	NS	2.53E-05	1.43E-06	NS	2.20E-12
31									Unknown(AF534906.1-1953)								
31									Unknown(JX173082.1-2003)								

Supplementary Table S13: list of 117 recombination events identifed by RDP4 analysis package

		Breakpoint Positions									Detection Methods						
		In Alignment		In Recombinant Sequence		Relative to NC_001405.1-1953											
Recombination Event Number	Number In .RDP File	Begin	End	Begin	End	Begin	End	Recombinant Sequence(s)	Minor Parental Sequence(s)	Major Parental Sequence(s)	RDP	GENECONV	Bootscan	Maxchi	Chimaera	SiSscan	3Seq
32	32	18461*	19296	18366*	19201	18364*	19197	^AC_000008.1-1953	LC068714.1-1993	Unknown (KF268129.1-2005)	5.47E-21	9.04E-13	4.26E-20	4.29E-04	1.75E-04	NS	1.44E-10
32									FJ349096.1-1953								
32									JX423389.1-2007								
32									KF951595.1-2013								
32									LC068717.1-2004								
32									LC068720.1-2005								
32									LC068718.1-2004								
32									LC068713.1-1987								
32									LC068715.1-1994								
32									LC068716.1-2003								
33	33~	28305	29097	28171	28922	28155	28899	^KF268129.1-2005	KX384959.1-2002	Unknown (AF534906.1-1953)	1.99E-19	NS	1.27E-06	9.75E-09	3.50E-11	6.15E-18	1.29E-05
33									KR699642.1-2009	Unknown(JX173082.1-2003)							
33									JX173079.1-2002	Unknown(JX173078.1-2000)							
33									JX173081.1-2001	Unknown(JX173083.1-2003)							
33									MF315029-2013	Unknown(JX173085.1-2003)							
33										Unknown(JX173086.1-2004)							
34	34	18516*	19296	18452*	19232	18419*	19197	^KF268129.1-2005	Unknown (KF268199.1-2008)	LC068716.1-2003	6.65E-19	1.48E-08	4.97E-19	7.73E-04	2.94E-04	NS	3.23E-07
34										FJ349096.1-1953							
34										JX423389.1-2007							
34										KF951595.1-2013							
34										LC068717.1-2004							
34										LC068720.1-2005							
34										LC068718.1-2004							
34										LC068713.1-1987							
34										LC068714.1-1993							
34										LC068715.1-1994							
35	35	33832	34818*	33139	34125*	33322	34308*	^FJ349096.1-1953	Unknown (JX173080.1-2001)	LC068715.1-1994	2.02E-18	1.59E-17	9.24E-19	3.44E-03	2.50E-04	1.20E-02	3.54E-08
35								NC_001405.1-1953		JX423389.1-2007							
35								JX173077.1-2005[P]		KF951595.1-2013							
35								KF268310.1-1992		LC068717.1-2004							

Supplementary Table S13: list of 117 recombination events identifed by RDP4 analysis package

		Breakpoint Positions									Detection Methods						
		In Alignment		In Recombinant Sequence		Relative to NC_001405.1-1953											
Recombination Event Number	Number In .RDP File	Begin	End	Begin	End	Begin	End	Recombinant Sequence(s)	Minor Parental Sequence(s)	Major Parental Sequence(s)	RDP	GENECONV	Bootscan	Maxchi	Chimaera	SiSscan	3Seq
35								KF268130.1-2004		LC068720.1-2005							
35								JX173084.1-2003		LC068714.1-1993							
35								JX173079.1-2002		HQ003817.1-2001							
35								KX384959.1-2002									
36	36	35482*	36477*	35014*	35979*	34970*	35937*	^KF268129.1-2005	Unknown (KF268199.1-2008)	KF951595.1-2013	1.59E-14	3.60E-07	9.58E-18	1.12E-02	2.90E-03	NS	NS
36									Unknown(JX173083.1-2003)	FJ349096.1-1953							
36									Unknown(JX173085.1-2003)	JX423389.1-2007							
36									Unknown(JX173086.1-2004)	LC068717.1-2004							
36									Unknown(JX173080.1-2001)	LC068720.1-2005							
37	37	20280	20974	20155	20837	20154	20845	^HQ003817.1-2001	KX384959.1-2002	JX173085.1-2003	1.43E-10	5.59E-11	1.81E-08	3.58E-02	4.06E-03	6.64E-17	5.51E-13
37									KR699642.1-2009	AF534906.1-1953							
37									JX173079.1-2002	JX173082.1-2003							
37									JX173081.1-2001	JX173078.1-2000							
37									MF315029-2013	JX173083.1-2003							
37										JX173086.1-2004							
37										JX173080.1-2001							
38	38	7634*	12214	7573*	12119	7594*	12137	^KX384959.1-2002	Unknown (JX173078.1-2000)	JX173079.1-2002	8.00E-16	5.33E-03	6.00E-05	4.97E-05	NS	NS	2.06E-02
38									Unknown(AF534906.1-1953)								
38									Unknown(JX173082.1-2003)								
39	39~	5437	6337	5417	6317	5397	6297	JX173083.1-2003	FJ349096.1-1953	JX173085.1-2003	NS	1.52E-12	1.00E-15	NS	NS	NS	1.07E-03
39									NC_001405.1-1953	JX173082.1-2003							
39									JX173077.1-2005	JX173086.1-2004							
39									KF268310.1-1992								
39									KF268130.1-2004								
39									KF951595.1-2013								
39									LC068717.1-2004								
39									LC068720.1-2005								
39									LC068718.1-2004								
39									LC068713.1-1987								

Supplementary Table S13: list of 117 recombination events identifed by RDP4 analysis package

Recombination Event Number	Number In .RDP File	Breakpoint Positions: In Alignment Begin	End	In Recombinant Sequence Begin	End	Relative to NC_001405.1-1953 Begin	End	Recombinant Sequence(s)	Minor Parental Sequence(s)	Major Parental Sequence(s)	Detection Methods: RDP	GENECONV	Bootscan	Maxchi	Chimaera	SiSscan	3Seq
39									LC068714.1-1993								
39									LC068715.1-1994								
39									LC068716.1-2003								
40	40~	35071*	36475*	34549*	35926*	34560*	35935*	^KF268199.1-2008	JX173085.1-2003	AC_000008.1-1953	3.66E-13	3.59E-10	1.14E-14	NS	NS	NS	1.65E-12
40									FJ349096.1-1953								
40									KF951595.1-2013								
40									LC068717.1-2004								
40									LC068720.1-2005								
40									HQ003817.1-2001								
40									AF534906.1-1953								
40									JX173082.1-2003								
40									JX173078.1-2000								
40									JX173083.1-2003								
40									JX173086.1-2004								
40									JX173080.1-2001								
41	41~	34210	34909*	33698	34393*	33700	34398*	^JX173080.1-2001	LC068715.1-1994	Unknown (LC068718.1-2004)	NS	2.19E-13	5.05E-14	NS	NS	NS	1.23E-03
41									KF951595.1-2013	Unknown(LC068713.1-1987)							
41									LC068717.1-2004	Unknown(LC068716.1-2003)							
41									LC068720.1-2005								
41									LC068714.1-1993								
41									HQ003817.1-2001								
42	42~	29893	30822	29670	30554	29443	30366	^KF268129.1-2005	JX173079.1-2002	JX173085.1-2003	2.81E-02	NS	NS	5.91E-03	6.49E-03	NS	NS
42									KR699642.1-2009	AF534906.1-1953							
42									KX384959.1-2002	JX173082.1-2003							
42									JX173081.1-2001	JX173078.1-2000							
42									MF315029-2013	JX173083.1-2003							
42										JX173086.1-2004							
43	43	11495	18566*	11419	18461*	11418	18469*	^JX173081.1-2001	Unknown (JX173080.1-2001)	KF268199.1-2008	NS	1.02E-02	4.23E-03	1.36E-05	8.16E-07	NS	NS

Supplementary Table S13: list of 117 recombination events identifed by RDP4 analysis package

		Breakpoint Positions																
		In Alignment		In Recombinant Sequence		Relative to NC_001405.1-1953					Detection Methods							
Recombination Event Number	Number In .RDP File	Begin	End	Begin	End	Begin	End	Recombinant Sequence(s)	Minor Parental Sequence(s)	Major Parental Sequence(s)	RDP	GENECONV	Bootscan	Maxchi	Chimaera	SiSscan	3Seq
44	44	363*	36250*	363*	35701*	362*	35714*	^JX173079.1-2002	FJ349096.1-1953	Unknown (AF534906.1-1953)	1.53E-09	1.20E-10	5.69E-13	2.42E-05	1.12E-05	2.44E-52	NS
44								KX384959.1-2002[P]	NC_001405.1-1953	Unknown(JX173082.1-2003)							
44									JX173077.1-2005	Unknown(JX173080.1-2001)							
44									KF268310.1-1992								
44									KF268130.1-2004								
44									KF951595.1-2013								
44									LC068717.1-2004								
44									LC068720.1-2005								
44									LC068718.1-2004								
44									LC068713.1-1987								
44									LC068714.1-1993								
44									LC068715.1-1994								
44									LC068716.1-2003								
45	45~	703*	2095*	685*	2067*	701*	2081*	^JX173078.1-2000	AC_000008.1-1953	AF534906.1-1953	8.21E-13	NS	2.39E-11	NS	NS	NS	2.90E-02
45										JX173080.1-2001							
46	46~	20060	20522	19902	20349	19934	20393	^AC_000008.1-1953	Unknown (JX173081.1-2001)	JX173083.1-2003	4.85E-04	NS	1.15E-03	NS	NS	9.42E-13	NS
46								KF268199.1-2008	Unknown(KR699642.1-2009)	AF534906.1-1953							
46									Unknown(JX173079.1-2002)	JX173082.1-2003							
46									Unknown(KX384959.1-2002)	JX173078.1-2000							
46									Unknown(MF315029-2013)	JX173085.1-2003							
46										JX173086.1-2004							
46										JX173080.1-2001							
47	47	34203	35291*	33572	34656*	33693	34780*	^HQ003817.1-2001	LC068715.1-1994	Unknown (LC068718.1-2004)	4.36E-11	3.18E-10	1.34E-12	NS	NS	NS	1.99E-05
47									JX423389.1-2007	Unknown(LC068713.1-1987)							
47									KF951595.1-2013	Unknown(LC068716.1-2003)							
47									LC068714.1-1993								
48	48	8340	13251	8312	13182	8300	13174	^JX173080.1-2001	JX173079.1-2002	JX173082.1-2003	2.43E-12	NS	9.30E-08	9.76E-03	4.92E-02	NS	0.042174983
48										AF534906.1-1953							

Supplementary Table S13: list of 117 recombination events identifed by RDP4 analysis package

		Breakpoint Positions									Detection Methods						
		In Alignment		In Recombinant Sequence		Relative to NC_001405.1-1953											
Recombination Event Number	Number In .RDP File	Begin	End	Begin	End	Begin	End	Recombinant Sequence(s)	Minor Parental Sequence(s)	Major Parental Sequence(s)	RDP	GENECONV	Bootscan	Maxchi	Chimaera	SiSscan	3Seq
49	49~	7899	11709	7864	11640	7859	11632	^JX173078.1-2000	JX173079.1-2002	JX173082.1-2003	9.17E-14	3.29E-11	1.67E-11	NS	NS	NS	9.10E-06
49								KR699642.1-2009	JX173077.1-2005	AF534906.1-1953							
49									FJ349096.1-1953								
49									LC068718.1-2004								
49									LC068713.1-1987								
49									LC068714.1-1993								
49									LC068715.1-1994								
50	50	8234	19257*	8191	19157*	8194	19158*	KR699642.1-2009	AF534906.1-1953	LC068717.1-2004	1.23E-05	2.28E-04	NS	1.82E-10	2.16E-08	1.78E-08	NS
50									JX173082.1-2003	FJ349096.1-1953							
50									JX173078.1-2000	KF951595.1-2013							
50										LC068720.1-2005							
50										LC068718.1-2004							
50										LC068713.1-1987							
50										LC068714.1-1993							
50										LC068715.1-1994							
50										LC068716.1-2003							
51	51	24849	25997	24745	25893	24718	25866	^MF315029-2013	JX173083.1-2003	Unknown (AC_000008.1-1953)	1.77E-06	1.15E-10	4.26E-11	3.12E-06	1.10E-03	1.04E-12	NS
51								NC_001405.1-1953	AF534906.1-1953	Unknown(KF268199.1-2008)							
51								JX173077.1-2005	JX173082.1-2003								
51								KF268310.1-1992	JX173078.1-2000								
51								KF268130.1-2004	JX173085.1-2003								
51								JX173084.1-2003	JX173086.1-2004								
51								KR699642.1-2009	JX173080.1-2001								
51								FJ349096.1-1953									
51								MF315028-2012									
52	52~	2875*	4428*	2844*	4393*	2840*	4388*	^JX173078.1-2000	KR699642.1-2009	JX173082.1-2003	7.08E-11	NS	2.46E-02	2.00E-04	NS	NS	NS
52									FJ349096.1-1953								
52									KF951595.1-2013								
52									LC068717.1-2004								
52									LC068720.1-2005								

Supplementary Table S13: list of 117 recombination events identifed by RDP4 analysis package

		Breakpoint Positions									Detection Methods						
		In Alignment		In Recombinant Sequence		Relative to NC_001405.1-1953											
Recombination Event Number	Number In .RDP File	Begin	End	Begin	End	Begin	End	Recombinant Sequence(s)	Minor Parental Sequence(s)	Major Parental Sequence(s)	RDP	GENECONV	Bootscan	Maxchi	Chimaera	SiSscan	3Seq
52									LC068718.1-2004								
52									LC068713.1-1987								
52									LC068714.1-1993								
52									LC068715.1-1994								
52									LC068716.1-2003								
53	53~	6864	7805*	6842	7783*	6824	7765*	^KF268199.1-2008	JX173078.1-2000	Unknown (JX173082.1-2003)	1.58E-11	2.85E-05	6.62E-07	NS	NS	NS	NS
53										Unknown(AF534906.1-1953)							
54	54	587	4191	583	4148	585	4151	^KR699642.1-2009	LC068717.1-2004	Unknown (KF268199.1-2008)	NS	5.21E-08	8.97E-11	1.68E-04	NS	7.49E-11	NS
54									FJ349096.1-1953								
54									KF951595.1-2013								
54									LC068720.1-2005								
54									LC068718.1-2004								
54									LC068713.1-1987								
54									LC068714.1-1993								
54									LC068715.1-1994								
54									LC068716.1-2003								
55	55~	35452*	36277*	34903*	35697*	34940*	35741*	^KX384959.1-2002	AC_000008.1-1953	JX173081.1-2001	2.94E-10	1.51E-03	3.83E-11	3.37E-03	NS	NS	1.53E-05
55								JX173079.1-2002		KR699642.1-2009							
55										MF315029-2013							
56	56~	35439*	35451*	34911*	34922*	34928*	34939*	^KR699642.1-2009	JX173082.1-2003	KX384959.1-2002	NS	NS	NS	NS	NS	NS	1.65E-12
56								MF315029-2013	AF534906.1-1953								
56									JX173078.1-2000								
57	57	19811	20279	19692	20154	19685	20153	HQ003817.1-2001	JX173079.1-2002	LC068715.1-1994	1.57E-04	NS	2.50E-03	3.90E-02	1.99E-03	4.49E-11	1.63E-03
57									KX384959.1-2002	FJ349096.1-1953							
57									JX173081.1-2001	JX423389.1-2007							
57										KF951595.1-2013							
57										LC068717.1-2004							
57										LC068720.1-2005							

Supplementary Table S13: list of 117 recombination events identifed by RDP4 analysis package

		Breakpoint Positions									Detection Methods						
		In Alignment		In Recombinant Sequence		Relative to NC_001405.1-1953											
Recombination Event Number	Number In .RDP File	Begin	End	Begin	End	Begin	End	Recombinant Sequence(s)	Minor Parental Sequence(s)	Major Parental Sequence(s)	RDP	GENECONV	Bootscan	Maxchi	Chimaera	SiSscan	3Seq
57										LC068718.1-2004							
57										LC068713.1-1987							
57										LC068714.1-1993							
57										LC068716.1-2003							
57										KF268129.1-2005							
58	58	4220	6716*	4192	6688*	4180	6676*	JX173080.1-2001	Unknown (LC068713.1-1987)	JX173085.1-2003	1.64E-09	NS	NS	NS	NS	NS	NS
58									Unknown(KF951595.1-2013)	JX173082.1-2003							
58									Unknown(LC068717.1-2004)	JX173086.1-2004							
58									Unknown(LC068720.1-2005)								
59	59	11862	18270*	11787	18168*	11785	18173*	^KF268130.1-2004	Unknown (AF534906.1-1953)	KF951595.1-2013	1.83E-04	NS	3.58E-05	2.21E-09	5.62E-06	NS	1.07E-05
59								NC_001405.1-1953	Unknown(JX173082.1-2003)	FJ349096.1-1953							
59								KF268310.1-1992	Unknown(JX173078.1-2000)	LC068717.1-2004							
59								LC068720.1-2005[P]		LC068720.1-2005							
59										LC068713.1-1987							
59										LC068714.1-1993							
59										LC068715.1-1994							
59										LC068716.1-2003							
60	60~	36*	597*	36*	596*	35*	595*	JX423389.1-2007	JX173083.1-2003	LC068716.1-2003	NS	9.65E-06	5.28E-09	NS	NS	NS	NS
60									AF534906.1-1953	LC068718.1-2004							
60									JX173082.1-2003	LC068714.1-1993							
60									JX173085.1-2003	LC068715.1-1994							
60									JX173086.1-2004								
60									JX173080.1-2001								
60									AC_000008.1-1953								
61	61~	19324	19688	19229	19542	19225	19571	^AC_000008.1-1953	Unknown (KF268129.1-2005)	JX173080.1-2001	NS	NS	NS	NS	NS	5.88E-09	NS
61									Unknown(FJ349096.1-1953)	AF534906.1-1953							
61									Unknown(JX423389.1-2007)	JX173082.1-2003							
61									Unknown(KF951595.1-2013)	JX173078.1-2000							
61									Unknown(LC068717.1-2004)	JX173083.1-2003							

Supplementary Table S13: list of 117 recombination events identifed by RDP4 analysis package

		Breakpoint Positions															
		In Alignment		In Recombinant Sequence		Relative to NC_001405.1-1953					Detection Methods						
Recombination Event Number	Number In .RDP File	Begin	End	Begin	End	Begin	End	Recombinant Sequence(s)	Minor Parental Sequence(s)	Major Parental Sequence(s)	RDP	GENECONV	Bootscan	Maxchi	Chimaera	SiSscan	3Seq
61									Unknown(LC068720.1-2005)	JX173085.1-2003							
61									Unknown(LC068718.1-2004)	JX173086.1-2004							
61									Unknown(LC068713.1-1987)								
61									Unknown(LC068714.1-1993)								
61									Unknown(LC068715.1-1994)								
61									Unknown(LC068716.1-2003)								
62	62	2928*	35268*	2895*	34741*	2893*	34757*	JX173079.1-2002	JX173082.1-2003	Unknown (LC068717.1-2004)	1.07E-02	NS	3.40E-04	1.55E-08	8.21E-06	1.42E-52	4.32E-07
62								JX173077.1-2005	AF534906.1-1953	Unknown(FJ349096.1-1953)							
62								JX173084.1-2003[P]	JX173078.1-2000	Unknown(KF951595.1-2013)							
62								JX173083.1-2003[T]		Unknown(LC068713.1-1987)							
62								JX173085.1-2003[T]		Unknown(LC068714.1-1993)							
62								JX173086.1-2004[T]		Unknown(LC068715.1-1994)							
62								KF268199.1-2008									
62								MF315028-2012[T]									
62								MF315029-2013[T]									
63	63~	27476*	29605	27318*	29336	27327*	29300	^HQ003817.1-2001	Unknown (JX173079.1-2002)	FJ349096.1-1953	NS	2.99E-02	5.04E-04	4.10E-04	NS	2.48E-14	NS
63									Unknown(KR699642.1-2009)								
63									Unknown(KX384959.1-2002)								
63									Unknown(JX173081.1-2001)								
63									Unknown(MF315029-2013)								
64	66~	22572	26738	22397	26559	22441	26598	^KF268199.1-2008	KF268130.1-2004	JX173080.1-2001	2.56E-06	NS	NS	2.35E-02	NS	6.82E-09	NS
64								AC_000008.1-1953	JX173079.1-2002	AF534906.1-1953							
64									KX384959.1-2002	JX173082.1-2003							
64										JX173078.1-2000							
64										JX173083.1-2003							
64										JX173085.1-2003							
64										JX173086.1-2004							
65	67~	150*	586*	133*	568*	149*	584*	^JX173078.1-2000	Unknown (KR699642.1-2009)	JX173082.1-2003	NS	2.51E-07	2.87E-07	NS	NS	NS	NS
65									Unknown(MF315029-2013)	AF534906.1-1953							

Supplementary Table S13: list of 117 recombination events identifed by RDP4 analysis package

Recombination Event Number	Number In .RDP File	Breakpoint Positions: In Alignment Begin	In Alignment End	In Recombinant Sequence Begin	In Recombinant Sequence End	Relative to NC_001405.1-1953 Begin	Relative to NC_001405.1-1953 End	Recombinant Sequence(s)	Minor Parental Sequence(s)	Major Parental Sequence(s)	Detection Methods: RDP	GENECONV	Bootscan	Maxchi	Chimaera	SiSscan	3Seq
65										JX173083.1-2003							
65										JX173085.1-2003							
65										JX173086.1-2004							
66	68~	23069	23664	22847	23442	22938	23533	^LC068720.1-2005	KF268310.1-1992	JX173085.1-2003	1.98E-03	3.15E-07	NS	2.31E-04	NS	NS	NS
66								JX423389.1-2007	JX173081.1-2001	AF534906.1-1953							
66								KF951595.1-2013		JX173082.1-2003							
66								LC068717.1-2004		JX173078.1-2000							
66								LC068718.1-2004		JX173083.1-2003							
66								LC068713.1-1987		JX173086.1-2004							
66								LC068714.1-1993		JX173080.1-2001							
66								LC068715.1-1994									
66								LC068716.1-2003									
67	69~	20207	20449	20085	20321	20081	20320	^KF951595.1-2013	JX173081.1-2001	JX173085.1-2003	NS	NS	NS	NS	NS	4.73E-07	NS
67								JX423389.1-2007	KR699642.1-2009	JX173078.1-2000							
67								LC068717.1-2004	JX173079.1-2002	JX173086.1-2004							
67								LC068720.1-2005	KX384959.1-2002	JX173080.1-2001							
67								LC068718.1-2004	MF315029-2013								
67								LC068713.1-1987									
67								LC068714.1-1993									
67								LC068715.1-1994									
67								LC068716.1-2003									
67								KF268129.1-2005									
68	71~	20207	20449	20072	20308	20081	20320	^FJ349096.1-1953	JX173081.1-2001	JX173078.1-2000	NS	NS	NS	NS	NS	4.73E-07	NS
68									JX173079.1-2002	JX173080.1-2001							
68									KX384959.1-2002								
68									MF315029-2013								
69	72~	1*	4544*	1*	4513*	1*	4504*	^MF315029-2013	AC_000008.1-1953	Unknown (KF268199.1-2008)	NS	1.66E-09	7.34E-07	NS	NS	NS	NS
70	73~	26127*	26570	25970*	26410	25996*	26430	^KX384959.1-2002	LC068714.1-1993	MF315029-2013	NS	2.61E-06	1.02E-02	NS	NS	NS	NS

Supplementary Table S13: list of 117 recombination events identifed by RDP4 analysis package

		Breakpoint Positions									Detection Methods						
		In Alignment		In Recombinant Sequence		Relative to NC_001405.1-1953											
Recombination Event Number	Number In .RDP File	Begin	End	Begin	End	Begin	End	Recombinant Sequence(s)	Minor Parental Sequence(s)	Major Parental Sequence(s)	RDP	GENECONV	Bootscan	Maxchi	Chimaera	SiSscan	3Seq
70								JX173079.1-2002	JX423389.1-2007	KR699642.1-2009							
70									KF951595.1-2013								
70									LC068717.1-2004								
70									LC068720.1-2005								
70									LC068718.1-2004								
70									LC068713.1-1987								
70									LC068715.1-1994								
70									LC068716.1-2003								
70									HQ003817.1-2001								
71	74~	13532	23385*	13473	23245*	13455	23254*	^HQ003817.1-2001	Unknown (KX384959.1-2002)	JX173078.1-2000	NS	NS	NS	4.88E-07	5.99E-03	5.33E-25	NS
71									Unknown(JX173079.1-2002)	AF534906.1-1953							
71										JX173082.1-2003							
71										JX173083.1-2003							
71										JX173085.1-2003							
71										JX173086.1-2004							
72	75~	587*	702*	569*	684*	585*	700*	^JX173078.1-2000	Unknown (AC_000008.1-1953)	HQ003817.1-2001	NS	NS	NS	NS	NS	NS	5.34E-08
72								AF534906.1-1953[T]									
72								JX173082.1-2003[T]									
73	76	14540	17587*	14489	17524*	14461	17490*	^KF268129.1-2005	JX173078.1-2000	JX173079.1-2002	NS	NS	NS	NS	2.84E-03	NS	NS
73									HQ003817.1-2001								
73									AF534906.1-1953								
73									JX173082.1-2003								
74	77~	82*	241*	82*	241*	81*	240*	^AC_000008.1-1953	JX173082.1-2003	Unknown (HQ003817.1-2001)	NS	NS	NS	NS	NS	NS	4.53E-08
74									JX173083.1-2003								
74									JX173085.1-2003								
74									JX173086.1-2004								
75	78~	32074	32333	31592	31848	31580	31830	^AC_000008.1-1953	Unknown (KF268129.1-2005)	AF534906.1-1953	3.46E-06	1.37E-02	6.75E-05	2.75E-05	9.31E-03	NS	NS
75								KF268199.1-2008	Unknown(FJ349096.1-1953)	JX173082.1-2003							

Supplementary Table S13: list of 117 recombination events identifed by RDP4 analysis package

		Breakpoint Positions									Detection Methods						
		In Alignment		In Recombinant Sequence		Relative to NC_001405.1-1953											
Recombination Event Number	Number In .RDP File	Begin	End	Begin	End	Begin	End	Recombinant Sequence(s)	Minor Parental Sequence(s)	Major Parental Sequence(s)	RDP	GENECONV	Bootscan	Maxchi	Chimaera	SiSscan	3Seq
75									Unknown(JX423389.1-2007)	JX173078.1-2000							
75									Unknown(KF951595.1-2013)	JX173083.1-2003							
75									Unknown(LC068717.1-2004)	JX173085.1-2003							
75									Unknown(LC068720.1-2005)	JX173086.1-2004							
75									Unknown(LC068718.1-2004)	JX173080.1-2001							
75									Unknown(LC068713.1-1987)								
75									Unknown(LC068714.1-1993)								
75									Unknown(LC068715.1-1994)								
75									Unknown(LC068716.1-2003)								
76	80~	31728*	32034	31246*	31552	31234*	31540	^AC_000008.1-1953	Unknown (KX384959.1-2002)	LC068720.1-2005	NS	NS	NS	NS	NS	9.16E-07	NS
77	81	33467*	33973*	32973*	33479*	32957*	33463*	^MF315028-2012	KF951595.1-2013	Unknown (KF268310.1-1992)	1.99E-02	3.64E-04	3.71E-04	NS	NS	NS	4.06E-06
77									LC068717.1-2004	Unknown(NC_001405.1-1953)							
77									LC068720.1-2005	Unknown(JX173077.1-2005)							
77									LC068714.1-1993	Unknown(KF268130.1-2004)							
77									LC068715.1-1994	Unknown(JX173084.1-2003)							
78	82~	36013	36408*	35516	35890*	35497	35869*	^MF315029-2013	JX173080.1-2001	JX173081.1-2001	NS	5.43E-06	8.96E-03	NS	NS	NS	NS
78								KR699642.1-2009	FJ349096.1-1953								
78									JX423389.1-2007								
78									KF951595.1-2013								
78									AF534906.1-1953								
78									JX173082.1-2003								
78									JX173078.1-2000								
79	83	17081	19227*	16976	19121*	16984	19128*	^JX173081.1-2001	JX423389.1-2007	Unknown (JX173080.1-2001)	NS	NS	NS	NS	2.10E-05	3.56E-06	1.60E-03
79									FJ349096.1-1953	Unknown(AF534906.1-1953)							
79									KF951595.1-2013	Unknown(JX173082.1-2003)							
79									LC068717.1-2004	Unknown(JX173078.1-2000)							
79									LC068713.1-1987	Unknown(JX173083.1-2003)							
79									LC068714.1-1993	Unknown(JX173085.1-2003)							
79									LC068715.1-1994	Unknown(JX173086.1-2004)							

Supplementary Table S13: list of 117 recombination events identifed by RDP4 analysis package

Recombination Event Number	Number In .RDP File	Breakpoint Positions: In Alignment: Begin	In Alignment: End	In Recombinant Sequence: Begin	In Recombinant Sequence: End	Relative to NC_001405.1-1953: Begin	Relative to NC_001405.1-1953: End	Recombinant Sequence(s)	Minor Parental Sequence(s)	Major Parental Sequence(s)	Detection Methods: RDP	GENECONV	Bootscan	Maxchi	Chimaera	SiSscan	3Seq
79									LC068716.1-2003								
79									HQ003817.1-2001								
80	84~	19324	19606	19231	19462	19225	19489	^KF268199.1-2008	Unknown (JX173080.1-2001)	KF951595.1-2013	NS	NS	NS	NS	NS	9.16E-07	NS
80									Unknown(JX173083.1-2003)	FJ349096.1-1953							
80										LC068717.1-2004							
80										LC068720.1-2005							
80										LC068718.1-2004							
80										LC068713.1-1987							
80										LC068714.1-1993							
80										LC068715.1-1994							
80										LC068716.1-2003							
81	85~	19839*	20049	19683*	19893	19713*	19923	^KF268199.1-2008	Unknown (KR699642.1-2009)	JX173078.1-2000	NS	NS	NS	NS	NS	1.05E-06	NS
81										JX173080.1-2001							
82	86~	13469	13877*	13393	13801*	13392	13800*	^JX173081.1-2001	Unknown (KF268310.1-1992)	KF268129.1-2005	2.92E-02	NS	NS	NS	NS	NS	NS
82									Unknown(KX384959.1-2002)								
82									Unknown(FJ349096.1-1953)								
82									Unknown(JX423389.1-2007)								
82									Unknown(KF951595.1-2013)								
82									Unknown(LC068713.1-1987)								
82									Unknown(LC068714.1-1993)								
82									Unknown(LC068715.1-1994)								
82									Unknown(LC068716.1-2003)								
82									Unknown(AC_000008.1-1953)								
83	87~	29699	30064	29311	29613	29310	29611	^AC_000008.1-1953	KR699642.1-2009	JX173085.1-2003	NS	NS	NS	NS	NS	7.66E-06	NS
83								KF268199.1-2008	KX384959.1-2002	AF534906.1-1953							
83										JX173082.1-2003							
83										JX173078.1-2000							
83										JX173083.1-2003							
83										JX173086.1-2004							

Supplementary Table S13: list of 117 recombination events identifed by RDP4 analysis package

		Breakpoint Positions									Detection Methods						
		In Alignment		In Recombinant Sequence		Relative to NC_001405.1-1953											
Recombination Event Number	Number In .RDP File	Begin	End	Begin	End	Begin	End	Recombinant Sequence(s)	Minor Parental Sequence(s)	Major Parental Sequence(s)	RDP	GENECONV	Bootscan	Maxchi	Chimaera	SiSscan	3Seq
84	88~	30644	30878	30185	30419	30188	30410	^AC_000008.1-1953	KR699642.1-2009	AF534906.1-1953	NS	NS	NS	NS	NS	1.03E-05	NS
84								KF268199.1-2008	JX173079.1-2002	JX173082.1-2003							
84									KX384959.1-2002	JX173078.1-2000							
84									JX173081.1-2001	JX173083.1-2003							
84										JX173085.1-2003							
84										JX173086.1-2004							
85	89~	19382	19564	19299	19454	19281	19447	^HQ003817.1-2001	Unknown (JX173079.1-2002)	FJ349096.1-1953	NS	NS	NS	1.13E-05	3.51E-02	NS	NS
85									Unknown(KR699642.1-2009)	JX423389.1-2007							
85									Unknown(KX384959.1-2002)	KF951595.1-2013							
85									Unknown(JX173081.1-2001)	LC068717.1-2004							
85									Unknown(MF315029-2013)	LC068720.1-2005							
85										LC068718.1-2004							
85										LC068713.1-1987							
85										LC068714.1-1993							
85										LC068715.1-1994							
85										LC068716.1-2003							
85										KF268129.1-2005							
86	90~	242*	951*	242*	950*	241*	949*	^AC_000008.1-1953	JX173083.1-2003	Unknown (HQ003817.1-2001)	NS	NS	NS	NS	NS	NS	1.08E-07
86									JX173085.1-2003								
86									JX173086.1-2004								
87	91~	18307*	18824	18198*	18715	18210*	18727	^JX173079.1-2002	JX173078.1-2000	Unknown (HQ003817.1-2001)	9.18E-03	2.42E-05	1.06E-06	4.17E-02	6.92E-03	NS	3.54E-03
87								KX384959.1-2002	AF534906.1-1953								
87									JX173082.1-2003								
87									JX173083.1-2003								
87									JX173085.1-2003								
87									JX173086.1-2004								
87									JX173080.1-2001								
88	93~	19904	20178	19799	20073	19778	20052	^JX173083.1-2003	JX173079.1-2002	LC068720.1-2005	NS	NS	NS	NS	NS	1.85E-05	NS

Supplementary Table S13: list of 117 recombination events identifed by RDP4 analysis package

		Breakpoint Positions									Detection Methods						
		In Alignment		In Recombinant Sequence		Relative to NC_001405.1-1953											
Recombination Event Number	Number In .RDP File	Begin	End	Begin	End	Begin	End	Recombinant Sequence(s)	Minor Parental Sequence(s)	Major Parental Sequence(s)	RDP	GENECONV	Bootscan	Maxchi	Chimaera	SiSscan	3Seq
88								JX173085.1-2003	KR699642.1-2009	FJ349096.1-1953							
88								JX173086.1-2004		KF951595.1-2013							
88								JX173080.1-2001		LC068717.1-2004							
88										LC068718.1-2004							
88										LC068713.1-1987							
88										LC068714.1-1993							
88										LC068715.1-1994							
88										LC068716.1-2003							
89	94~	7392*	63	7354*	63	7352*	62	JX173079.1-2002	Unknown (LC068720.1-2005)	JX173078.1-2000	NS	NS	NS	NS	NS	3.15E-25	NS
89									Unknown(FJ349096.1-1953)	JX173080.1-2001							
89									Unknown(KF951595.1-2013)								
89									Unknown(LC068717.1-2004)								
89									Unknown(LC068718.1-2004)								
89									Unknown(LC068713.1-1987)								
89									Unknown(LC068714.1-1993)								
89									Unknown(LC068715.1-1994)								
89									Unknown(LC068716.1-2003)								
90	95~	19800	19903*	19648	19745*	19677	19777*	AC_000008.1-1953	Unknown (KF268129.1-2005)	JX173085.1-2003	NS	NS	NS	NS	NS	5.06E-08	NS
90										JX173083.1-2003							
90										JX173086.1-2004							
90										JX173080.1-2001							
91	96~	17702	19799*	17624	19700*	17605	19676*	^JX173083.1-2003	Unknown (HQ003817.1-2001)	AC_000008.1-1953	1.74E-04	1.66E-05	NS	7.76E-06	2.75E-03	6.53E-03	3.25E-04
91								AF534906.1-1953									
91								JX173082.1-2003									
91								JX173078.1-2000									
91								JX173085.1-2003									
91								JX173086.1-2004									
91								JX173080.1-2001									
92	97~	13587*	13861*	13511*	13785*	13510*	13784*	^JX173081.1-2001	KF268129.1-2005	AF534906.1-1953	3.66E-02	3.39E-02	NS	NS	NS	NS	NS

Supplementary Table S13: list of 117 recombination events identifed by RDP4 analysis package

		Breakpoint Positions									Detection Methods						
		In Alignment		In Recombinant Sequence		Relative to NC_001405.1-1953											
Recombination Event Number	Number In .RDP File	Begin	End	Begin	End	Begin	End	Recombinant Sequence(s)	Minor Parental Sequence(s)	Major Parental Sequence(s)	RDP	GENECONV	Bootscan	Maxchi	Chimaera	SiSscan	3Seq
92										JX173082.1-2003							
92										JX173078.1-2000							
93	98~	6912*	8339*	6876*	8303*	6872*	8299*	^JX173081.1-2001	JX173080.1-2001	Unknown (AF534906.1-1953)	NS	NS	NS	1.21E-02	1.11E-02	NS	1.92E-03
93										Unknown(JX173082.1-2003)							
94	99~	34985*	35315*	34539*	34869*	34474*	34804*	^JX173083.1-2003	MF315029-2013	AF534906.1-1953	NS	2.14E-03	3.84E-04	NS	NS	NS	NS
94								JX173085.1-2003	KR699642.1-2009								
94								JX173086.1-2004									
94								JX173080.1-2001									
95	100~	35638*	36422*	35142*	35899*	35126*	35883*	^MF315028-2012	JX173082.1-2003	Unknown (NC_001405.1-1953)	2.08E-03	4.78E-04	3.92E-04	NS	4.27E-02	NS	1.32E-03
95									AF534906.1-1953	Unknown(JX173077.1-2005)							
95										Unknown(KF268310.1-1992)							
95										Unknown(KF268130.1-2004)							
95										Unknown(FJ349096.1-1953)							
95										Unknown(KF951595.1-2013)							
95										Unknown(LC068720.1-2005)							
96	101~	31867	32291	31362	31786	31373	31797	^JX173081.1-2001	Unknown (KF268129.1-2005)	JX173082.1-2003	2.32E-04	NS	5.77E-03	7.24E-05	5.09E-04	NS	NS
96								NC_001405.1-1953	Unknown(FJ349096.1-1953)	AF534906.1-1953							
96								JX173077.1-2005	Unknown(JX423389.1-2007)	JX173078.1-2000							
96								KF268310.1-1992	Unknown(KF951595.1-2013)	JX173083.1-2003							
96								KF268130.1-2004	Unknown(LC068717.1-2004)	JX173085.1-2003							
96								JX173084.1-2003	Unknown(LC068720.1-2005)	JX173086.1-2004							
96								KR699642.1-2009	Unknown(LC068718.1-2004)	JX173080.1-2001							
96								JX173079.1-2002	Unknown(LC068713.1-1987)								
96								KX384959.1-2002	Unknown(LC068714.1-1993)								
96								MF315028-2012	Unknown(LC068715.1-1994)								
96								MF315029-2013	Unknown(LC068716.1-2003)								
97	103	1281*	6012*	1270*	5992*	1269*	5972*	^JX173082.1-2003	JX173085.1-2003	Unknown (AF534906.1-1953)	1.54E-03	NS	NS	2.92E-07	1.52E-06	NS	5.37E-09
97									JX173083.1-2003								

Supplementary Table S13: list of 117 recombination events identifed by RDP4 analysis package

		Breakpoint Positions									Detection Methods						
		In Alignment		In Recombinant Sequence		Relative to NC_001405.1-1953											
Recombination Event Number	Number In .RDP File	Begin	End	Begin	End	Begin	End	Recombinant Sequence(s)	Minor Parental Sequence(s)	Major Parental Sequence(s)	RDP	GENECONV	Bootscan	Maxchi	Chimaera	SiSscan	3Seq
97									JX173086.1-2004								
98	104~	32515	32720	32086	32285	32009	32214	^AF534906.1-1953	Unknown (MF315029-2013)	JX423389.1-2007	NS	NS	NS	NS	NS	8.62E-07	NS
98								JX173078.1-2000	Unknown(KR699642.1-2009)	FJ349096.1-1953							
98								JX173080.1-2001		KF951595.1-2013							
98										LC068717.1-2004							
98										LC068720.1-2005							
98										LC068718.1-2004							
98										LC068713.1-1987							
98										LC068714.1-1993							
98										LC068715.1-1994							
98										LC068716.1-2003							
99	105~	19310*	36477*	19227*	35775*	19211*	35937*	^KF951595.1-2013	MF315029-2013	JX173083.1-2003	NS	NS	NS	NS	NS	3.99E-37	NS
99								FJ349096.1-1953[P]	KR699642.1-2009	AF534906.1-1953							
99								JX423389.1-2007		JX173082.1-2003							
99								LC068717.1-2004		JX173078.1-2000							
99								LC068720.1-2005		JX173085.1-2003							
99								LC068718.1-2004[P]		JX173086.1-2004							
99								LC068713.1-1987[P]		JX173080.1-2001							
99								LC068714.1-1993[P]									
99								LC068715.1-1994[P]									
99								LC068716.1-2003[P]									
100	106~	32430	32590	31942	32096	31924	32084	AC_000008.1-1953	KR699642.1-2009	JX173082.1-2003	NS	NS	NS	NS	NS	2.77E-06	NS
100								KF268199.1-2008	KX384959.1-2002	JX173083.1-2003							
100										JX173085.1-2003							
100										JX173086.1-2004							
101	107~	19904	20178	19801	20075	19778	20052	^AF534906.1-1953	KX384959.1-2002	KF951595.1-2013	NS	NS	NS	NS	NS	1.85E-05	NS
101									JX173081.1-2001	FJ349096.1-1953							
101										LC068717.1-2004							
101										LC068713.1-1987							

Supplementary Table S13: list of 117 recombination events identifed by RDP4 analysis package

		Breakpoint Positions															
		In Alignment		In Recombinant Sequence		Relative to NC_001405.1-1953					Detection Methods						
Recombination Event Number	Number In .RDP File	Begin	End	Begin	End	Begin	End	Recombinant Sequence(s)	Minor Parental Sequence(s)	Major Parental Sequence(s)	RDP	GENECONV	Bootscan	Maxchi	Chimaera	SiSscan	3Seq
101										LC068714.1-1993							
101										LC068715.1-1994							
101										LC068716.1-2003							
101										KF268129.1-2005							
102	108~	19328	19903*	19243	19791*	19229	19777*	^JX173080.1-2001	Unknown (KR699642.1-2009)	FJ349096.1-1953	NS	NS	NS	NS	NS	1.84E-14	NS
102								JX173082.1-2003	Unknown(MF315029-2013)	JX423389.1-2007							
102								JX173078.1-2000		KF951595.1-2013							
102								JX173083.1-2003		LC068717.1-2004							
102								JX173085.1-2003		LC068720.1-2005							
102								JX173086.1-2004		LC068718.1-2004							
102										LC068713.1-1987							
102										LC068714.1-1993							
102										LC068715.1-1994							
102										LC068716.1-2003							
103	110~	19296	20206	19200	20071	19197	20080	^FJ349096.1-1953	Unknown (KX384959.1-2002)	JX173082.1-2003	NS	NS	NS	NS	NS	1.85E-05	NS
103										JX173078.1-2000							
104	111~	19904	20178	19798	20072	19778	20052	^JX173082.1-2003	KX384959.1-2002	KF951595.1-2013	NS	NS	NS	NS	NS	1.85E-05	NS
104									JX173081.1-2001	LC068717.1-2004							
104										LC068713.1-1987							
104										LC068714.1-1993							
104										LC068715.1-1994							
104										LC068716.1-2003							
105	112~	19325*	19666	19246*	19572	19226*	19549	^JX173082.1-2003	MF315029-2013	JX423389.1-2007	NS	NS	NS	NS	NS	1.05E-04	NS
105								AF534906.1-1953	KR699642.1-2009	KF951595.1-2013							
105								JX173078.1-2000		LC068717.1-2004							
105								JX173083.1-2003		LC068720.1-2005							
105								JX173085.1-2003		LC068718.1-2004							
105								JX173086.1-2004		LC068713.1-1987							
105								JX173080.1-2001		LC068715.1-1994							

Supplementary Table S13: list of 117 recombination events identifed by RDP4 analysis package

		Breakpoint Positions									Detection Methods						
		In Alignment		In Recombinant Sequence		Relative to NC_001405.1-1953											
Recombination Event Number	Number In .RDP File	Begin	End	Begin	End	Begin	End	Recombinant Sequence(s)	Minor Parental Sequence(s)	Major Parental Sequence(s)	RDP	GENECONV	Bootscan	Maxchi	Chimaera	SiSscan	3Seq
105										LC068716.1-2003							
106	113~	19904*	19983	19746*	19825	19778*	19857	^AC_000008.1-1953	Unknown (JX173079.1-2002)	JX173078.1-2000	NS	NS	NS	NS	NS	9.66E-08	NS
107	114~	10121*	35243*	10075*	34608*	10057*	34732*	^HQ003817.1-2001	JX173079.1-2002	JX173082.1-2003	2.00E-03	NS	NS	NS	NS	5.74E-13	NS
107										AF534906.1-1953							
108	115~	6919*	8339*	6899*	8319*	6879*	8299*	^JX173082.1-2003	Unknown (JX173080.1-2001)	JX173078.1-2000	5.68E-04	1.38E-02	5.73E-04	NS	NS	NS	4.20E-04
108								AF534906.1-1953									
109	116~	27839*	28277*	27648*	28086*	27689*	28127*	^AC_000008.1-1953	Unknown (MF315029-2013)	AF534906.1-1953	2.03E-03	NS	6.28E-04	1.77E-02	8.69E-03	2.40E-05	NS
109									Unknown(KR699642.1-2009)	JX173082.1-2003							
109									Unknown(KX384959.1-2002)	JX173078.1-2000							
109										JX173083.1-2003							
109										JX173085.1-2003							
109										JX173086.1-2004							
110	117~	34350	34987*	33861	34497*	33840	34476*	^MF315029-2013	LC068713.1-1987	JX173079.1-2002	NS	NS	4.23E-02	NS	NS	NS	NS
110								KR699642.1-2009[T]									
110								JX173081.1-2001									
111	118~	18407	18724*	18328	18645*	18310	18627*	^HQ003817.1-2001	KF951595.1-2013	JX173078.1-2000	NS	NS	NS	NS	NS	7.32E-04	NS
111									FJ349096.1-1953	AF534906.1-1953							
111									JX423389.1-2007	JX173082.1-2003							
111									LC068717.1-2004	JX173080.1-2001							
111									LC068720.1-2005								
111									LC068718.1-2004								
111									LC068713.1-1987								
111									LC068714.1-1993								
111									LC068715.1-1994								
111									LC068716.1-2003								
112	120~	26947*	33272*	26809*	32833*	26801*	32763*	^JX173083.1-2003	MF315028-2012	Unknown (LC068713.1-1987)	NS	NS	NS	NS	NS	2.39E-26	NS

Supplementary Table S13: list of 117 recombination events identifed by RDP4 analysis package

		Breakpoint Positions															
		In Alignment		In Recombinant Sequence		Relative to NC_001405.1-1953					Detection Methods						
Recombination Event Number	Number In .RDP File	Begin	End	Begin	End	Begin	End	Recombinant Sequence(s)	Minor Parental Sequence(s)	Major Parental Sequence(s)	RDP	GENECONV	Bootscan	Maxchi	Chimaera	SiSscan	3Seq
112								AF534906.1-1953	KR699642.1-2009								
112								JX173085.1-2003	MF315029-2013								
112								JX173086.1-2004									
112								JX173080.1-2001									
113	121~	26947*	33272*	26808*	32831*	26801*	32763*	^JX173082.1-2003	JX173084.1-2003	Unknown (LC068713.1-1987)	NS	NS	NS	NS	NS	1.09E-17	NS
113									KR699642.1-2009								
113									KX384959.1-2002								
113									MF315029-2013								
114	125~	32922	33111	32428	32617	32416	32605	^AC_000008.1-1953	Unknown (KX384959.1-2002)	KF268129.1-2005	NS	NS	NS	NS	NS	3.89E-04	NS
115	129~	27756*	28752	27598*	28564	27607*	28579	^HQ003817.1-2001	Unknown (AF534906.1-1953)	KF268310.1-1992	NS	NS	NS	1.43E-03	NS	2.55E-06	2.78E-02
116	131~	1493	18508*	1484	18413*	1481	18411*	AC_000008.1-1953	Unknown (AF534906.1-1953)	LC068715.1-1994	NS	NS	NS	NS	NS	4.00E-06	1.70E-02
116										FJ349096.1-1953							
116										LC068717.1-2004							
116										LC068713.1-1987							
116										LC068714.1-1993							
117	132~	5553	6818*	5531	6796*	5513	6778*	KF268199.1-2008	JX173086.1-2004	LC068713.1-1987	3.15E-02	NS	2.87E-02	NS	NS	NS	NS

Table Key:

a: the first 40 recombination events were chosen for the breakpoint distribution analysis.

~ = It is possible that this apparent recombination signal could have been caused by an evolutionary process other than recombination.

* = The actual breakpoint position is undetermined (it was most likely overprinted by a subsequent recombination event).

^ = The recombinant sequence may have been misidentified (one of the identified parents might be the recombinant)

Minor Parent = Parent contributing the smaller fraction of sequence.

Major Parent = Parent contributing the larger fraction of sequence.

Unknown = Only one parent and a recombinant need be in the alignment for a recombination event to be detectable.
The sequence listed as unknown was used to infer the existance of a missing parental sequence.

Supplementary Table S13: list of 117 recombination events identifed by RDP4 analysis package

		Breakpoint Positions															
		In Alignment		In Recombinant Sequence		Relative to NC_001405.1-1953					Detection Methods						
Recombination Event Number	Number In .RDP File	Begin	End	Begin	End	Begin	End	Recombinant Sequence(s)	Minor Parental Sequence(s)	Major Parental Sequence(s)	RDP	GENECONV	Bootscan	Maxchi	Chimaera	SiSscan	3Seq

NS = No significant P-value was recorded for this recombination event using this method.

Supplementary Table S14: Edited annotation of NC_001405

Original GenBank annotation	Edited annotation [1]	Starts	Ends
E1A	E1A-e1	559	1111
E1A	E1A-e2	1226	1542
E1B	E1B-1	1711	2238
E1B	E1B-2	2016	3503
IX	IX	3600	4022
IVa2	IVa2-e2	4081	5417
E2B	E2B-1	5187	8774
L1	L1-1	7968	8381
E2B	E2B-2	8573	10579
L1	L1-2	11040	12287
L1	L1-3	12308	14065
L2	L2-1	14151	15866
L2	L2-2	15873	16469
L2	L2-3	16539	17648
L2	L2-4	17676	17918
L3	L3-1	18001	18753
L3	L3-2	18838	21744
L3	L3-3	21778	22392
E2A	E2A	22490	24079
L4	L4-1	24108	26525
L4	L4-2	26239	26826
L4	L4-3e2	26754	27127
L4	L4-4	27215	27898
E3A	E3A-1	27899	28222
E3A	E3A-2	28630	28815
E3A	E3A-3	28812	29291
E3A	E3A-4	29468	29773
E3B	E3B-1	29781	30056
E3B	E3B-2	30059	30451
E3B	E3B-3	30444	30830
U	U	30856	31019
L5	L5	31030	32778
E4	E4-1e2	32914	33192
E4	E4-2	33193	34077
E4	E4-3	33998	34342
E4	E4-4	34356	34706
E4	E4-5	34703	35095
E4	E4-6	35146	35532

1: exons are identified as follows, "-e1" for first exon, "-e2" for second exon. The genes of the same transcription unit are numbered from left to right.

Supplementary Table S15: breakpoint clustering test based on NC_001405 annotation

Test [1]	Breakpoints No in region	Breakpoint No outside region	breakpoints/100nts in region	breakpoints/100nts outside region	Probability of fewer than expected BPs in region	Probability of more than expected BPs in region
Intergenic regions vs all ORFs combined	0	55	0	0.168911786	1	0.012
E1A-e1 vs rest of ORFs	0	55	0	0.169432966	1	1
E1A-e2 vs rest of ORFs	0	55	0	0.168211081	1	0.95
E1B1 vs rest of ORFs	0	55	0	0.169492961	1	0.945
E1B2 vs rest of ORFs	1	54	6.56E-02	0.171484275	0.297	0.949
IX vs rest of ORFs	0	55	0	0.168757164	1	0.941
IVa2-e2 vs rest of ORFs	4	51	0.299177263	0.160998797	0.002	1
E2B1 vs rest of ORFs	5	50	0.13932063	0.169920502	0.009	0.997
L1-1 vs rest of ORFs	0	55	0	0.168710577	1	0.961
E2B2 vs rest of ORFs	2	53	9.72E-02	0.171202285	0.097	0.985
L1-2 vs rest of ORFs	4	51	0.320512821	0.160547748	0	1
L1-3 vs rest of ORFs	3	52	0.170478592	0.166376031	0.059	0.987
L2-1 vs rest of ORFs	0	55	0	0.175799562	1	0.41
L2-2 vs rest of ORFs	0	55	0	0.169662926	1	0.807
L2-3 vs rest of ORFs	0	55	0	0.172476747	1	0.557
L2-4 vs rest of ORFs	0	55	0	0.167830289	1	0.954
L3-1 vs rest of ORFs	4	51	0.530877817	0.158086839	0.006	0.998
L3-2 vs rest of ORFs	7	48	0.234899696	0.15981684	0.996	0.014
L3-3 vs rest of ORFs	3	52	0.487804878	0.160497698	0.019	0.999
E2A vs rest of ORFs	2	53	0.125786164	0.168659454	0.593	0.664
L4-1 vs rest of ORFs	4	51	0.164511184	0.166760342	0.158	0.941
L4-2 vs rest of ORFs	0	55	0	0.169753284	1	0.708
L4-3e2 vs rest of ORFs	1	54	0.251126533	0.16556097	0.224	0.968
L4-4 vs rest of ORFs	2	53	0.291531248	0.163943675	0.127	0.977
E3A-1 vs rest of ORFs	4	51	1.234448826	0.15601019	0.003	1
E3A-2 vs rest of ORFs	0	55	0	0.16765939	1	0.513
E3A-3 vs rest of ORFs	2	53	0.412149737	0.162931887	0.503	0.747
E3A-4 vs rest of ORFs	0	55	0	0.168201129	1	0.214
E3B-1 vs rest of ORFs	0	55	0	0.168009789	1	0.407
E3B-2 vs rest of ORFs	0	55	0	0.168714664	1	0.094
E3B-3 vs rest of ORFs	0	55	0	0.168572117	1	0.063
U vs rest of ORFs	0	55	0	0.167426701	1	0.47
L5 vs rest of ORFs	5	50	0.271244765	0.160393088	0.999	0.002
E4-1e2 vs rest of ORFs	1	54	0.358422939	0.164960033	0.168	0.978
E4-2 vs rest of ORFs	1	54	0.11299435	0.168071244	0.371	0.93
E4-3 vs rest of ORFs	0	55	0	0.168354264	1	0.904
E4-4 vs rest of ORFs	1	54	0.284671999	0.165325083	0.071	0.998
E4-5 vs rest of ORFs	0	55	0	0.168612385	1	0.951
E4-6 vs rest of ORFs	0	55	0	0.168570971	1	0.977
End 50% vs Middle 50%	31	25	0.181580556	0.146897884	0.07	0.966
End 25% vs Middle 75%	20	36	0.234797762	0.140773644	0.002	0.999
End 10% vs Middle 90%	9	47	0.264186251	0.153173045	0.001	1

1: tests with p-value <0.01 are highlighted.

Supplementary Figure 1: Phylogenetic network built with 32 HAdV-C WGSs. The clusters corresponding to type 1, 2, 5 and 6 are shown with different colors, type 1 in pastel pink, type 2 in blue, type 5 in pastel yellow and type 6 in pastel green. The sequences corresponding to the prototype viruses of type 1, 2, 5 6 and 57 are noted by a black dot.

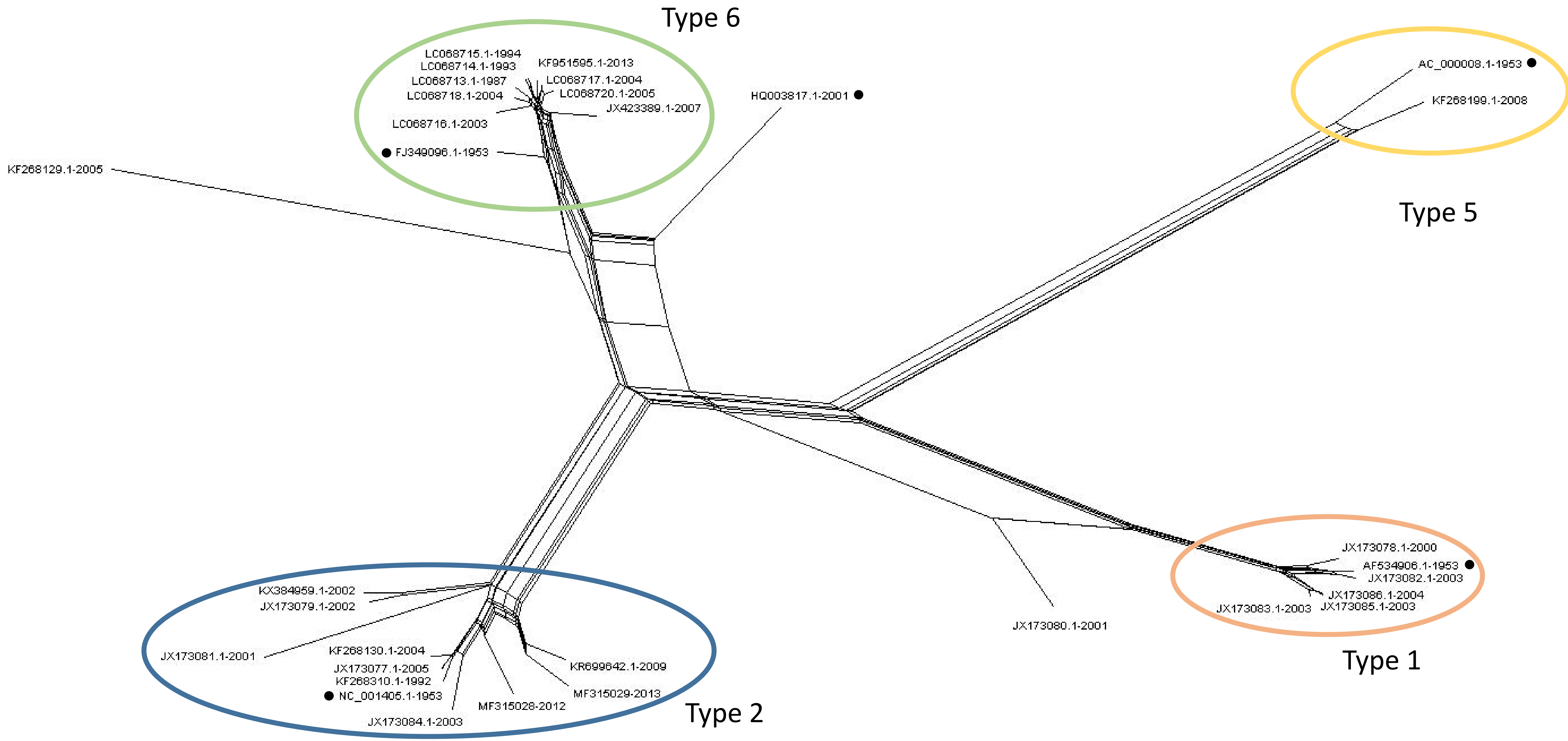

Supplementary Figure 1

Supplementary Figure 2: Neighbor joining phylogenetic trees of 32 HAdV-Cs. WGS as well contiguous regions were analyzed. The genomic region used to build the tree is shown for each tree. Type designations for the hexon and fiber genes are indicated by brackets. The related sequences are identified with symbols: the pair LC068714-5 with an asterisk, the triplet LC068717-20-KF951595 with a triangle, the triplet KF268310-130-JX173077 with a circle and the triplet JX173083-5-6 with a square. The most recent sequence in each triplet is identified by a black symbol whereas the other two sequences are identified with an empty symbol. The related sequences were identified based on the p-distances from supplementary table 1. Bootstrap values greater than 70% are shown.

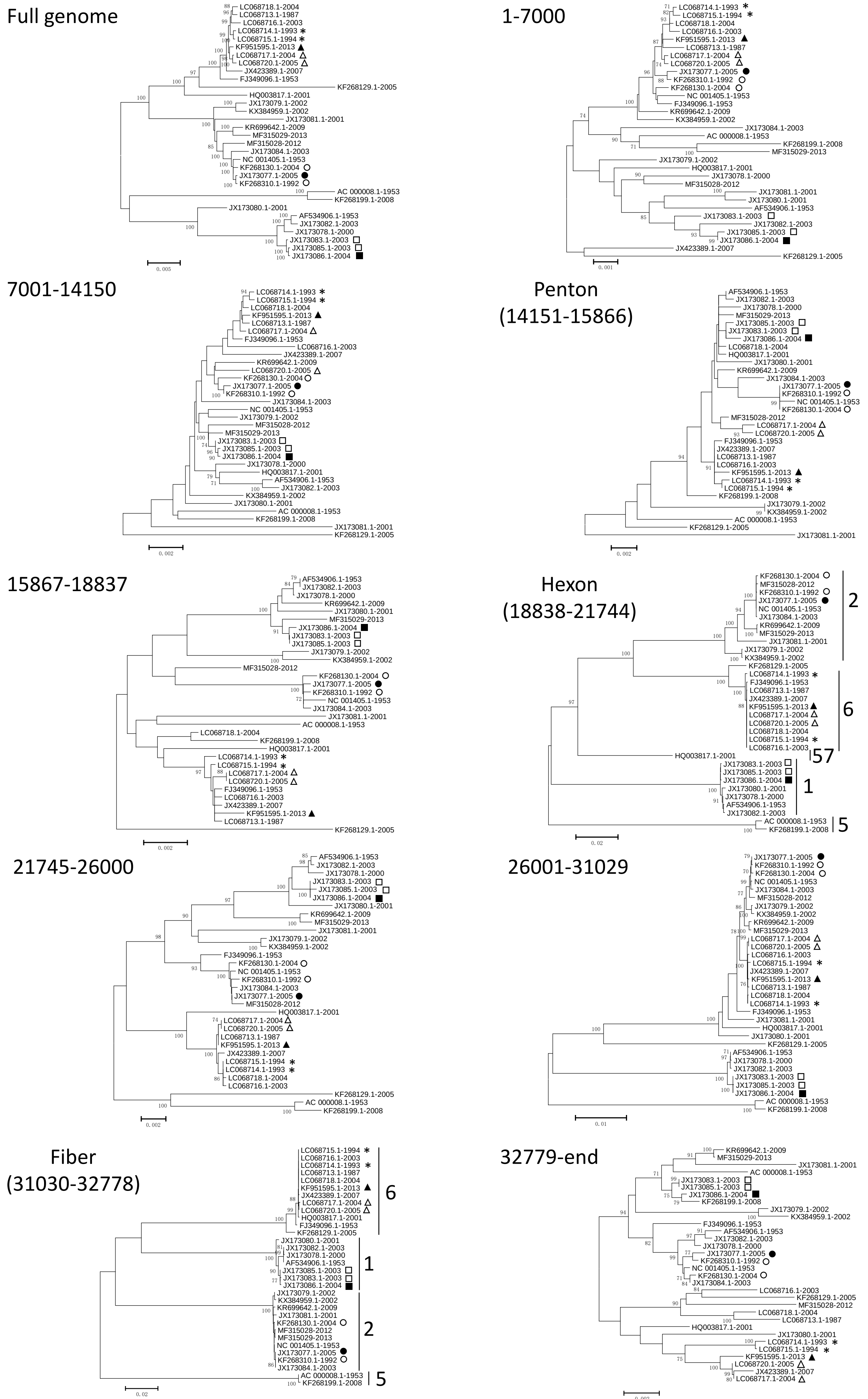

Supplementary Figure 2

Supplementary Figure 3: Phylogenetic network built with 21 HAdV-C WGSs. The clusters corresponding to type 1, 2, 5 and 6 are shown with different colors, type 1 in pastel pink, type 2 in blue, type 5 in pastel yellow and type 6 in pastel green. The sequences corresponding to the prototype viruses of type 1, 2, 5 and 6 are noted by a black dot.

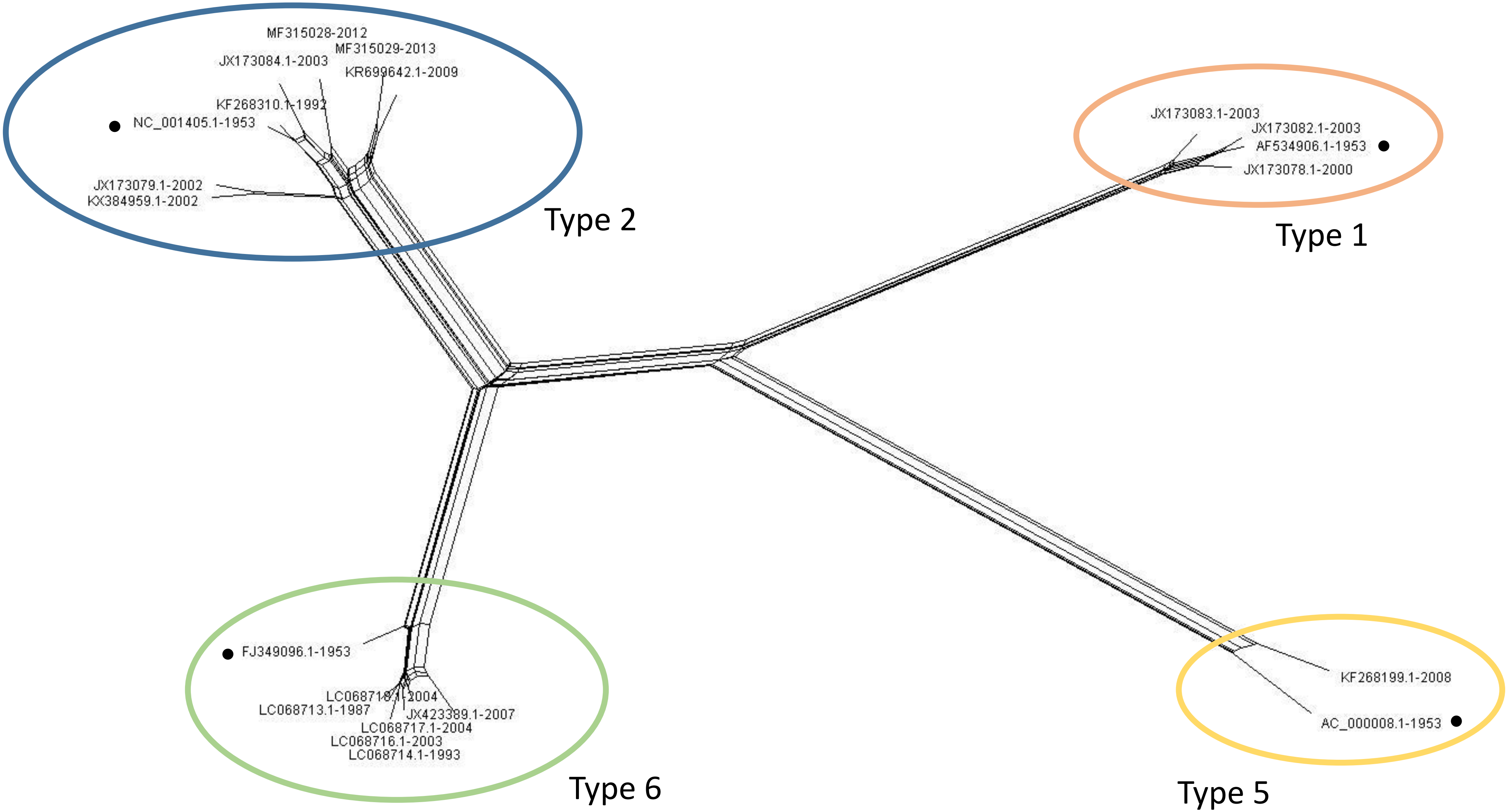

Supplementary Figure 3

Supplementary figure S4: Algorithm to analyze the lowest pairwise p-distances. The lowest pairwise distances are analyzed at the WGS level as well as the genomic region level. The analysis at WGS level identifies the backbone, either known or unknown. The analysis at the genomic region level identifies whether the genome is a recombinant and the genetic elements involved in any recombination. The recombination predicted by this analysis are manually checked on phylogenetic trees and assessed with RDP4.

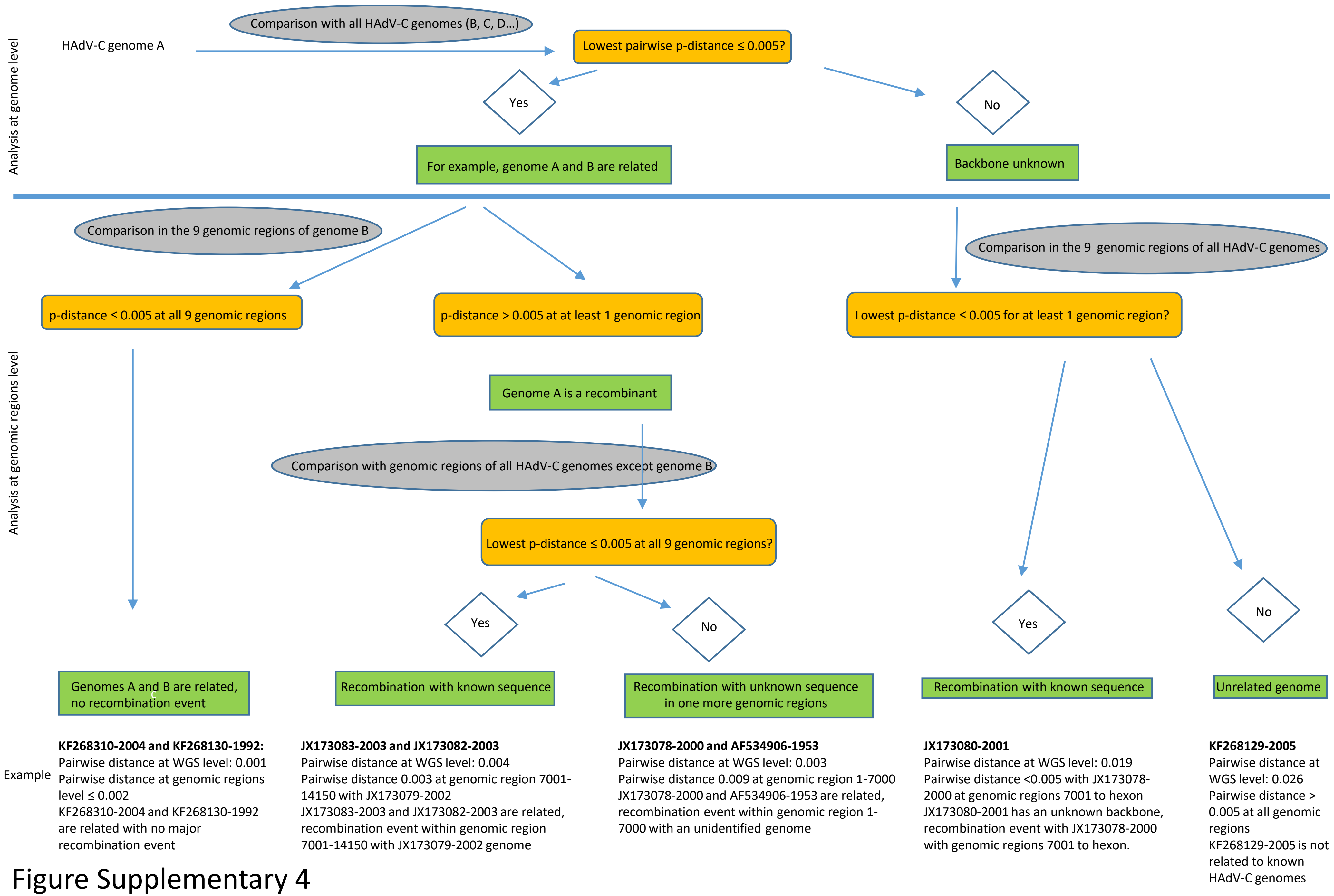

Figure Supplementary 4

Supplementary Figure 5: Neighbor joining phylogenetic trees of WGS from 32 HAdV-Cs and 2 non-human adenoviruses. HAdV-C sequences are shown in red. The sequences from chimpanzee (CS138463) and bonobo (HC191035) are shown in black. Bootstrap values greater than 70% are shown.

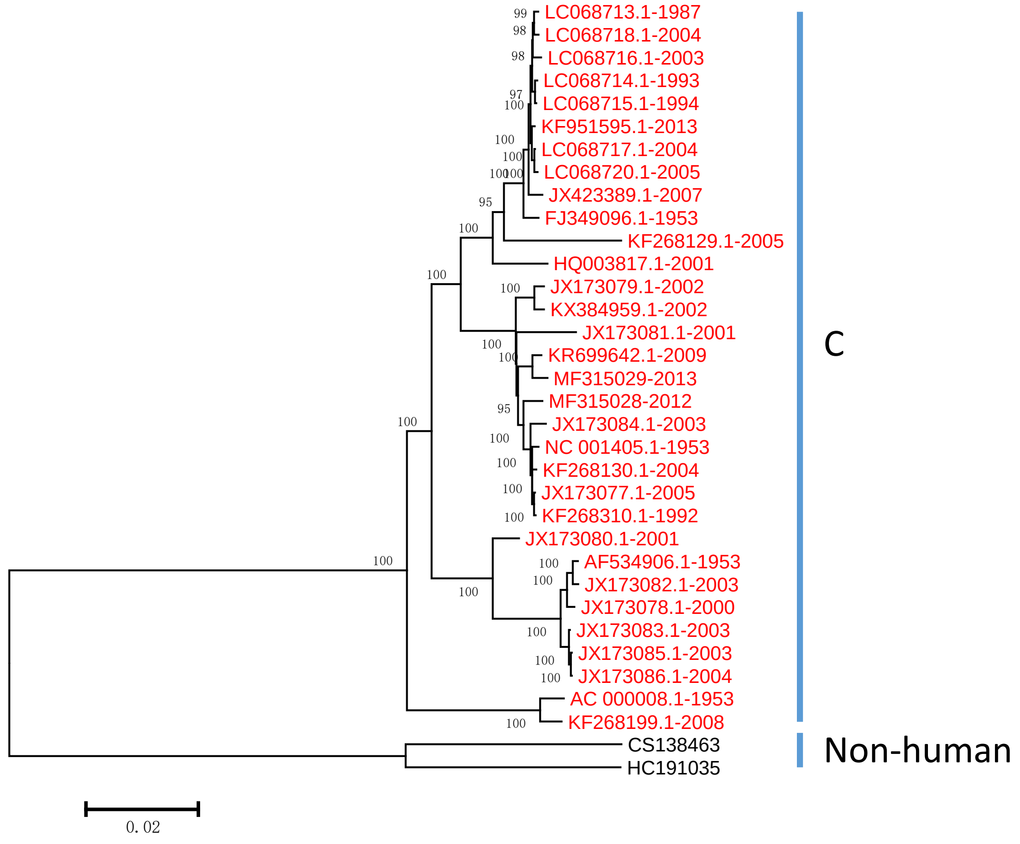

Supplementary figure 5

Supplementary Figure 6: Neighbor joining phylogenetic trees of WGS from 32 HAdV-Cs and 76 HAdV from species A, B, D, E, F and G. HAdV-C sequences are shown in red. The species are shown in brackets. Bootstrap values greater than 70% are shown.

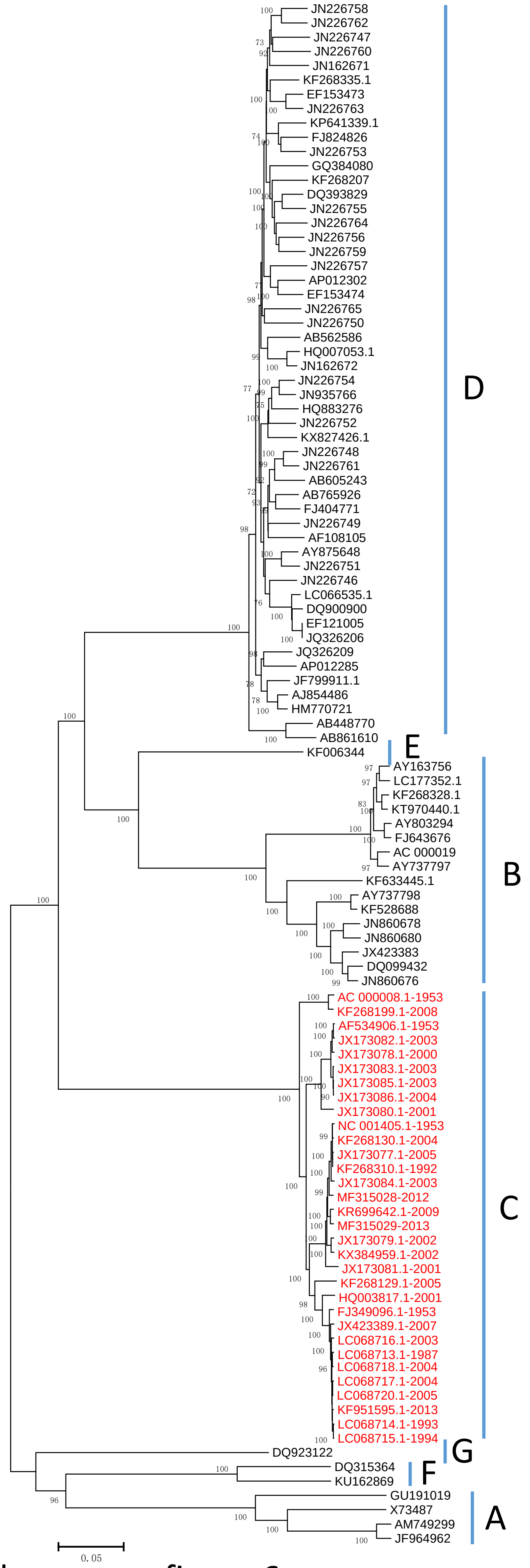

Supplementary figure 6
